# PI3K regulates TAZ/YAP and mTORC1 axes that can be synergistically targeted

**DOI:** 10.1172/jci.insight.191600

**Published:** 2026-02-10

**Authors:** Keith C. Garcia, Ali A. Khan, Krishnendu Ghosh, Souradip Sinha, Nicholas Scalora, Gillian DeWane, Colleen Fullenkamp, Nicole Merritt, Yuliia Drebot, Samuel Y. Yu, Mariah Leidinger, Michael D. Henry, Patrick J. Breheny, Michael S. Chimenti, Munir R. Tanas

**Affiliations:** 1 Department of Pathology,; 2 Cancer Biology Graduate Program,; 3 Center for Biocatalysis and Bioprocessing Program,; 4 Molecular Medicine Graduate Program,; 5 Department of Molecular Physiology and Biophysics,; 6 Holden Comprehensive Cancer Center,; 7 Department of Biostatistics,; 8 Iowa Institute of Human Genetics, Carver College of Medicine, University of Iowa,; 9 Pathology and Laboratory Medicine, Veterans Affairs Medical Center, Iowa City, Iowa, USA.

**Keywords:** Cell biology, Oncology, Cancer, Signal transduction

## Abstract

Sarcomas are a heterogeneous group of cancers with few shared therapeutic targets. We show that PI3K signaling is frequently activated in sarcomas due to PTEN loss (in 30%–60%), representing a common therapeutic target. The PI3K pathway has lacked a downstream oncogenic transcription factor. We show TAZ and YAP are transcriptional coactivators regulated by PI3K and drive a transcriptome necessary for tumor growth in a PI3K-driven sarcoma mouse model. This PI3K/TAZ/YAP axis exists in parallel to the known PI3K/AKT/mTORC1 axis, providing a rationale for combination therapy targeting the TAZ/YAP-TEAD interaction and mTORC1. Combination therapy using IK-930 (TEAD inhibitor) and everolimus (mTORC1 inhibitor) synergistically diminished proliferation and anchorage-independent growth of PI3K-activated sarcoma cell lines at low, physiologically achievable doses. Furthermore, this combination therapy showed a synergistic effect in vivo, suggesting that an integrated view of PI3K and Hippo signaling can be leveraged therapeutically in PI3K-activated sarcomas.

## Abstract 2

PI3K signaling is active in sarcomas of different histological types. A PI3K-TAZ/YAP axis exists in addition to the PI3K-Akt-mTORC1 axis that can be synergistically targeted.

## Graphical abstract

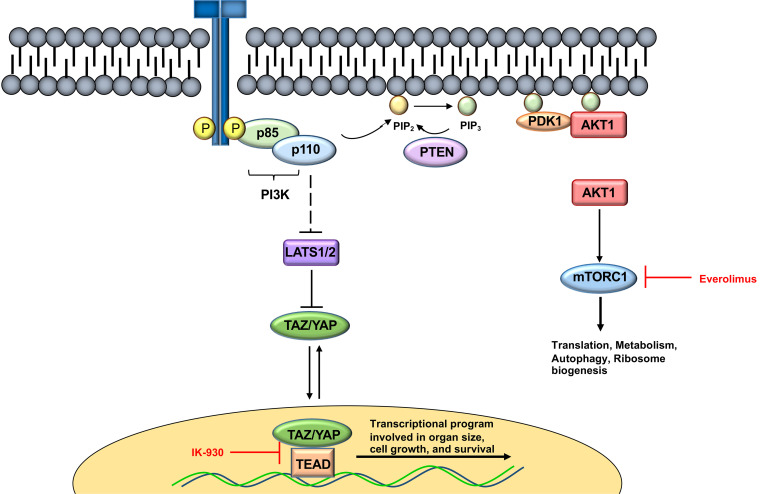

## Introduction

Soft tissue sarcomas are a heterogeneous group of cancers that consist of over 50 histological subtypes (1), making these cancers challenging to both diagnose and treat. Additional therapeutic targets active in different histological types of sarcomas are needed. Phosphatidylinositol-3 kinase (PI3K) signaling has been implicated in the pathogenesis of several different sarcoma types (2–4), but as a group, sarcomas have not been identified as a cancer type in which PI3K signaling plays a central role or can be successfully targeted. This is because in contrast to other cancers such as breast cancer or ovarian cancer, there is a scarcity of mutations in *PIK3CA*, *AKT1*, *AKT2*, or *AKT3* (5). In addition, the PI3K/mTORC1 axis has proven to be a difficult therapeutic target in cancer (6), and targeting mTORC1 has shown modest efficacy in treating sarcomas in phase III clinical trials (7). Thus, targeting the PI3K/mTORC1 axis represents a therapeutic approach in sarcomas that could be improved upon.

There are 3 classes of PI3K enzymes, of which the class I enzymes are the best characterized (8). Class I PI3Ks are heterodimers consisting of a catalytic subunit (p110) and a regulatory subunit (p85). When activated by growth factor receptor tyrosine kinases, PI3K enzymes will phosphorylate PI(4,5)P_2_ (PIP_2_) to generate PI(3,4,5)P_3_ (PIP_3_) at the cell membrane that results in a docking site for phosphoinositide-dependent protein kinase-1 (PDK1) and AKT via their individual pleckstrin homology (PH) domains. Upon phosphorylation of AKT by PDK1, AKT phosphorylates a number of substrates, including GSK-3β, p21, BAD, FOXO1, MDM2, and TSC2, which coordinately drives proliferation, inhibits apoptosis, and drives growth (9–16). By inactivating the TSC1/TSC2 complex, mTOR is activated as part of the mTORC1 complex that is also composed of the RPTOR and GβL proteins. The mTORC1 complex subsequently phosphorylates and activates an S6K1-S6 axis and 4E-BP1, which together stimulate cap-dependent translation of proteins (17–20). A key negative regulator of the PI3K pathway is the phosphatase and tensin homolog (PTEN) tumor suppressor that dephosphorylates PIP_3_, converting it back to PIP_2_ at the membrane, thereby dampening PI3K signaling (21–23).

One underappreciated phenotype of PI3K signaling is the regulation of cell size (24), a phenotype that overlaps with that of the Hippo pathway (25). Indeed, several connections have been made between the PI3K signaling pathway and the Hippo pathway as it pertains to amino acid regulation (26–29) but also at a signal transduction level (30–35).

The Hippo pathway is a highly conserved pathway involved in regulating tissue/organ and cell size (36–41). This pathway is a core serine/threonine kinase cascade that consists of the mammalian STE20-like protein kinases 1 and 2 (MST1/2) and the large tumor suppressor kinases 1 and 2 (LATS1/2) (36, 37, 39–44). The downstream effectors of the Hippo pathway are the transcriptional coactivators TAZ (gene name is *WWTR1*; WW domain-containing transcription regulator 1) and YAP (gene name is *YAP1*; Yes-associated protein 1). TAZ and YAP have been implicated as oncoproteins in a number of cancers including breast, colon, liver, lung, pancreas, and thyroid cancers (45). More recently, TAZ and YAP have been shown to be frequently activated in sarcomas (46–58). Various external cues such as cell confluence or detachment can activate the core signaling cascade in the Hippo pathway, leading to LATS1/2-mediated phosphorylation of TAZ and YAP at multiple serine residues that promote their accumulation into the cytoplasm, where they are subsequently ubiquitinated and degraded by the proteasome (40, 41, 59, 60). Inactivation of the core serine/threonine kinase cassette results in the nuclear accumulation of TAZ and YAP where they bind to the TEA domain (TEAD) family of transcription factors to stimulate target gene expression (61, 62). Upstream signaling pathways such as RAS/RAF/MEK/ERK and WNT signaling (63, 64) have been implicated to activate TAZ and YAP in various cancer types (65, 66).

One notable gap in the known PI3K signaling cascade and an enigma in the field is the absence of downstream oncogenic transcription factors that could transduce signals from the cell membrane into the nucleus (FOXO1 is a transcription factor downstream of PI3K but has a tumor suppressive function) (67). Because TAZ and YAP are transcriptional coactivators, we hypothesized they could be serving as a nexus linking PI3K signaling and the Hippo pathway. Herein, we show using in vitro and in vivo approaches that TAZ and YAP are oncogenic transcriptional coactivators and effectors downstream of PI3K signaling, and that this finding can be leveraged to better therapeutically target PI3K signaling in sarcomas.

## Results

### PI3K is activated in sarcomas.

**Figure 1:**
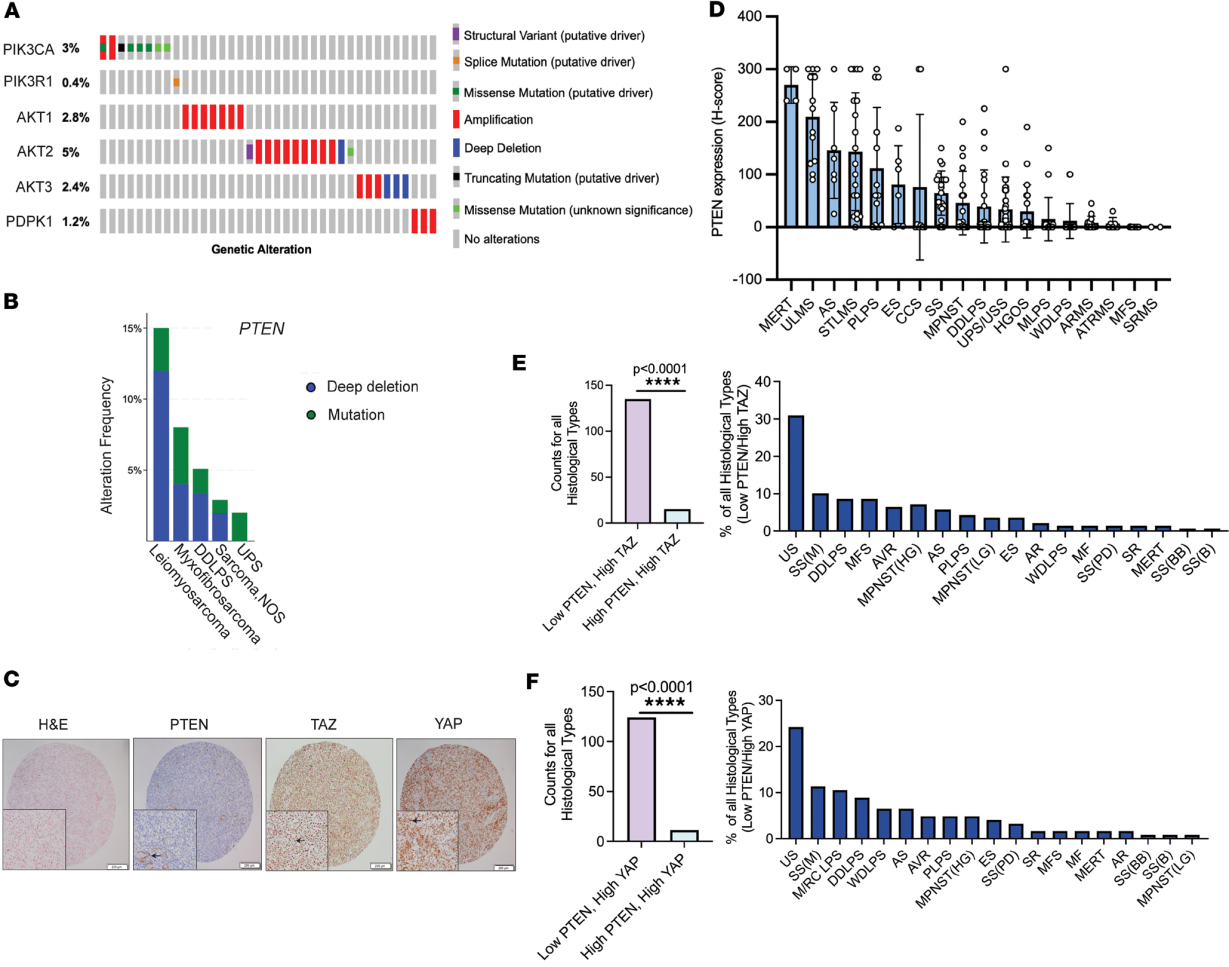
PI3K is widely activated in sarcomas. (**A**) The Cancer Genome Atlas (TCGA) data (cBioPortal online) for PI3K signaling alterations in sarcomas (*n* = 253) demonstrating the incidence of mutations in the PI3K signaling pathway. (**B**) TCGA data demonstrating the frequency of genetic alterations of *PTEN* in sarcomas (*n* = 253). (**C**) H&E and IHC performed for PTEN, TAZ, and YAP on adult-type rhabdomyosarcoma from tissue microarray. Inset for PTEN shows vascular positive control. Insets for activated (nuclear) TAZ and YAP. (**D**) H-scores from clinical tissue microarray panel of various sarcoma histological subtypes. See supplemental materials for abbreviations used. Histology, H&E staining, and IHC for PTEN, TAZ, and YAP in a sample of alveolar rhabdomyosarcoma (ARMS). (**E**) Number of sarcomas exhibiting low PTEN/high TAZ expression relative to high PTEN/high TAZ expression (left) and composition of sarcomas exhibiting low PTEN/high TAZ profile according to histological type (right). (**F**) Number of sarcomas exhibiting low PTEN/high YAP expression relative to high PTEN/high YAP expression (left) and composition of sarcomas exhibiting low PTEN/high YAP profile according to histological type (right). Two-tailed *P* values for IHC counts were evaluated using the χ^2^ test with 1 degree of freedom. **P < 0.01, ***P < 0.001.

To evaluate the mutational landscape of the PI3K signaling pathway, we analyzed publicly available datasets (The Cancer Genome Atlas) (68-70) for genetic alterations involving the pathway’s core components such as *PIK3CA* (p110α subunit)*, PIK3R1* (p85 subunit), *AKT1-3,* and *PDPK1* (PDK1). We observed that 5% or fewer of sarcomas exhibit gain-of-function genetic alterations for each of these members of the PI3K signaling pathway (Figure 1A). Scattered mutations in PTEN have been identified in various cancers, including the loss-of-function R130Q mutation in cancers including undifferentiated pleomorphic sarcoma (71–73) (Supplemental Figure 1A; supplemental material available online with this article; https://doi.org/10.1172/jci.insight.191600DS1). Further analysis of data from TCGA showed alterations of *PTEN* such as deletions and truncations (Figure 1B). To determine whether PTEN was lost at the protein level in clinical samples, we performed IHC on a tissue microarray composed of 144 untreated sarcomas encompassing 18 histological types (Figure 1, C and D). H-scores were calculated (intensity × % positive cells) to quantitate PTEN expression. Sarcomas demonstrated a spectrum of PTEN loss, including those with complete loss of expression (H-score of 0) and samples with very low expression (H-score of ≤50). Approximately 1/3 (32%) of sarcomas showed a complete loss of PTEN expression, consistent with the 20%–30% loss that has been reported in the literature (2). However, approximately 2/3 of sarcomas (63%) showed complete loss to very low expression of PTEN (H-score of ≤50) across different histological types, suggesting that PI3K may be activated in a higher percentage of sarcomas than previously appreciated.

To determine whether PTEN expression and thus PI3K activation correlates with TAZ and YAP expression and activation (nuclear localization), H-scores for TAZ and YAP from a previously published dataset (48) were plotted as a function of H-score for PTEN. The resulting scatter plot for TAZ (Supplemental Figure 1B) was divided into 4 quadrants/categories: TAZ high/PTEN low, TAZ high/PTEN high, TAZ low/PTEN low, and TAZ low/PTEN high. The scatter plot for YAP was similarly divided (Supplemental Figure 1C). The cutoff for low expression of TAZ, YAP, and PTEN was established at an H-score of 150 or less (the median H-score possible); H-scores of more than 150 were considered high. Tumors with high TAZ expression/activation were more likely to demonstrate low PTEN expression than high PTEN expression (*P* < 0.0001) (Figure 1E). Similarly, tumors with high YAP expression/activation were more likely to demonstrate low PTEN expression than high PTEN expression (*P* < 0.0001) (Figure 1F). The sarcoma histological type with the highest percentage of low PTEN/high TAZ tumors and low PTEN/high YAP tumors was undifferentiated sarcoma (composed of undifferentiated pleomorphic sarcoma and undifferentiated spindle cell sarcoma). Other histological types notable for having a relatively high percentage of low PTEN/high TAZ/YAP tumors included monophasic synovial sarcomas, well-differentiated/dedifferentiated liposarcoma, myxoid/round cell liposarcoma, malignant peripheral nerve sheath tumor, myxofibrosarcoma, and alveolar rhabdomyosarcoma (Figure 1, E and F).

We subsequently assayed 8 sarcoma cell lines (Supplemental Figure 1D) to examine hyperactive PI3K signaling by Western blot after serum starvation. Phosphorylated AKT (S473) levels were elevated for SJCRH30 (alveolar rhabdomyosarcoma) and RD (embryonal rhabdomyosarcoma) cell lines compared with the primary skeletal muscle cell control (Supplemental Figure 1D). Phosphorylated AKT (S473) was also elevated for the A204 (malignant extrarenal rhabdoid tumor) and SW872 (liposarcoma) cell lines compared with primary cell controls that were available (primary smooth muscle and skeletal muscle cells). Consistent with findings from the above-mentioned clinical samples (Figure 1, C and D), 3 of the 4 cell lines (75%) mentioned above demonstrated elevated phospho-AKT levels and were simultaneously characterized by reduced PTEN expression. To determine whether these cell lines were dependent on PI3K for cell survival and thus driven by PI3K, clonogenic outgrowth assays were performed on the SJCRH30 (PTEN lost) and A204 (PTEN intact) sarcoma cell lines. Treatment with the pan–class I PI3K inhibitors, GDC-0941 (Pictilisib) and LY294002 (LY) (Supplemental Figure 1, E–H), resulted in a significant decrease in cell survival in a concentration-dependent manner. Even so, inhibition did not entirely prevent clonogenic outgrowth even at higher concentrations, providing an impetus to better target PI3K signaling in these cell lines and sarcomas overall.

### TAZ/YAP are transcriptional end effectors regulated by PI3K.

**Figure 2:**
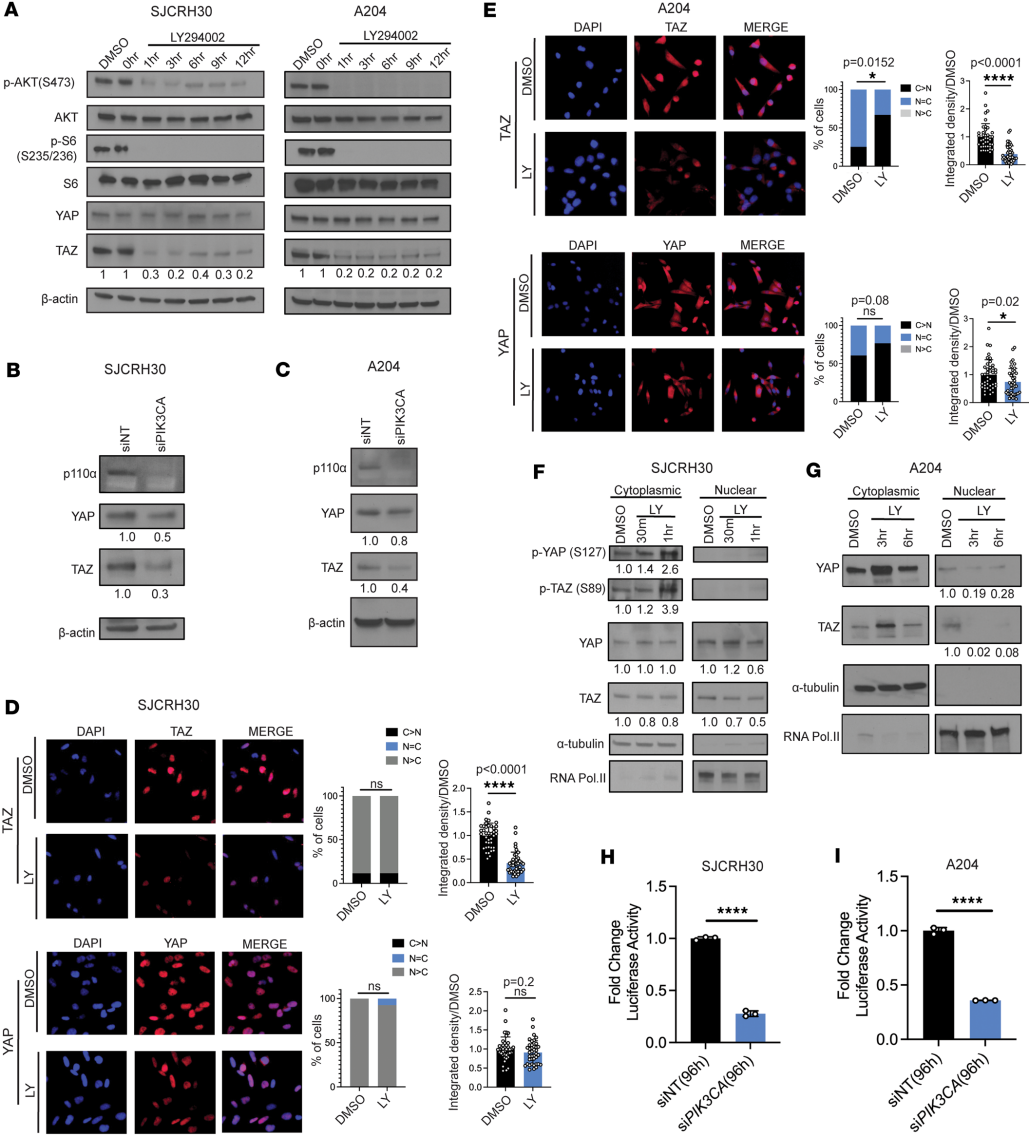
PI3K inhibition alters the stability and localization of TAZ/YAP in SJCRH30 and A204 cells. (**A**) Western blot of SJCRH30 and A204 cells treated with 30 μM LY294002 for the indicated time points. (**B** and **C**) Western blot of TAZ/YAP upon siRNA-mediated knock down of *PIK3CA* (p110α) in SJCRH30 (**B**) and A204 (**C**) cells. (**D** and **E**) Immunofluorescence analysis of SJCRH30 (**D**) or A204 (**E**) cells that were treated with 60 μM LY2094002 for 3 hours and 6 hours, respectively (see Methods for details). (**F** and **G**) Nuclear and cytoplasmic fractionation of SJCRH30 cells (**F**) and A204 cells (**G**) treated with vehicle (DMSO) or 60 μM LY294002. Luciferase activity measured upon siRNA (pooled) mediated knockdown of *PIK3CA* for 96 hours in (**H**) SJCRH30 cells and (**I**) A204 cells stably expressing a TEAD luciferase reporter. Statistical analysis was performed using unpaired 2-tailed Student’s *t* test. Each experiment was repeated at least twice. Data are shown as mean ± SD. *P < 0.05, ****P < 0.0001, ns not statistically significant. Each experiment was repeated at least twice.

Observations from analysis of the above clinical samples (Figure 1, E and F) led to the hypothesis that PTEN loss leads to activation of PI3K signaling, which subsequently activates TAZ and YAP in sarcomas. To determine whether TAZ and YAP were oncoproteins in the PI3K-activated SJCRH30 and A204 cell lines (Supplemental Figure 1D), we performed shRNA-mediated knockdown of TAZ or YAP in these cell lines, which resulted in reduced anchorage-independent growth (Supplemental Figure 1, I and J). These results demonstrated that SJCRH30 and A204 were two PI3K-activated sarcoma cell lines in which TAZ and YAP functioned as oncoproteins. To determine whether TAZ and YAP are regulated by PI3K signaling, SJCRH30 and A204 cells were treated with 30 μM of LY, a pan–class I PI3K inhibitor, over a 12-hour time course. For both the SJCRH30 and A204 cell lines, we saw marked decreases in total TAZ levels beginning at 1 hour of treatment (Figure 2A) that exceeded the decrease of expression of TAZ upon serum starvation in the SJCRH30 and A204 cell lines (with half-lives of 1 hour and 4 hours, respectively; see Supplemental Figure 2, A and B) (74). Total YAP protein expression levels remained essentially unchanged in both SJCHR30 and A204 cells (Figure 2A) with 30 μM of LY.

To further determine whether PI3K regulates TAZ/YAP in sarcoma cells, we performed siRNA-mediated knockdown of *PIK3CA*, the catalytic subunit of the PI3K heterodimer, that resulted in a substantial decrease in total TAZ protein levels, and to a lesser extent YAP, in both SJCRH30 and A204 cell lines (Figure 2, B and C). YAP is hypothesized to be more stable than TAZ due to additional serine residues involved in its protein stability (41, 59, 60). To test the hypothesis that PI3K signaling was affecting the localization of YAP rather than total levels of YAP, we performed immunofluorescence analysis on SJCHR30 cells treated with 60 μM LY (Figure 2D). Although the ratio of nuclear/cytoplasmic TAZ did not change after treatment with LY, total TAZ levels decreased after treatment with LY by integrated density (*P* < 0.0001) (Figure 2D). No significant change in YAP localization was identified after treatment with 60 μM LY. When A204 cells were treated with LY, localization of TAZ was shifted from the nucleus into the cytoplasm (Figure 2E). A similar trend toward cytoplasmic localization was noted for YAP; however, this was not statistically significant. Total TAZ and YAP expression, as calculated by integrated density, was decreased in A204 cells after treatment with LY (Figure 2E).

Although a shift in the nuclear to cytoplasmic localization of YAP was not identified by immunofluorescence in the SJCRH30 cells, nuclear and cytoplasmic fractionation demonstrated a decrease in total YAP in the nuclear compartment after treatment with 60 μM LY for 1 hour accompanied by a greater than 2-fold increase in phospho-YAP S127 in the cytoplasmic compartment (Figure 2F). In addition, nuclear and cytoplasmic fractionation showed that total TAZ protein levels decreased between 30 minutes and 1 hour, and a greater than 3-fold increase in phosphorylated TAZ (S89) was observed in the cytoplasmic fraction (Figure 2F). Consistent with the above findings, PI3K inhibition in A204 cells revealed that TAZ and YAP expression decreased in the nuclear fraction after treatment with LY relative to the DMSO control (Figure 2G).

The above studies showed that PI3K regulates the expression and localization of TAZ and YAP. To determine whether this inhibition of PI3K signaling was functionally important and affected the transcriptional activity of TAZ/YAP, SJCRH30 and A204 cells stably expressing an 8xTEAD luciferase reporter were subsequently transfected with siRNA targeting *PIK3CA* for 96 hours using a dual luciferase reporter assay with *Renilla* luciferase control (see Methods for details). siRNA-mediated knockdown of *PIK3CA* in the 2 cell lines caused a significant reduction in TEAD reporter activity; a 3.6-fold reduction (*P* < 0.0001) was noted for SJCRH30, and a 2.9-fold reduction (*P* < 0.0001) was seen in the A204 cell line (Figure 2, H and I). These findings demonstrate that inhibition of PI3K signaling leads to reduced transcriptional activity of TAZ/YAP in sarcoma cells. Collectively, the above data indicate TAZ/YAP are transcriptional end effectors regulated by PI3K in sarcoma cell lines.

### PI3K inhibition results in LATS-mediated regulation of TAZ and YAP in sarcoma cells.

To determine how PI3K may be regulating the expression and activity of TAZ and YAP, we examined the role of core enzymes downstream of PI3K. Work from others has suggested that PDK1 and AKT are key regulators of TAZ and YAP (33, 35, 75). To determine whether PDK1 was a regulator of TAZ/YAP in these 2 cell lines, we performed siRNA-mediated silencing of *PDK1* and found that it did not affect total TAZ or YAP expression levels (Supplemental Figure 2C). To determine whether AKT is a potential upstream regulator of TAZ and YAP in SJCRH30 and A204 cells, we performed siRNA-mediated knockdown of *AKT1* (Supplemental Figure 2D) and treatment with MK2206, an allosteric pan-AKT inhibitor (Supplemental Figure 2, E and F). Pharmacological and genetic inhibition of AKT had no effect on the expression of TAZ and YAP protein levels in both cell lines (Supplemental Figure 2, D–F). The above data indicate PDK1 and AKT are not regulators of TAZ and YAP expression in these PI3K-activated sarcoma cell lines.

There is some evidence suggesting that GSK3β may regulate TAZ by phosphorylating serine residues 58 and 62 that are part of the N-terminal phosphodegron of TAZ (76) (Supplemental Figure 3A). To determine whether GSK3β plays a role in TAZ stability subsequent to PI3K inhibition, SJCRH30 and A204 cells were engineered to stably express WT Flag-TAZ or the Flag-TAZ S58/62A GSK3β–resistant mutant (Supplemental Figure 3, A and B). When treated with LY294002, Flag-TAZ and Flag-TAZ S58/62A both decreased over the course of 1 hour in SJCRH30 cells, suggesting that TAZ is regulated by PI3K in a GSK3β-independent manner (Supplemental Figure 3, C and D). Similarly, Flag-TAZ and Flag-TAZ S58/62A in A204 cells decreased over time when treated with LY (Supplemental Figure 3, E and F). Taken together, the above findings indicate that PI3K is regulating TAZ and YAP by a mechanism that does not include PDK1, AKT, or GSK3β.

**Figure 3:**
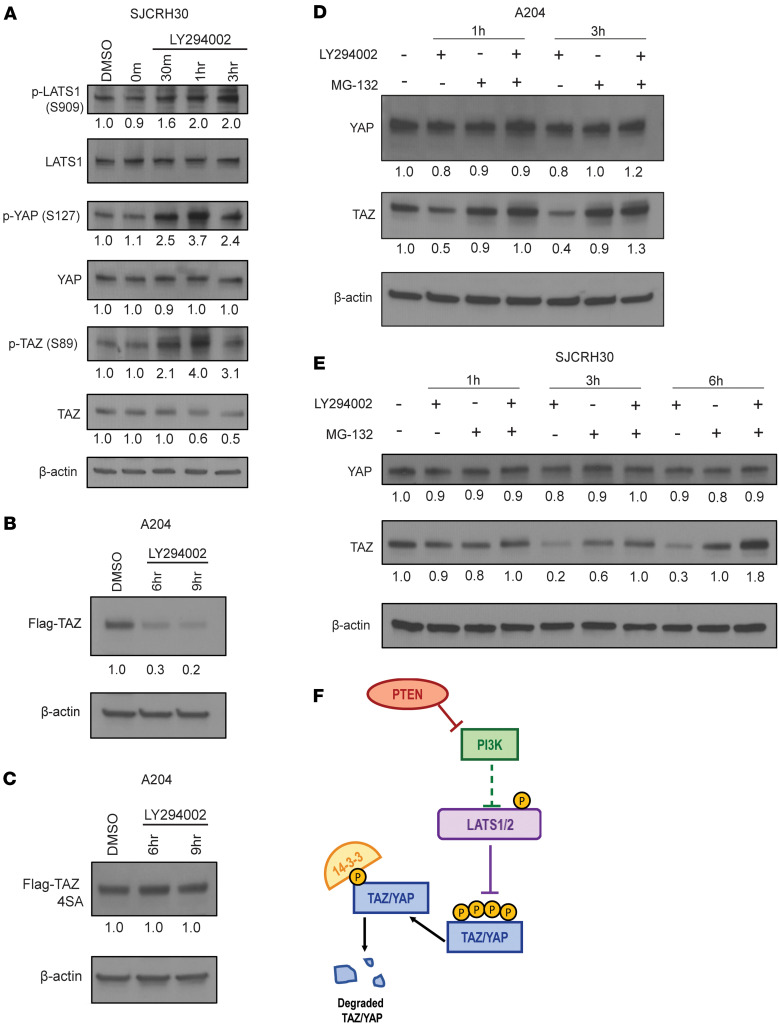
PI3K inhibition promotes LATS-mediated degradation of TAZ/YAP in SJCRH30 and A204 cells. (**A**) Western blot of SJCRH30 cells that were treated with 60 μM LY294002 for the indicated time points. (**B**) Flag-TAZ or (**C**) Flag-TAZ 4SA were treated with 60 μM LY294002 for 6 and 9 hours. (**D**) A204 cells were treated with or without LY294002 (60 μM) or MG132 (10 μM) as indicated for 6 and 9 hours. (**E**) SJCRH30 cells were treated with or without LY294002 or MG132 as indicated at 1, 3, and 6 hours. (**F**) Working model. Each experiment was repeated at least twice.

To gain further insight into how TAZ and YAP are regulated by PI3K, we evaluated whole-cell lysates of SJCRH30 after treatment of LY by Western blot (Figure 3A). Consistent with previous observations (Figure 2F), 30 minutes to 1 hour after treatment with LY, elevated phosphorylation levels of TAZ (S89) and YAP (S127), target sites for the LATS1/2 kinases, were observed relative to cells treated with DMSO (vehicle) (Figure 3A). In addition, phospho-LATS1 (S909) was elevated after treatment with LY, consistent with activation of LATS1 (77, 78). Quantitative immunoblot analysis showed that total TAZ protein levels decreased approximately 2-fold relative to vehicle control around 3 hours after treatment (Figure 3A). No considerable change was observed for total YAP protein levels in whole-cell lysates (Figure 3A), although this does not take into account the shift in nuclear and cytoplasmic localization seen in Figure 2F. To address potential off-target effects with LY, SJCRH30 and A204 cells were treated with a second pan–class I PI3K inhibitor, GDC-0941 (10 μM), which displayed similar results (Supplemental Figure 4, A and B). To test the hypothesis that PI3K inhibition results in LATS1/2-mediated phosphorylation of TAZ and YAP, we generated and expressed a LATS1/2-insensitive form of TAZ, Flag-TAZ4SA, in both SJCRH30 and A204 cells (Figure 3, B and C, and Supplemental Figure 4, C and D) (79). Treatment of LY in A204 Flag-TAZ cells exhibited an observable decrease in Flag-TAZ protein levels (Figure 3B). In contrast, LY did not diminish expression of Flag-TAZ4SA in A204 cells (Figure 3C), consistent with the hypothesis that PI3K-dependent regulation of TAZ is mediated via LATS1/2. Similar findings were observed in the SJCRH30 Flag-TAZ and Flag-TAZ4SA cell lines when treated with LY (Supplemental Figure 4, C and D). Finally, we sought to determine whether depletion of TAZ levels upon PI3K inhibition was due to proteasomal degradation secondary to phosphorylation by LATS1/2. A204 cells simultaneously treated with LY and 10 μM of MG-132 showed that MG-132 rescued proteasomal degradation of TAZ promoted by LY (Figure 3D). Similar results were found in the SJCRH30 cell line (Figure 3E). The above findings support a working model that inhibition of PI3K signaling in sarcoma cells results in LATS1/2-mediated phosphorylation of TAZ and YAP to promote their destabilization and proteasomal degradation (Figure 3F).

### Taz and Yap are central oncoproteins in a Trp53^fl/fl^Pten^fl/fl^ sarcoma mouse model.

**Figure 4:**
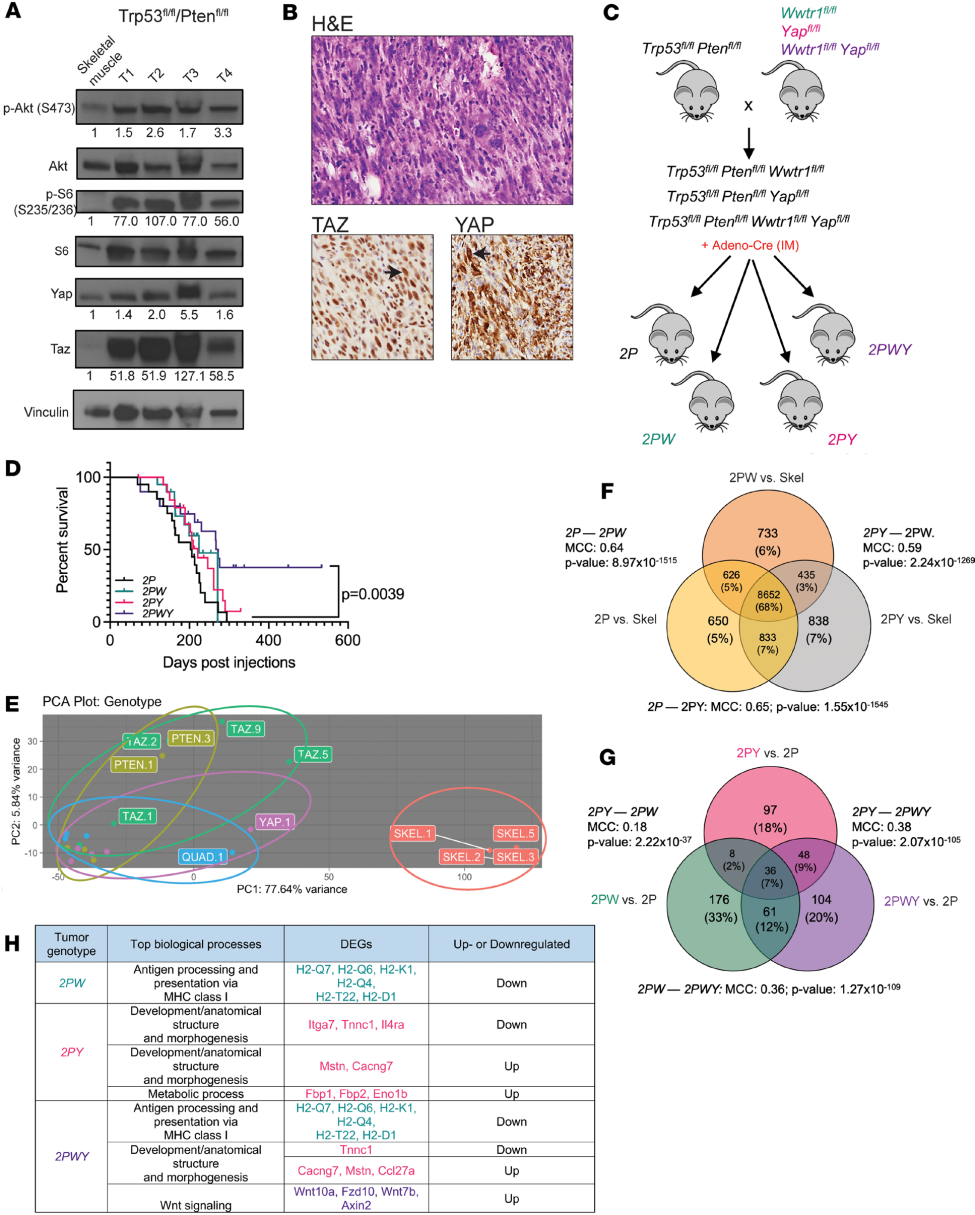
TAZ and YAP are critical oncoproteins in a PI3K-activated mouse model of sarcoma. (**A**) Western blot analysis of 4 representative tumors (T1, T2, T3, and T4) from *Trp53^fl/fl^Pten^fl/fl^* mice. (**B**) H&E section from a *Trp53^fl/fl^Pten^fl/fl^* primary tumor. IHC for TAZ and YAP. (**C**) Schematic for mouse experiment. (**D**) Survival curves for each mouse cohort. (**E**) Principal component analysis of RNA expression for 5 representative samples from murine skeletal muscle and *2P*, *2PW*, *2PY*, and *2PWY* tumors. (**F**) Number of differentially expressed genes in *2P*, *2PW*, and *2PY* tumors normalized to murine skeletal muscle. (**G**) Number of differentially expressed genes in *2PW*, *2PY*, and *2PWY* tumors normalized to *2P* tumors. (**H**) List of top biological processes and their respective differentially expressed genes (DEGs) for *2PW*, *2PY*, and *2PWY* tumors; although Wnt10a is differentially expressed in *2PW* mice, Wnt signaling as a biological process, overall, is not enriched. Statistical analysis for Kaplan-Meier survival analysis was performed with the log-rank (Mantel-Cox) test. The above experiment with *2P*, *2PW*, *2PY*, and *2PWY* mice was performed twice. To assess enrichment on RNA-Seq datasets, hypergeometric analyses and Matthews correlation coefficient calculations were carried out.

We previously showed that conditional KO of *Trp53* and *Pten* by adenovirus-mediated Cre recombinase (Ad CMV-Cre) expression injected intramuscularly resulted in the formation of predominantly undifferentiated pleomorphic sarcoma at the injection site (80). Tumor lysates in undifferentiated pleomorphic sarcomas derived from *Trp53^fl/fl^Pten^fl/fl^* mice showed activation of PI3K signaling, as demonstrated by elevated phospho-Akt (Ser473) and phospho-S6 (Ser235/236) relative to skeletal muscle controls (Figure 4A). Consistent with the above in vitro studies, total TAZ and YAP in these tumor lysates were elevated relative to skeletal muscle controls. To determine whether TAZ and YAP were activated in these tumors, we performed IHC on tumors from *Trp53^fl/fl^Pten^fl/fl^* mice. In agreement with the above findings by Western blot, IHC for TAZ and YAP demonstrated diffuse and strong expression and nuclear localization of the 2 transcriptional coactivators in essentially all of the tumors (Figure 4B).

To determine whether TAZ and YAP were functionally important to the formation of PI3K-driven sarcomas in vivo*,* *Trp53^fl/fl^Pten^fl/fl^* mice (hereafter referred to as *2P*) were crossed with *Wwtr1^fl/fl^* mice to generate *Trp53^fl/fl^Pten^fl/fl^Wwtr1^fl/fl^* mice (*2PW)*, *Yap1^fl/fl^* mice to generate *Trp53^fl/fl^Pten^fl/fl^Yap1^fl/fl^ mice* (*2PY*), and *Wwtr1^fl/fl^Yap1^fl/fl^* mice to generate *Trp53^fl/fl^Pten^fl/fl^Wwtr1^fl/fl^Yap1^fl/fl^* mice (*2PWY*) (Figure 4C). Loss of expression of Taz, Yap, or both in the above arms of the study was confirmed by IHC (Supplemental Figure 5A). Mice (*n* = 20) in each arm of the study were injected intramuscularly with Ad CMV-Cre recombinase with Ad CMV-eGFP controls as described in the Methods. Subsequent to intramuscular delivery of Ad CMV-Cre, survival analysis for each cohort was performed once tumors reached 1.5 cm in the greatest dimension at the site of injection (according to animal use protocol; see Methods for details). No differences in the histological types of sarcomas generated were seen between the different groups, which were composed predominantly of undifferentiated pleomorphic sarcoma/undifferentiated spindle cell sarcoma as previously described in the *Trp53^fl/fl^Pten^fl/fl^* mouse model (80) (see Supplemental Table 1). Survival analysis showed that relative to *2P* mice, conditional KO of either *Wwtr1^fl/fl^* (*2PW*, *P* = 0.17) or *Yap1* (*2PY*, *P* = 0.17) alone did not improve overall survival, whereas loss of both significantly extended the overall survival (*2PWY*, *P* = 0.0039) (Figure 4D). This indicates that TAZ and YAP have distinct complementary functions in this PI3K-activated, sarcoma-prone mouse model.

To determine whether differences in survival were due to differences in time to tumor formation, we compared the time to tumor formation among the 4 phenotypes and saw that 2*PY* mice showed an increased latency to tumor formation compared with *2P* mice (176 days vs. 126 days; *P* = 0.007) (Supplemental Figure 5, B, and D–G). *2PWY* mice showed a trend toward increase in the latency of tumor formation; however, this did not reach statistical significance (*P* = 0.11). Finally, we compared the fraction of mice that developed tumors in the span of the experiment, 533 days, and found that although tumors developed in all mice of the *2P*, *2PY*, and *2PW* genotypes, 6 of the 20 mice (30%) with the *2PWY* genotype did not develop tumors at the injection site by the end of the study. To determine whether there were differences in the growth rate of the tumors, we evaluated the time to tripling of the volume of the tumors, and found that tumors from the *2PW*, *2PY*, and *2PWY* genotypes showed no difference compared with the *2P* controls (Supplemental Figure 5, C, and D–G). We have repeated this experiment with similar findings. Taken together, the data suggest that TAZ and YAP, when coordinately inactivated in vivo, may play a role in tumor initiation.

To determine whether the above phenotype in vivo seen with inactivation of TAZ and YAP was due to changes in transcription, RNA was isolated from 5 tumors (undifferentiated pleomorphic sarcoma) from each genotype, and total RNA-Seq was performed. By principal component analysis, samples from the groups tended to cluster together and were distinct relative to normal skeletal muscle tissue (Figure 4E and Supplemental Figure 6, A–D). Beginning with *2P* (designated PTEN), ellipses representing tumor samples from each arm of the study demonstrated a clockwise rotation corresponding to sequential loss of Taz (*2PW*), Yap (*2PY*), or both (*2PWY;* designated QUAD). (Figure 4E). This arrangement suggests a step-wise change in the overall transcriptome with inactivation of TAZ, YAP, or both. The transcriptomes of 2PW and 2PY overlapped significantly with 2P (Matthews correlation coefficient of 0.64 and 0.65, respectively, *P* < 0.0001 for both comparisons) (Figure 4F). These findings are consistent with the working model that TAZ and YAP are downstream transcriptional effectors of PI3K signaling.

Differentially expressed genes (DEGs) in the conditional KO of both *Wwtr1* and *Yap1* (*2PWY*) overlapped with DEGs seen in the *2PW* and *2PY* transcriptomes. However, conditional KO of Taz and Yap in *2PWY* mice did not simply have an additive effect on gene expression; the *2PWY* transcriptome was also composed of a subset of genes that was unique and likely represented a set of genes coordinately regulated by both Taz and Yap (Figure 4G). By pathway analysis, conditional KO of *Wwtr1* (*2PW*) showed inactivation of genes important for antigen processing and presentation and Wnt signaling (Figure 4H). Conditional KO of *Yap1* (*2PY*) showed differential expression of a series of genes important for development (Figure 4H). The *2PWY* transcriptome demonstrated DEGs identified by pathway analysis in the *2PW* and *2PY* transcriptomes. However, consistent with the Venn diagram analysis showing DEGs unique to the *2PWY* transcriptome (Figure 4G), pathway analysis also showed differential expression of genes involved in Hippo signaling (*Wnt10a*, *Fzd10*, *Wnt7b*, *Axin2*) and mTOR biological process (*Wnt10a*, *Fzd10*, and *Wnt7b*) unique to the *2PWY* transcriptome (Figure 4H). Well-defined transcriptional targets of Taz and Yap, *Ccn1* (Cyr61) and *Ccn2* (Ctgf), were downregulated in *2PWY* mice versus *2P* mice (log_2_ fold-change of –0.193 for *Ccn2* and –0.059 for *Ccn1*), although this did not reach statistical significance (Supplemental Table 2). Collectively, the above in vivo data suggest that TAZ and YAP are key oncogenic transcriptional effectors in a PI3K-activated mouse model of sarcoma.

### mTORC1 inhibition has a limited therapeutic effect in vitro and in vivo.

**Figure 5:**
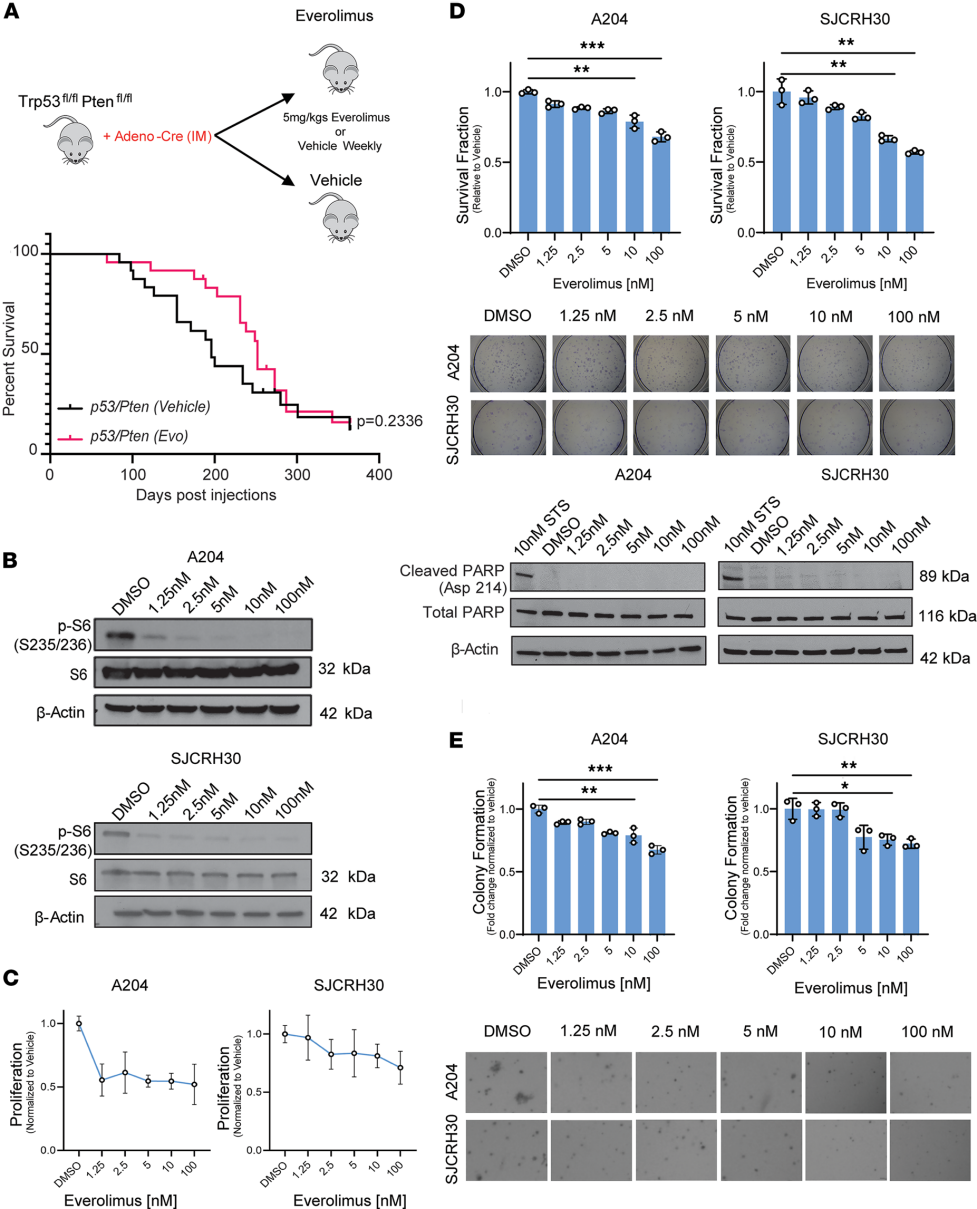
mTORC1 inhibition shows a modest effect on proliferation and growth in vivo and in vitro. (**A**) Everolimus (5 mg/kg) or vehicle (DMSO) was given weekly in *2P* mice. Kaplan-Meier survival analysis shown. (**B**) Western blot evaluating p-S6 (S235/236) as a function of everolimus concentration. (**C**) Drug response curve after treatment with everolimus for 72 hours. (**D**) Clonogenic assay and evaluation of PARP-cleavage after treatment with everolimus for 72 hours. (**E**) Soft agar assay after treatment with everolimus. For soft agar and clonogenic assays, statistical significance was evaluated using an unpaired 2-tailed *t* test. All in vitro assays were repeated at least twice. Statistical analysis for Kaplan-Meier survival analysis was performed with the log-rank (Mantel-Cox) test. *P < 0.05, **P < 0.01, ***P < 0.001.

Taken together, the above in vitro and in vivo data suggest that the PI3K/TAZ/YAP axis represents another key signaling axis in addition to the PI3K/AKT/mTORC1 axis. To begin assessing the relative efficacy of targeting the 2 axes, we evaluated the efficacy of targeting the PI3K/AKT/mTORC1 axis utilizing everolimus (a rapamycin derivative) in vivo in the PI3K-driven *Trp53^fl/fl^ Pten^fl/fl^* sarcoma mouse model mentioned above (Figure 4). Everolimus (5 mg/kg) or vehicle (DMSO) was administered orally on a weekly basis as previously described (81). Tumor dimensions were measured until an endpoint of 2 cm was met. Kaplan-Meier analysis indicated an initial survival benefit to single-agent therapy with everolimus: the maximum difference between the survival curves seen at 220 days showed a survival fraction of 0.79 for everolimus, whereas vehicle treatment had a survival fraction of 0.44. However, this difference in survival decreased over the course of the experiment, with no change in overall survival at the culmination of the study (*P* = 0.2336) (Figure 5A). This is consistent with clinical data showing that mTORC1 inhibition alone is not effective in reducing tumor burden/survival in vivo as has been documented in various contexts (82–84).

To dissect the therapeutic limitations of everolimus observed in vivo, we treated the SJRCH30 and A204 cell lines with everolimus and assessed various hallmarks of cancer in vitro. Everolimus was shown to decrease phosphorylation of the S6 ribosomal subunit at a wide range of concentrations spanning from 1 nM to 100 nM (10 nM is a concentration often utilized clinically) (85) (Figure 5B). Reduction in S6 phosphorylation was observed at sub-10 nM concentrations of everolimus in both the SJCRH30 and A204 cell lines (Figure 5B). In contrast, this inhibitory effect resulted in only a modest decrease in proliferation, even at higher concentrations of 100 nM (10-fold higher than the clinically relevant dose) (Figure 5C). To determine whether this decrease in proliferation could be due to cytotoxic effects of everolimus, we performed clonogenic outgrowth assays over a range of concentrations. Clonogenic assays showed survival fractions that were marginally reduced at physiologically relevant doses of everolimus and only reduced by 25%–40% at the highest concentration of 100 nM (Figure 5D). No change in cleaved PARP was seen via Western blot over this range of concentrations of everolimus. Taken together, the above findings demonstrate low cytotoxicity and an absence of apoptosis with treatment with everolimus. Similarly, effects of mTORC1 inhibition on anchorage-independent growth were modest and not seen until higher concentrations (Figure 5E).

### TEAD inhibition alone has modest efficacy in vitro and predominantly affects anchorage-independent growth.

**Figure 6:**
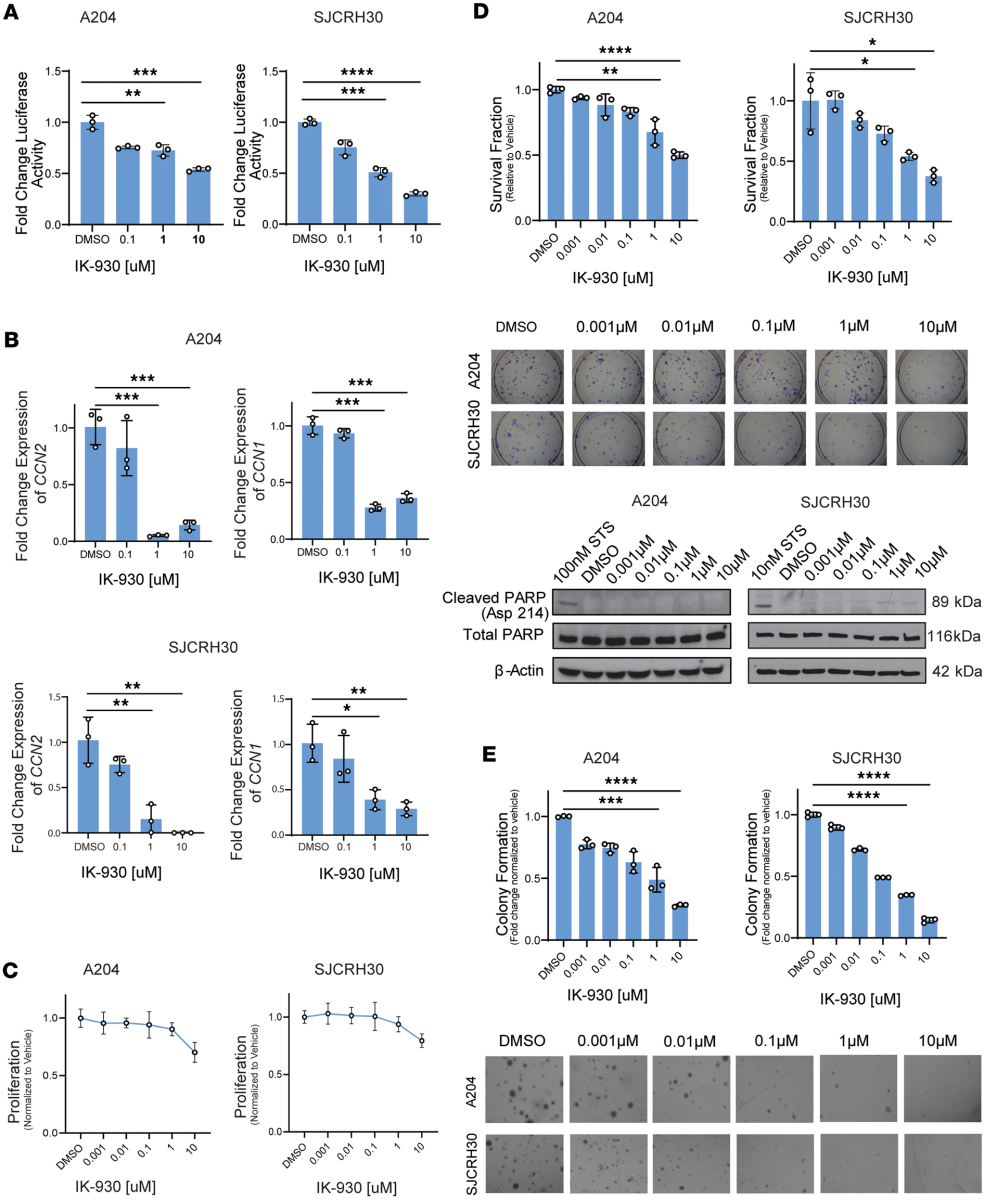
YAP/TAZ inhibition markedly reduces growth-related phenotype. (**A**) Dual luciferase reporter assay after treatment with IK-930. (**B**) Quantitative RT-PCR (qRT-PCR) after treatment with IK-930. (**C**) Drug response curve after treatment with IK-930 for 72 hours. (**D**) Clonogenic assay and evaluation of PARP-cleavage after treatment with IK-930 for 72 hours. (**E**) Soft agar assay after treatment with IK-930. For quantitative RT-PCR and luciferase reporter assays, SD was calculated from fold-change values for each triplicate. For all assays, statistical significance was evaluated using an unpaired 2-tailed *t* test. All in vitro assays were repeated at least twice. *P < 0.05, **P < 0.01, ***P < 0.001, ****P < 0.0001.

We then assessed whether it may be more efficacious to target the PI3K/TAZ/YAP axis. To determine whether IK-930, a recently described TEAD inhibitor (86), disrupts TEAD-dependent transcription, A204 and SJCRH30 cell lines were transduced with a TEAD luciferase (pLV-8XTEADrep) reporter construct plasmid. Luciferase activity was measured in the SJCRH30 and A204 cell lines after 72 hours of treatment with various concentrations of IK-930 (Figure 6A). A dose-dependent reduction in TEAD reporter activity was seen in both A204 and SJCRH30 cell lines with IK-930 treatment (Figure 6A).

To confirm that IK-930 disrupts expression of TAZ/YAP-TEAD transcriptional targets, A204 and SJCRH30 cell lines were treated with 1 μM IK-930 for 24 hours. To evaluate the effect of TEAD inhibition on well-defined transcriptional targets of YAP and TAZ, qRT-PCR was done using the clinically relevant concentration of 1 μM IK-930 in the SJCRH30 and A204 cell lines (Figure 6B). Expression of *CCN2* (CTGF) and *CCN1* (CYR61) was decreased approximately 5-fold upon treatment with 1 μM, a clinically relevant dose, of IK-930 in both cell lines (Figure 6B).

To examine the effect of IK-930 on sarcoma cell proliferation, we treated SJCRH30 and A204 cells with various concentrations of IK-930, which resulted in modest reductions in proliferation; only the higher concentrations of IK-930 (1 μM, 10 μM) showed a slight effect (Figure 6C). Similar effects were observed when performing a clonogenic assay for IK-930, with no marked effects on the survival fraction until concentrations reached higher levels between 1 μM and 10 μM (Figure 6D). To investigate the effect of TEAD inhibition on apoptosis, Western blot was performed on A204 and SJCRH30 cells to examine PARP cleavage (Figure 6D). No increase in cleaved PARP was identified with increasing concentrations of IK-930, suggesting the limited cytotoxicity seen in higher concentrations of IK-930 may be due to other mechanisms of cell death (e.g., necrosis, ferroptosis) (Figure 6D). YAP and TAZ have been implicated in anchorage-independent growth in various cancer types (87–91). To test the effect of TEAD inhibition on anchorage-independent growth, we performed soft agar assays for both cell lines (Figure 6E). In contrast to proliferation, IK-930 demonstrated reduction in anchorage-independent growth at lower concentrations, including the 1–10 nM range (Figure 6E). Taken together, TEAD inhibition had the greatest effect on anchorage-independent growth, with more modest effects on proliferation and cytotoxicity.

### mTORC1 and TEAD inhibitors show synergistic activity in reducing proliferation and soft agar growth.

**Figure 7:**
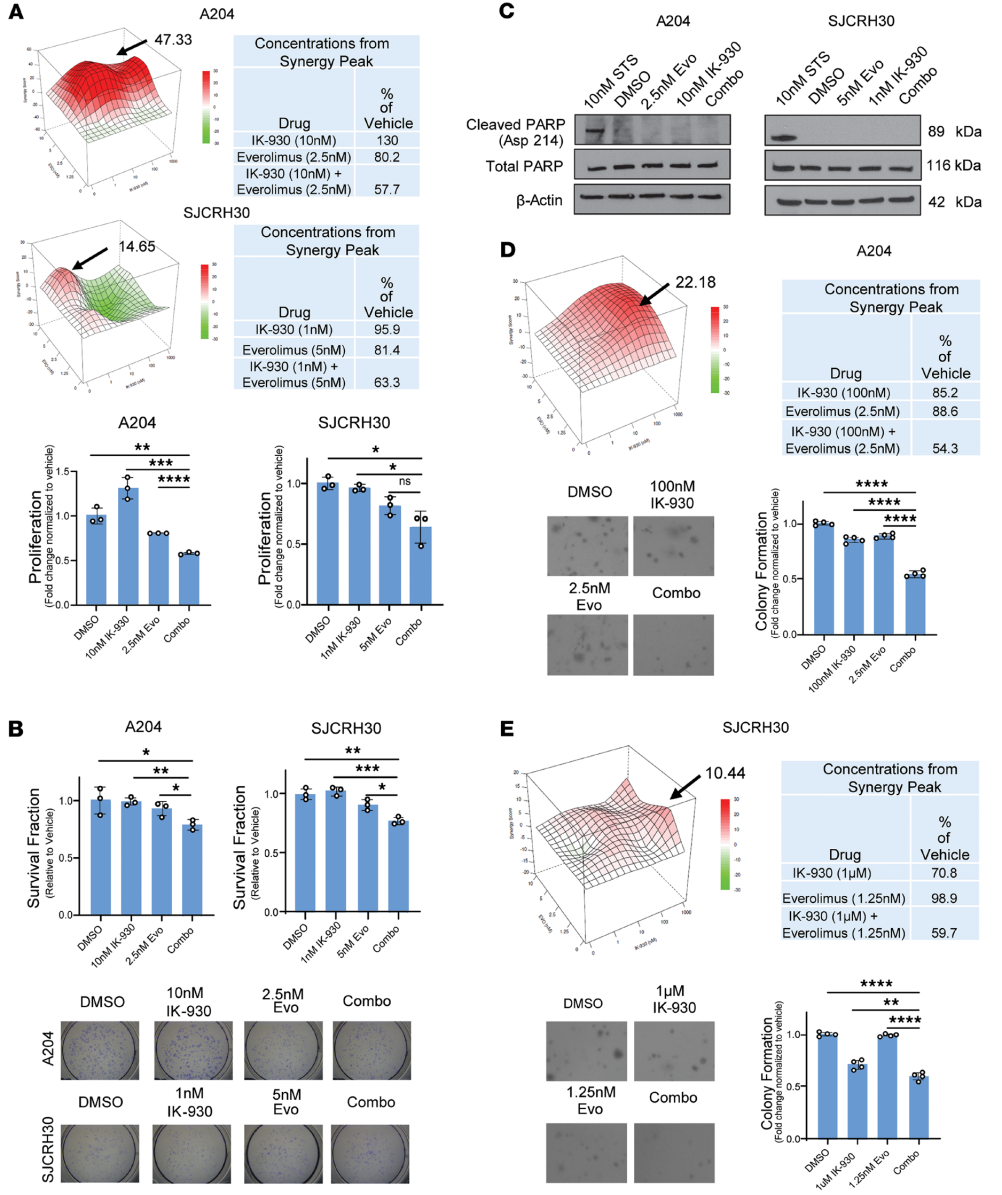
Pharmacological inhibition of YAP/TAZ and mTORC1 works synergistically in vitro. (**A**) Cells treated with combinational drug matrix of IK-930 and everolimus at indicated doses for 72 hours and cell viability evaluated by MTT-style assay. Arrows demonstrate concentrations of IK-930 and everolimus further evaluated. (**B**) Clonogenic assay using the synergistic concentrations in **A** showed statistically significant reduction in clonogenic outgrowth. (**C**) Western blot evaluating cleaved PARP after monotherapy and combination therapy for the synergistic concentration in **B**. (**D** and **E**) Synergy scores and 3D surface plots of combination studies in soft agar in the A204 cell line (**D**) and SJCRH30 cell line (**E**). Arrows demonstrate concentrations of IK-930 and everolimus further evaluated in table form. All in vitro assays were repeated at least twice. Synergy scores and 3D surface plots of cell viability and soft agar growth were quantified and analyzed with the Bliss model using SynergyFinder. Staurosporine was used as a positive control. For soft agar assay, statistical significance was evaluated using an unpaired 2-tailed *t* test. *P < 0.05, **P < 0.01, ***P < 0.001, ****P < 0.0001.

The above data showed that inhibition of mTORC1 (everolimus) predominantly affected proliferation, whereas inhibition of TAZ/YAP (with TEAD inhibitor IK-930) predominantly affected anchorage-independent growth; both drugs exhibited limited cytotoxic effect. This led to the hypothesis that targeting both the PI3K/AKT/mTORC1 and PI3K/TAZ/YAP axes might be more effective from a therapeutic standpoint. To test this hypothesis, we treated the SJCRH30 and A204 cell lines with varying concentrations of everolimus and IK-930 in combination. A range of concentrations centered around their IC_50_ were used and the online tool SynergyFinder (92) was used to identify synergistic concentrations of the 2 drugs. The Bliss quantitative model was used (score greater than 10 is synergistic) (93) with the Loewe synergy score (score greater than 0) (94) calculated as a confirmatory method (92, 95). Using the Bliss quantitative model within the SynergyFinder algorithm, we found that the effect of combination therapy on proliferation was greater than that of either single agent (Figure 7A). The Bliss synergy score was higher for A204 (47.3) as compared with the SJCRH30 cell line (14.7), but the contours of the synergy maps showed similar patterns, with a primary peak at lower concentrations of IK-930 (≤10 nM) and everolimus (≤5 nM) (Figure 7A). The Loewe synergy score for A204 and the SJCRH30 cells was 17.1 and 3.6, respectively. For A204, 10 nM of IK-930 and 2.5 nM of everolimus showed a synergistic reduction in proliferation by approximately 43% in comparison with vehicle. Whereas for SJCRH30, 1 nM of IK-930 and 5 nM of everolimus showed a synergistic reduction in proliferation by 37% in comparison with vehicle. In contrast, at the above concentrations, single-agent IK-930 showed a 0%–4% reduction in proliferation, while single-agent everolimus demonstrated a 20% reduction in proliferation.

These synergistic concentrations in Figure 7A were then used to study the effect on clonogenic outgrowth in SJCRH30 and A204 cell lines (Figure 7B). Statistically significant reductions in clonogenic outgrowth compared with vehicle control, 23% for SJCRH30 (*P* = 0.043) and 21% for A204 (*P* = 0.0027), were seen using these low, physiologically relevant concentrations. To determine whether this effect on clonogenic outgrowth was due to apoptosis, Western blot analysis was used to evaluate changes in cleaved PARP. PARP cleavage was not identified with the combination of everolimus and IK-930 or the single-agent controls (Figure 7C). Taken together, the above data suggest the combination therapy does cause some cytotoxicity in an apoptosis-independent manner (96, 97) at lower, more clinically relevant concentrations than everolimus alone (Figure 5D) or IK-930 alone (Figure 6D). To investigate the effect of combination therapy on anchorage-independent growth, soft agar assays were performed utilizing varying concentrations of both IK-930 and everolimus in agarose. Synergistic activity of TEAD inhibition and mTORC1 inhibition at 100 nM and 2.5 nM, respectively, was seen, represented by a peak with a Bliss synergy score of 22.2 in the A204 cell line (Figure 7D). The Loewe synergy score was 17.3. The combination therapy reduced anchorage-independent growth by 46%, while single-agent therapy with everolimus or IK-930 at those concentrations only reduced anchorage-independent growth by approximately 15% in the A204 cell line (Figure 7D). For SJCRH30, the synergy peak was seen at 1 μM of IK-930 and 1.25 nM of everolimus with a Bliss synergy score of 10.4 (Figure 7E). The Loewe synergy score was 7.5. The combination therapy reduced anchorage-independent growth by 40%, while single-agent IK-930 and everolimus reduced anchorage-independent growth by around 30% and 2%, respectively, in the SJCRH30 cell line (Figure 7E).

Everolimus alone predominantly affected proliferation (Figure 5), and TEAD inhibition alone predominantly affected anchorage-independent growth (Figure 6), and the above data show that the combination therapy synergistically affected proliferation (Figure 7A) and anchorage-independent growth (Figure 7D), while causing some apoptosis-independent cell death at more physiologically relevant doses than the single-agent approaches (Figure 7B).

### Combination therapy with TEAD and mTORC1 inhibitors reduces tumor volume in vivo.

**Figure 8:**
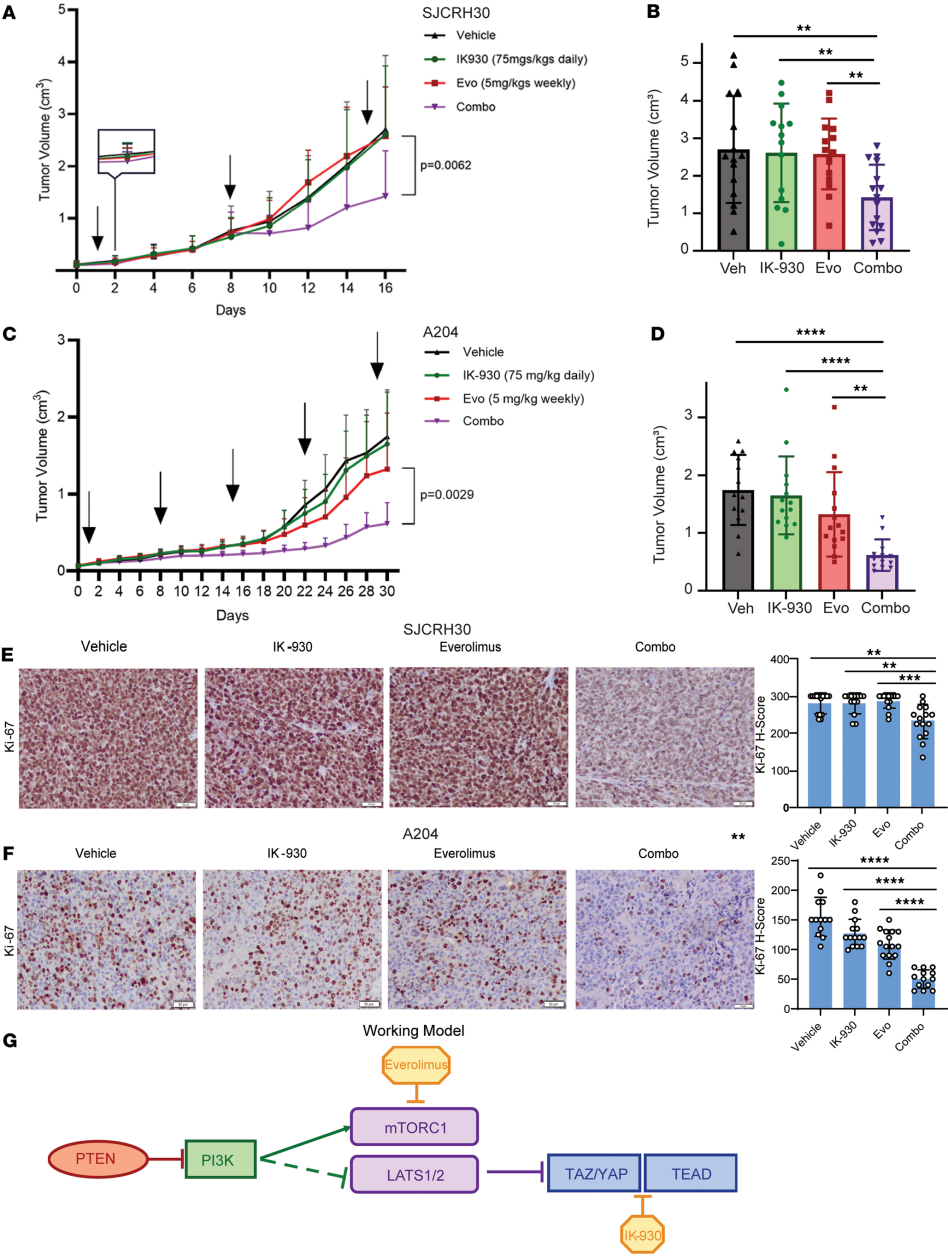
Combination therapy targeting YAP/TAZ and mTORC1 inhibits tumor growth in vivo. (**A**) Tumor growth curve for SJCRH30 xenograft in NSG mice receiving IK-930, everolimus (Evo), combination IK-930 and everolimus (Combo), and DMSO (Vehicle). Arrows denote weekly administration of everolimus. (**B**) Tumor volumes at the end of study in **A**. (**C**) Tumor growth curve for A204 xenograft in NSG mice receiving IK-930 (75 mg/kg daily), everolimus (Evo) (5 mg/kg weekly), combination IK-930 and everolimus (Combo), and DMSO (Vehicle). Arrows denote weekly administration of everolimus. (**D**) Tumor volumes at the end of study in **C**. (**E**) IHC for Ki-67 on SJCRH30 tumors. (**F**) IHC for Ki-67 on A204 tumors. (**G**) Simplified working model. For photomicrographs, scale bar: 50 μm (200×). For tumor volume and histological analysis, statistical significance was evaluated using an unpaired 2-tailed *t* test. **P < 0.01, ***P < 0.001, ****P < 0.0001.

To determine whether this combination therapy would be effective in vivo, we further evaluated the effect of combination therapy in a mouse xenograft model. First, 5 × 10^6^ SJCRH30 cells were subcutaneously injected in *Prkdc^scid^Il2rg^tm1Wjl^/SzJ* (NSG) mice. After tumors were palpable (5 mm^3^), mice were divided into 4 groups: vehicle, IK-930 alone, everolimus alone, and IK-930 combined with everolimus. IK-930 (75 mg/kg) was administered orally every day; everolimus (5 mg/kg) was administered orally on a weekly basis (81). Tumor volume was measured every other day until tumors in the vehicle arm averaged 2 cm. Of note, in the combination therapy arm, every treatment of everolimus (weekly administration of everolimus denoted by arrows) resulted in a reduction of tumor growth (Figure 8A). At the end of the study, tumor volume was measured in situ in the mice, showing an almost 2-fold reduction in tumor volume for combination therapy–treated mice in comparison to vehicle and single-agent therapy controls (*P* < 0.01) (Figure 8B). This experiment was repeated with the A204 cell line (Figure 8C). Similar to the SJCRH30 cell line xenograft model, combination therapy with IK-930 and everolimus resulted in a greater reduction of tumor growth in the A204 cell line xenograft model than each of the single-agent controls or vehicle controls (Figure 8C). Similar to the SJCRH30 cell line xenograft model, tumor volume in the combination therapy was reduced approximately 2-fold or greater when compared with the single-agent arms of the study and vehicle control (Figure 8D). Histological evaluation of resected tumors from both xenograft models showed no change in the percentage of necrosis between the different treatment arms (Supplemental Figure 7, A and C). Similarly, IHC showed no change in cleaved caspase-3 activity between the different treatment arms (Supplemental Figure 7, B and D). However, in the SJCRH30 xenograft model, an approximately 20% reduction in Ki-67, a proliferation marker, was seen in the combination therapy cohort in comparison with vehicle-treated mice (*P* = 0.001) and single-agent–treated mice (*P* = 0.0002 for everolimus and *P* = 0.002 for IK-930) (Figure 8E). In the A204 xenograft model, a similar trend was observed, with an approximately 50% reduction in Ki-67 in the combination therapy as compared with the closest control arm, the everolimus-alone arm (*P* < 0.0001) (Figure 8F). No significant differences were identified in mouse weight after treatment for the combination therapy arm in both the SJCRH30 and A204 xenograft models (Supplemental Figure 7, E and F), indicating the treatment regimen was tolerated by the mice. Collectively, these results support the working model that targeting both the PI3K/AKT/mTORC1 and PI3K/TAZ/YAP axes can be leveraged to more completely inhibit oncogenic PI3K signaling in sarcomas and potentially other cancers (Figure 8G).

## Discussion

Overall, sarcomas are a difficult to treat group of cancers. Surgical resection is a mainstay in the treatment of sarcomas, but radiation therapy and chemotherapy can be given in the neoadjuvant and adjuvant setting for certain subsets of sarcomas. Chemotherapy (usually anthracycline-based) can be administered to treat metastatic sarcomas (98). Localized sarcomas have a 5-year overall survival of approximately 81%, sarcomas with regional spread have a 5-year survival rate of 56%, and metastatic sarcomas have a 5-year survival rate of 15% (99). Additional therapeutic targets for sarcomas are needed.

Our studies show that despite a low mutational burden for some of the core PI3K signaling enzymes, PI3K signaling could be activated at some level in up to 60% of sarcomas due to absent or very low PTEN expression, representing an underutilized, common therapeutic target active in various sarcomas. Loss of expression of PTEN varied across different histological types but is prevalent in key sarcoma histological types, including undifferentiated pleomorphic sarcoma/undifferentiated spindle cell sarcoma, the most common histological type of sarcoma (1). This observation is validated by the finding that the *Trp53^fl/fl^Pten^fl/fl^* sarcoma mouse model generates predominantly undifferentiated pleomorphic sarcomas (80). The loss of PTEN expression seen at the protein level in clinical samples (30%–60%) (Figure 1, C and D) exceeds the frequency of mutations or deletions seen in *PTEN* at the genomic level (5%–10%) (Figure 1B), suggesting that the epigenetic mechanisms may be playing a role in the regulation of PTEN. Previous reports have shown that YAP suppresses PTEN expression by inducing expression of miR-29 in epithelial cell systems and carcinomas (30). Additional studies are needed to elucidate the mechanisms leading to the loss of expression of PTEN in sarcomas.

One persistent question in the study of PI3K signaling is how signals from the cell surface are transduced into the nucleus. Unlike other oncogenic signal transduction pathways, a bona fide oncogenic transcription factor downstream of PI3K has not been elucidated. In these studies, we found that TAZ and YAP represent an additional arm of the PI3K signaling axis and represent oncogenic transcription factors/transcriptional coactivators identified downstream of PI3K signaling. Our observations support a working model that a PI3K/TAZ/YAP axis exists in parallel to a PI3K/AKT/mTORC1 axis in sarcomas and potentially other cancers. Our work shows that PI3K regulates TAZ/YAP via a LATS1/2-dependent mechanism in sarcomas, which may differ from mechanisms implicating PDK1, AKT1, or GSK-3β derived from studies in epithelial cells (33, 35, 75, 76). In gastric carcinomas, phosphorylated (inactivated) PTEN disrupted the MOB1-LATS1/2 interaction, resulting in YAP nuclear localization and activation (100). Future studies are warranted to identify the intermediate steps by which PI3K signaling inactivates LATS1/2 to activate TAZ/YAP in sarcomas.

TAZ and YAP are well known drivers of initiation, progression, and metastasis (101). However, to our knowledge, analysis of the role and relative contributions of Taz and Yap in genetically engineered mouse models of sarcomas have not been explored. It is increasingly appreciated that TAZ and YAP do not completely phenocopy each other and may functionally compensate for one another (102, 103). We showed in a PI3K-driven sarcoma mouse model that inactivation of both Taz and Yap are needed to inhibit tumor initiation (in approximately 30% of animals) and have an effect on overall survival (Figure 4D). This finding suggests that TAZ and YAP have complementary roles in PI3K-driven sarcomagenesis and is consistent with the transcriptomic data shown above, which showed that TAZ and YAP have overlapping yet distinct transcriptomes and coordinately regulate a unique set of genes in the PI3K transcriptome (Figure 4, F and G). Collectively, our findings and those of others indicate that Taz and Yap play distinct and complementary roles in sarcoma development and mediate the oncogenic PI3K transcriptome. Future studies are needed to further elucidate how the PI3K/TAZ/YAP transcriptome drives sarcoma formation.

PI3K signaling is activated in a number of cancers, including breast and ovarian cancer due to mutations in *PIK3CA* and *AKT1/2/3*, and loss of *PTEN* (104–107). Several approaches have been used to target these cancers driven by PI3K signaling including targeting of PI3K, inhibition of AKT, combination approaches targeting PI3K and AKT, inhibition of mTORC1 (108, 109), and combination approaches targeting PI3K and mTORC1 (110). Most current approaches targeting PI3K signaling have utilized rapamycin derivatives (e.g., sirolimus, everolimus), while acknowledging limitations due to various feedback mechanisms (6, 26, 111–113). To address these limitations, mTORC1 inhibition has been pursued in sarcomas for clinical trials in combination with other therapeutic agents (114, 115). Malignant perivascular epithelioid cell tumor (PEComa) is currently the only sarcoma in which mTORC1 inhibition is approved (116). Our studies largely recapitulate these observations (Figure 5). Although everolimus showed a modest decrease in proliferation, there was limited cytotoxicity in vitro. Initial gains in overall survival in vivo diminished over time as tumors became resistant to rapamycin-based therapy. Taken together, our findings and previous studies demonstrate limitations in treating PI3K-driven cancers including sarcomas with mTORC1 inhibition alone. Additional therapeutic approaches are needed to target PI3K signaling in these cancers, including targeting of the PI3K/TAZ/YAP axis.

The above findings show that YAP/TAZ are downstream oncogenic transcriptional coactivators of PI3K signaling, thus providing a rationale for combination therapy targeting the TEAD transcription factors along with mTORC1 inhibitors. We show that IK-930 as a single agent was effective in decreasing anchorage-independent growth and that everolimus inhibited proliferation in vitro, but they were both ineffective as single-agent therapies in vivo. Consistent with our in vitro studies, combining the 2 drugs in vivo showed a synergistic decrease in proliferative index and tumor volume that could have relevance to the treatment of sarcomas and potentially other PI3K-dependent cancers. The synergistic effect, both in vitro and in vivo, between YAP/TAZ-TEAD and mTORC1 inhibition can potentially be explained by the hypothesis that this approach inhibits both the transcriptional (YAP/TAZ) and translational (mTORC1) apparatus downstream of PI3K signaling (87, 117, 118).

Although TEAD inhibitors may be effective as a single agent in some settings, concerns regarding their efficacy as a monotherapy exist. Indeed, our studies show that as a single agent, a TEAD inhibitor was insufficient to inhibit sarcoma growth in a TAZ/YAP-dependent xenograft mouse model. It was only with combination targeting of a parallel signaling axis that the TEAD inhibitor was effective. Rational combination of a TEAD inhibitor with additional agents targeting a relevant pathway is emerging as a viable therapeutic strategy (119–123). Combination therapy with TEAD inhibitors may also be a reasonable approach to mitigating the risk of secondary resistance developing to TEAD inhibitors. For these reasons, additional investigation into the basic mechanisms driving or complementing TAZ and YAP activation in different cancers is needed to more effectively utilize TEAD inhibitors in TAZ/YAP-dependent cancers.

## Methods

### Sex as a biological variable.

Sex was considered as a biological variable and approximately equal numbers of male and female animals were utilized for our mouse studies.

### Statistics.

For soft agar and clonogenic assays, statistical significance was evaluated using Student’s unpaired 2-tailed *t* test. For Kaplan-Meier curves, significance was determined by log-rank (Mantel-Cox) test. For tumor initiation, volume tripling, and weight, statistical significance was determined using an unpaired 2-tailed *t* test with Welch’s correction to account for different sample sizes between groups. For immunofluorescence assays, statistical significance was evaluated using Student’s unpaired 2-tailed *t* test. To assess enrichment on RNA-Seq datasets, hypergeometric analyses and the Matthews correlation coefficient calculations were carried out in R, version 4.4.1. The upper tail of the hypergeometric distribution was calculated using the phyper() function. Each experiment was repeated at least twice. Data are shown as mean ± SD. *P* values of less than 0.05 were considered significant.

### Study approval.

Mouse experiments were approved by the IACUC (protocol 2052228) at the University of Iowa. Sarcoma samples were retrieved from the University of Iowa Department of Pathology with previous approval from the IRB (ID 201408808) at the University of Iowa.

### Data availability.

The RNA-Seq data reported in this paper for mouse tumors have been deposited in NCBI’s Gene Expression Omnibus (GEO GSE274982). Full access to Supporting data values in the manuscript is provided in the Supporting Data Values file.

See Supplemental Methods for additional methodology.

## Author contributions

MRT was responsible for conceptualization. MSC, PJB, and MRT contributed to methodology. MSC and PJB were responsible for software. MSC, PJB, and MRT performed the formal analysis. KCG, AAK, KG, SS, NS, GD, CF, NM, YD, SYY, ML, PJB, MSC, and MRT conducted the investigation. MDH, PJB, and MRT provided resources. KCG, AAK, and MRT wrote the original draft. KCG, AAK, and MRT reviewed and edited the manuscript. MRT supervised the study. KCG, AAK, and MRT acquired funding.

## Funding support

This work is the result of NIH funding, in whole or in part, and is subject to the NIH Public Access Policy. Through acceptance of this federal funding, the NIH has been given a right to make the work publicly available in PubMed Central.

- Veterans Health Administration Merit Review Program (1 I01 BX003644-01, to MRT).
- NIH, National Cancer Institute (NCI) (1 R01 CA237031-01A1, to MRT).
- NIH, NCI (1 R01 CA237031-01A1S1, to KCG and MRT).
- University of Iowa Stead Family Scholars Program (to MRT).
- University of Iowa Sarcoma Multidisciplinary Oncology Group pilot award (to MRT).
- Center for Biocatalysis and Bioprocessing Predoctoral Fellowship (to AAK).
- NCI Core grant P30 CA086862 (University of Iowa Holden Comprehensive Cancer Center).

## Supplementary Material

Supplemental data

Unedited blot and gel images

Supplemental table 2

Supporting data values

## Version 1 02/12/2026

In-Press Preview

## Version 2 03/23/2026

Electronic publication

## Version 3 03/25/2026

Affiliation 9 added
