# Supplementary material for: PI3K regulates TAZ/YAP and mTORC1 axes that can be synergistically targeted
Source: JCI Insight. 2026 Feb 10;11(6):e191600. doi: 10.1172/jci.insight.191600 (PMC13043097; doi:10.1172/jci.insight.191600)
Supplement: Supplemental data [file jciinsight-11-191600-s373.pdf]

Supplemental Methods

Cell culture

Cell lines were obtained from American Type Culture Collection and grown in the recommended growth culture medium. SJCRH30 (human alveolar rhabdomyosarcoma) and A204 (human malignant extrarenal rhabdoid tumor) cells were obtained from American Type Culture Collection (ATCC, Manassas, VA, USA). SJCRH30 and A204 cells were cultured in RPMI and McCoy's 5A media (Gibco), respectively. Media was supplemented with (according to ATCC recommendations) 10% fetal bovine serum (Invitrogen-Life Technologies), 1 mM sodium pyruvate, and 50 μg/mL penicillin/streptomycin. All cells were cultured at 37 °C and 5% CO_2. For PI3K inhibition studies, SJCRH30 and A204 cells were treated with 30 μM/60 μM LY294002 (TOCRIS, catalog# 1130), 10 μM GDC-0941 (Selleckchem, catalog# S1065). SJCRH30 and A204 cells were treated with 10 nM-10 μM of the AKT inhibitor MK-2206 2HCl (Selleckchem, catalog# S1078). To evaluate proteasomal degradation, SJCRH30 and A204 cells were treated with 10 μM of MG-132 (Sigma-Aldrich, catalog# C2211) alone or in combination with 60 μM LY294002. To evaluate PARP cleavage by western blot, Staurosporine (10 nM) was used as a positive control for apoptosis in A204 and SJCRH30 cells.

Histopathology

Sarcoma samples were retrieved from the University of Iowa Department of Pathology with previous approval from the Institutional Review Board. The tissue microarray was constructed by arraying 1.0 mm cores taken from formalin fixed paraffin embedded tissue and assembled using a MTA-1 tissue arrayer from Beecher Instruments (Sun Prairie, WI) as previously described (48). Sarcomas were classified according to World Health Organization criteria (1).

Expression constructs

Double Flag TAZ and TAZ4SA were derived as previously described in (49). TAZ S58/62A mutations were introduced to Double Flag TAZ by site directed mutagenesis using the QuikChange II Site-directed mutagenesis kit (Agilent #200521) and the following primers:

TAZ S58A Primer – Forward

5' – ctttctttaaggagcctgatgcgggctcgcactc – 3'

TAZ S58A Primer – Reverse

5' – gagtgcgagcccgcatcaggctccttaaagaaag – 3'

TAZ S62A Primer – Forward

5' – cgggctcgcacgcgcgccagtcc – 3'

TAZ S62A Primer – Reverse

5' – ggactggcgcgcgtgcgagcccg – 3'

Transfection and retroviral transduction

Retroviral transduction with pBabeNeo constructs was performed by transfecting PhoenixA retroviral packaging cells with 10 μg of plasmid DNA. Transfection was done using Lipofectamine Reagent and Plus Reagent (Invitrogen-Life Technologies) according to manufacturer's instructions. Supernatant was collected at 48 and 72 hours after transfection, filtered with 0.45 μm filters and supplemented with 8 μg/mL polybrene (EMD Millipore, Burlington, MA, USA). Serial transductions (48 and 72-hour supernatants) were applied to either SJCRH30 or A204 cells for 8 hours each. Pooled stable lines were generated by selecting with G418 for two weeks.

siRNA knockdown

On-TARGETplus SMARTPOOL siRNAs were obtained from Dharmacon and transfected according to the manufacturer's protocol. A204 and SJCRH30 cells were grown to 60-70% confluence in 6cm tissue culture plates. Each plate received 5.5 µL of 20 µM On-TARGETplus SMARTPOOL (Dharmacon) siRNA (AKT1, cat# L-003000-00-0005, PIK3CA, cat# L-003018-00-0005, PDK1, cat# L-003017-00-0005) that was forward transfected with Lipofectamine® RNAiMAX (cat# 13778075) according to manufacturer's protocol. Target cells treated with RNAi- Lipofectamine® RNAiMAX duplexes were incubated for 12, 24, or 48hrs before harvesting for western blot analysis.

RNA interference-mediated silencing

The following pLKO.1-puromycin constructs were obtained from Sigma-Aldrich. Empty vector construct (SHC001) scrambled negative control (SHC002), and TAZ knock-down constructs TRCN0000319224 (shTAZ#3), TRCN0000370007 (shTAZ#5). YAP knock-down constructs were obtained from Addgene #42540 (shYAP#1), #42541 (shYAP1#2). Constructs were transfected into LentiX (HEK293T) cells, along with pcMVΔ8.12 and pVSVG packaging plasmids, using Lipofectamine Reagent and Plus Reagent (Invitrogen) according to manufacturer's instructions. Supernatants were collected at 48 hours, filtered using 0.45 µm filters, and 40% polyethylene glycol (PEG) 8000 was added to a final concentration of 12%, and stored at 4 °C overnight. The following day, 48-hour supernatants were centrifuged, and viral pellets were resuspended in 0.45 µm filtered 72-hour media and 8 µg/mL polybrene (EMD Millipore, Burlington, MA, USA) added to either SJCRH30 or A204 overnight. Pooled stable lines were generated by selection in puromycin.

Western blot

Cell pellets were lysed in radioimmunoprecipitation assay (RIPA) buffer, containing cOmplete™ Protease Inhibitor Cocktail (EDTA-free) (Roche) and PhosSTOP™ Phosphatase Inhibitor Cocktail (Roche) according to the manufacturer's instructions. Total protein concentration was measured using Pierce BCA™ Protein Assay Kit (ThermoFisher Scientific, Waltham, MA, USA). Between 20 μg and 100 μg of total protein was loaded onto a gradient (4-15%) polyacrylamide gel (BioRad, Hercules, CA, USA). Proteins were then transferred to a polyvinylidene difluoride (PVDF) membrane and probed with antibodies described below. Each experiment was repeated at least twice. ImageJ (NIH) was used to quantitate protein densitometry. Fold change expression in western blots was determined by normalization to loading control and comparison to vehicle control (DMSO) or non-targeting controls.

Nuclear and cytoplasmic fractionation

Nuclear and cytoplasmic fractionation was performed with the Nuclear Extract Kit (Active Motif, 40010). Cells collected from two 10cm cell culture plates that were centrifuged and resuspended in 500 μL 1X Hypotonic buffer and incubated for 5 min on ice. 25 μL of detergent was added to resuspended cells which were subsequently vortexed for 10s. After centrifugation, the supernatant cytoplasmic fraction was immediately processed for western blot analysis (see above). Remaining nuclear pellet was resuspended in 50-100 μL complete lysis buffer followed by pestle-motor vortexing for maximal nuclear protein extraction. After centrifugation, the supernatant nuclear fraction was immediately processed for western blot analysis.

Antibodies for Western Blot and Histopathology

Anti-FLAG antibody (mouse monoclonal clone M2 (catalog # F3165) utilized for western blot (1:1000) was obtained from Sigma-Aldrich (St. Louis, MO, USA). Anti-β-actin antibody (AC-15;

catalog #A544) utilized for western blot (1:10,000) was obtained from Sigma-Aldrich (St. Louis, MO, USA). Anti-alpha tubulin antibody (clone DM1A, catalog #T9026) was utilized for western blot (1:5000) was obtained from Sigma-Aldrich (St. Louis, MO, USA). Anti-TAZ (catalog# HPA007415) utilized for western blot (1:2000) was obtained from Sigma-Aldrich (St. Louis, MO, USA). YAP (D8H1X XP; catalog #14074) (1:1000), GAPDH (D16H11;catalog#5174) XP (1:5000), Phospho-S6 Ribosomal Protein (Ser235/236)(D57.2.2E;catalog#4858)(1:2000), S6 Ribosomal Protein (5G10;catalog#2217)(1:2000) ,Phospho-AKT (Ser473) (D9E; catalog# 4060) XP (1:500), AKT (pan) (11E7;catalog# 4685) (1:500), PTEN (D4.3;catalog# 9188) XP (1:500), Phospho-YAP/TAZ(Ser127)(catalog#4911)(1:1000),Phospho-PRAS40(Thr246) (C77D7;catalog#2997) (1:2000) ,PRAS40 (D23C7;catalog# 2691)(1:2000), PI3 Kinase p110α (C73F8;catalog#4249)(1:1000), and Vinculin (E1E9V;catalog#13901)(1:5000) were obtained from Cell Signaling (Danvers, MA, USA) and used for western blot analysis. PDPK1(E-3) (catalog #sc-17765) (1:1000) used for western blots was obtained from Santa Cruz Biotechnology. RNA Pol II (4H8;catalog# 39097) (1:2000) was obtained from Active Motif.

The following antibodies were utilized for immunohistochemistry on formalin fixed paraffin embedded tissue. Anti phospho-AKT Ser473 (rabbit monoclonal D9E; Cell Signaling Technology catalog #4060 (Danvers, MA, USA). Anti-PTEN (rabbit monoclonal D4.3 XP; catalog# 9188) was obtained from Cell Signaling Technology. Anti-TAZ (catalog #BD560235). Anti-YAP (catalog #sc-15407). Dilutions utilized for immunohistochemistry are as follows phospho-AKT (1:50), PTEN (1:50), anti-TAZ (1:50), anti-YAP (1:100). Horseradish peroxidase-conjugated secondary antibodies for immunohistochemistry were obtained from Dako. Horseradish peroxidase-conjugated secondary antibodies used for western blots were obtained from Bethyl Laboratories (catalog# A120-101P; A90-116P) and used at 1:5,000 or 1:10,000.

Soft agar assay

The base layer of 0.5% agarose was plated into 6-well plates (2 mLs/well) and allowed to solidify for 1 hour. For SJCRH30 cells, 2 x 10^3 cells/2 mL in 0.35% agarose was added to form the top layer. Plates were left in the hood and allowed to solidify at room temperature for 3 hours. Colonies were allowed to grow for 2-3 weeks at 37 °C and 5% CO_2 before imaging. Each experiment was repeated at least twice.

Clonogenic assay

100-500 cells were seeded in a 6 well plate format. The following day cells were treated with varying concentrations of LY294002, GDC-0941, or DMSO for 72 hours. After treatment, drug containing media was removed and replaced with fresh media and outgrowth was monitored for 2-3 weeks. Colonies were then washed with 1X PBS twice and cells were fixed and stained with 0.5% crystal violet for 30 minutes. Plates were rinsed, dried, and colonies were subsequently counted. Survival fraction= (# of colonies formed post-treatment)/(# of cells seeded). Cells are plated prior to treatment, controlling for plating efficiency across the different conditions.

Mouse studies

All animal procedures were performed with approval from the University of Iowa Institutional Animal Care and Use Committee.

Experiment 1 (Figure 4)

Both the *Pten* and *Trp53* floxed alleles were bred to homozygosity as previously described in (80). Offspring were genotyped by PCR for the presence of the *Trp53*$^{fl/fl}$ and *Pten*$^{fl/fl}$ alleles using gene-specific primers. *Wwtr1*$^{fl/fl}$*Yap1*$^{fl/fl}$ mice (124) were generously provided by Dr. Eric Olson (University of Texas Southwestern Medical Center, Dallas, TX) and crossed with *Trp53*$^{fl/fl}$*Pten*$^{fl/fl}$ mice to generate *Trp53*$^{fl/fl}$*Pten*$^{fl/fl}$*Wwtr1*$^{fl/fl}$, *Trp53*$^{fl/fl}$*Pten*$^{fl/fl}$*Yap1*$^{fl/fl}$, and

Trp53$^{fl/fl}$*Pten*$^{fl/fl}$*Wwtr1*$^{fl/fl}$*Yap1*$^{fl/fl}$ mice. 8-12 week old mice were used for the study, with approximately 50% female and 50% male mice.

Experiment 2 (Figure 5)

For the everolimus study, 8-12 week old *Trp53*$^{fl/fl}$*Pten*$^{fl/fl}$ mice were injected with adeno-Cre virus as described above and separated into treatment and vehicle control arms composed of 16 mice, with approximately equal numbers of female and male mice used. 100 µL of 5mg/kg everolimus or vehicle (DMSO) was delivered by oral gavage on a weekly basis (81).

Experiment 3 (Figure 8)

For the combination drug study, *Prkdc*^{scid}*Il2rg*^{tm1Wjl}*/SzJ* (NSG) (RRID:IMSR_JAX:005557) were obtained from The Jackson Laboratory (Bar Harbor, ME). NSG mice ranging in age from 8-10 weeks old were utilized in the SJCRH30 xenograft/combination therapy study. $5x10^6$ SJCRH30 cells or A204 cells in 100 µL 1X PBS were injected subcutaneously IK-930 (75 mg/kg daily), everolimus (5 mg/kg weekly), or the combination of the two were administered by oral gavage to the respective treatment arms of their study.

Adenovirus injection:

For the experiment evaluating sarcoma formation and overall survival in *Trp53*$^{fl/fl}$*Pten*$^{fl/fl}$, *Trp53*$^{fl/fl}$*Pten*$^{fl/fl}$*Wwtr1*$^{fl/fl}$, *Trp53*$^{fl/fl}$*Pten*$^{fl/fl}$*Yap1*$^{fl/fl}$, and *Trp53*$^{fl/fl}$*Pten*$^{fl/fl}$*Wwtr1*$^{fl/fl}$*Yap1*$^{fl/fl}$ mice, 20 mice were included for each genotype and injected with Ad5 CMV-Cre recombinase virus, while 4 mice (1 from each genotype) were injected with Ad5 CMV-eGFP virus for a total of 84 mice examined. No tumors developed in Adeno CMV-eGFP control mice. The experiment was repeated. *2P*, *2PY*, *2PW* and *2PWY* mice were first anesthetized in a chamber using 3% isoflurane in order to facilitate accurate injections. Ad5 CMV-eGFP (control animals) or Ad5 CMV-Cre recombinase (experimental animals) obtained from The University of Iowa Viral Vector Core was

injected intramuscularly into the posterior left quadriceps. Injections were performed using $1x10^9$ pfu in a total of 20 µL injection volume (viral stocks were diluted to final concentration using 1x phosphate-buffered saline). Successful injections were confirmed either by manual stabilization of the quadriceps muscle and palpation of the femur with the needle tip prior to injection in the intramuscular group.

Mouse examinations

Once tumors were palpable, they were monitored/measured every 2-3 days until completion of the study. Tumor volume was estimated using the equation $V = 0.5 x L x W^2$ (V=volume, L=length, W=width). Euthanasia and dissections were performed when mice reached pre-determined end points, including 20% loss of starting body weight, primary tumor size 1.5 cm (Experiment 1 – see above Mouse studies section) or 2.0 cm (Experiments 2, 3 – see above Mouse studies section) at the site of injection, decreased mobility, lethargy/lack of grooming, or other gross morbidity. Control mice were also euthanized at the completion of the study. Primary tumors and other appropriate tissues were collected and incubated in 10% neutral buffered formalin and embedded in paraffin prior to sectioning.

Combination therapy mouse studies

NOD scid gamma (NSG) mice were obtained from The Jackson Laboratory (Bar Harbor, ME, USA) and carry the strain NOD.Cg-Prkdcscid Il2rgtm1Wjl/SzJ (RRID:IMSR_JAX:005557). All animal work was approved by the University of Iowa Institutional Animal Use and Care Committee. SJCRH30 cells or A204 cells containing (5×10^6 cells/100 µL PBS) were injected subcutaneously into the NSG mice. The mice were 8-10 weeks of age at the time of injection.

RNA-Seq

Total RNA was extracted using Trizol Reagent (Ambion-Life Technologies). Total RNA was isolated using the PureLink RNA mini kit (Invitrogen-ThermoFisher Scientific). On-column DNase (Invitrogen) treatment was performed according to manufacturer's instructions. Transcription profiling using RNA-Seq was performed by the University of Iowa Genomics Division using manufacturer recommended protocols. Initially, 500 ng of DNase I-treated total RNA was used as input for library preparation. The enriched total RNA pool was then fragmented, converted to cDNA and ligated to sequencing adaptors containing indexes using the Illumina TruSeq Stranded Total RNA w/ RiboZero sample preparation kit (Cat. No. RS-122-2201, Illumina, Inc, San Diego, CA). The molar concentrations of the indexed libraries were measured using the 2100 Agilent Bioanalyzer (Agilent Technologies, Santa Clara, CA) and combined equally into pools for sequencing. The concentration of the pools was measured using the Illumina Library Quantification Kit (KAPA Biosystems, Wilmington, MA) and sequenced on the Nova Seq 6000 genome sequencer using 150 bp paired-end SBS chemistry.

Barcoded samples were pooled and sequenced using an Illumina NovaSeq 6000 in the Iowa Institute of Human Genetics (IIHG) Genomics Core Facility. Paired-end reads were demultiplexed and converted from the native Illumina BCL format to FASTQ format using a custom python workflow wrapper to Illumina's 'bcl2fastq' conversion utility. FASTQ data were processed with nf-core/rnaseq (v3.10), a best-practices pipeline available at the open-source 'nf-core' project (125) (https://nf-co.re/, Nextflow version 22.10) (126) running on the Argon HPC resource at the University of Iowa. The pipeline was invoked with command:

nextflow run nf-core/rnaseq -r 3.10 -profile argon --igenomes_base /Users/mchiment/igenomes/references --input samplesheet_NOSKEL4.csv --outdir nf_NOSKEL4 --genome GRCm38 --email michael-chimenti@uiowa.edu --save_merged_fastq FALSE --skip_markduplicates TRUE --skip_preseq TRUE --skip_dupradar TRUE --skip_stringtie TRUE.

Reads from the samples were aligned against the mouse reference genome ‘GRCm38’ using the STAR aligner (127) and quantified with ‘salmon’ (128). Samtools (129) was used in conjunction with Qualimap (130) (131) and MultiQC {10.1093/bioinformatics/btw354} to inspect alignment results . Length-normalized gene-level counts from the STAR/salmon pipeline were used for differential gene expression analysis with DESeq2 (132). Bioconductor package ‘PCAExplorer’ (133) was used for exploratory analysis. The DE gene lists were analyzed using AdvaitaBio’s iPathwayGuide (https://www.advaitabio.com/ipathwayguide) (134) (135) (136) (137). This software analysis tool implements the ‘Impact Analysis’ approach that takes into consideration the direction and type of all signals on a pathway. The raw FASTQ files and associated metadata have been made available for download at GEO accession: GSE274982.

Immunofluorescence

For both SJCRH30 and A204 cell lines, integrated density of nuclear signal for YAP or TAZ (ImageJ software) was taken for 10 cells from four different fields. The integrated density for LY294002 treated cells were compared relative to DMSO for final graphical presentation (GraphPad Prism). Since TAZ or YAP signal was more diffuse in both the nuclear and cytoplasmic compartments in the A204 cell line, the integrated density was taken for the entire cell. For both SJCRH30 and A204 cell lines, the fluorescent signal in approximately 100 cells for DMSO and LY294002 treated conditions were counted as predominantly nuclear (N>C), cytoplasmic (C>N), or both (N=C). Percentages for the respective counts were calculated relative to the total number of cells counted.

Luciferase reporter assay

SJCRH30 and A204 cells were transduced with TEAD Luciferase (pLV-8xTEADrep-Luc2-pgk-Rluc-2A-Neo) reporter construct plasmid (Ikena Oncology, Boston, MA) to generate stable cell

lines. A Dual luciferase reporter assay (Promega, Madison, WI, USA) detecting both firefly luciferase and *Renilla* luciferase activity was performed in a six-well plate (biological triplicates) with a seeding density of $5x10^5$ cells/well. Three days after the drug treatment, the cells were collected and lysed, and extracts were assayed in technical triplicate for firefly and *Renilla* luciferase activity using the Dual Luciferase Reporter Assay System (Promega, Madison, WI, USA) and a Synergy H1 Hybrid Multi-Mode Microplate Reader (Biotek, Winooski, VT, USA). Each experiment was repeated at least twice. Due to extremely high luminescence values of *Renilla* control for A204 cells, the firefly luciferase activity values were considered alone after being normalized with the untreated group (DMSO).

Proliferation assay

1000 cells/well from the SJCRH30 and A204 cell lines were plated in a 96 well plate. Drugs were added for 72 hours and proliferation was measured with the Dojindo Assay Cell Counting Kit 8 (Dojindo Molecular Technologies, Rockville, MD) according to the manufacturer's instructions. Absorbance was measured using the BioTek: Synergy H1 Hybrid Reader (Biotek, Winooski, VT, USA). Each experiment was repeated at least twice.

References:

124. Xin M, et al. Hippo pathway effector Yap promotes cardiac regeneration. *Proc Natl Acad Sci U S A.* 2013;110(34):13839-44.
125. Ewels PA, et al. The nf-core framework for community-curated bioinformatics pipelines. *Nat Biotechnol.* 2020;38(3):276-8.
126. Di Tommaso P, et al. Nextflow enables reproducible computational workflows. *Nat Biotechnol.* 2017;35(4):316-9.
127. Dobin A, et al. STAR: ultrafast universal RNA-seq aligner. *Bioinformatics.* 2013;29(1):15-21.
128. Bray NL, et al. Near-optimal probabilistic RNA-seq quantification. *Nat Biotechnol.* 2016;34(5):525-7.
129. Li H, et al. The Sequence Alignment/Map format and SAMtools. *Bioinformatics.* 2009;25(16):2078-9.

130. Garcia-Alcalde F, et al. Qualimap: evaluating next-generation sequencing alignment data. *Bioinformatics.* 2012;28(20):2678-9.
131. Okonechnikov K, et al. Qualimap 2: advanced multi-sample quality control for high-throughput sequencing data. *Bioinformatics.* 2016;32(2):292-4.
132. Love MI, et al. Moderated estimation of fold change and dispersion for RNA-seq data with DESeq2. *Genome Biol.* 2014;15(12):550.
133. Marini F, Binder H. pcaExplorer: an R/Bioconductor package for interacting with RNA-seq principal components. *BMC Bioinformatics.* 2019;20(1):331.
134. Draghici S, et al. A systems biology approach for pathway level analysis. *Genome Res.* 2007;17(10):1537-45.
135. Tarca AL, et al. A novel signaling pathway impact analysis. *Bioinformatics.* 2009;25(1):75-82.
136. Donato M, et al. Analysis and correction of crosstalk effects in pathway analysis. *Genome Res.* 2013;23(11):1885-93.
137. Ahsan S, Draghici S. Identifying Significantly Impacted Pathways and Putative Mechanisms with iPathwayGuide. *Curr Protoc Bioinformatics.* 2017;57:7 15 1-7 30.
