# Supplementary material for: PI3K regulates TAZ/YAP and mTORC1 axes that can be synergistically targeted
Source: JCI Insight. 2026 Feb 10;11(6):e191600. doi: 10.1172/jci.insight.191600 (PMC13043097; doi:10.1172/jci.insight.191600)

Full unedited gel for Figure 2A

Figure 2A SJCRH30 IB: Total AKT

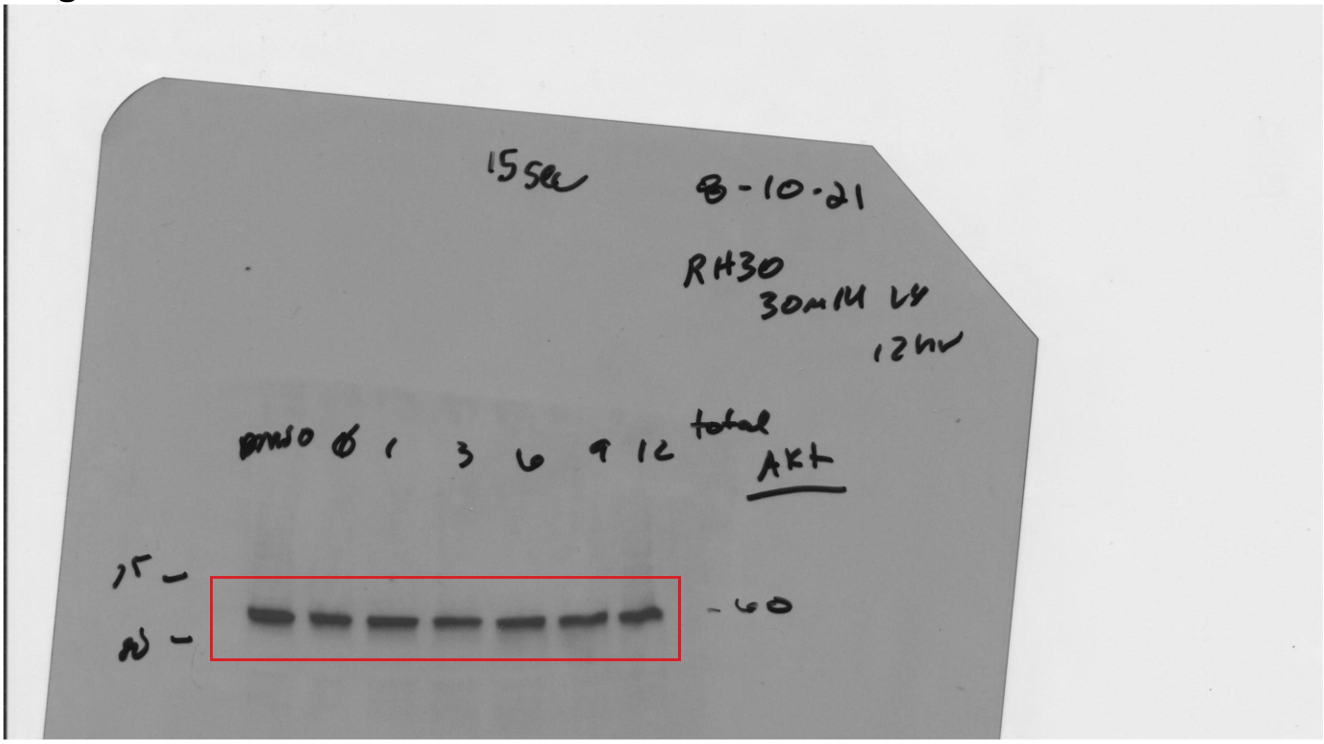

Figure 2A SJCRH30 IB: Phospho-AKT(S473)

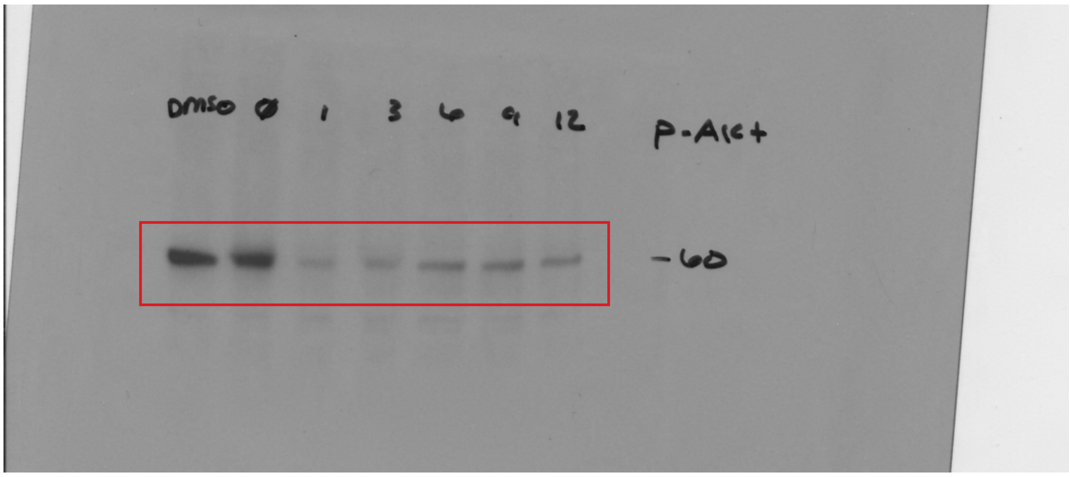

Figure 2A SJCRH30 IB: β-ACTIN

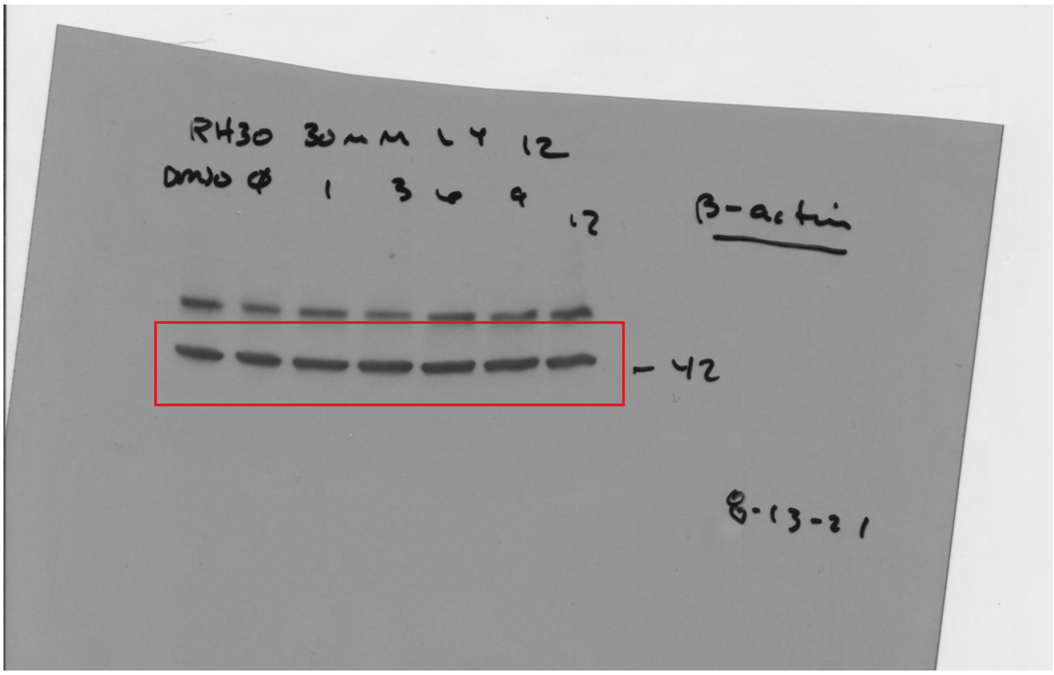

Full unedited gel for Figure 2A

Figure 2A SJCRH30 IB: Total S6

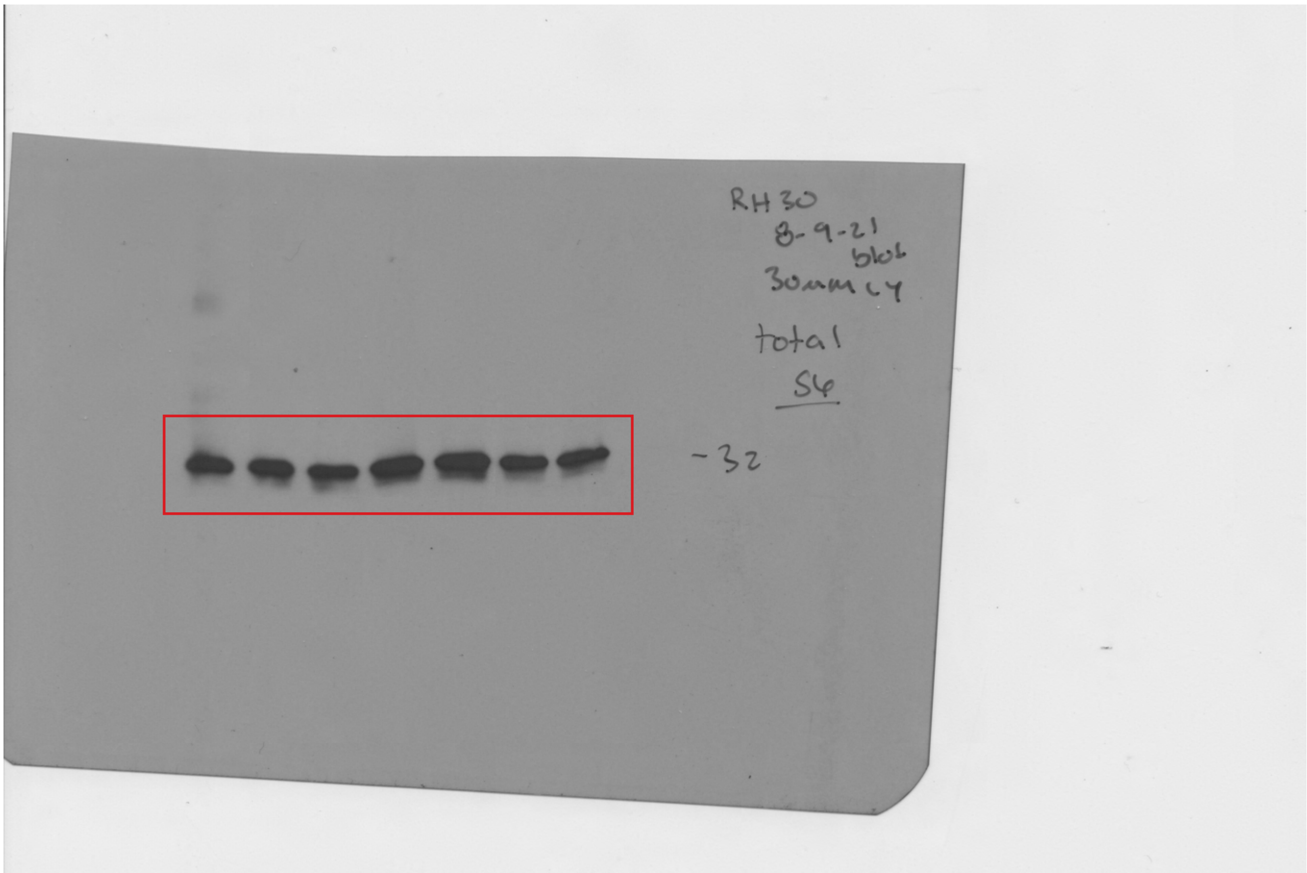

Figure 2A SJCRH30 IB: Phospho-S6 (S235/236)

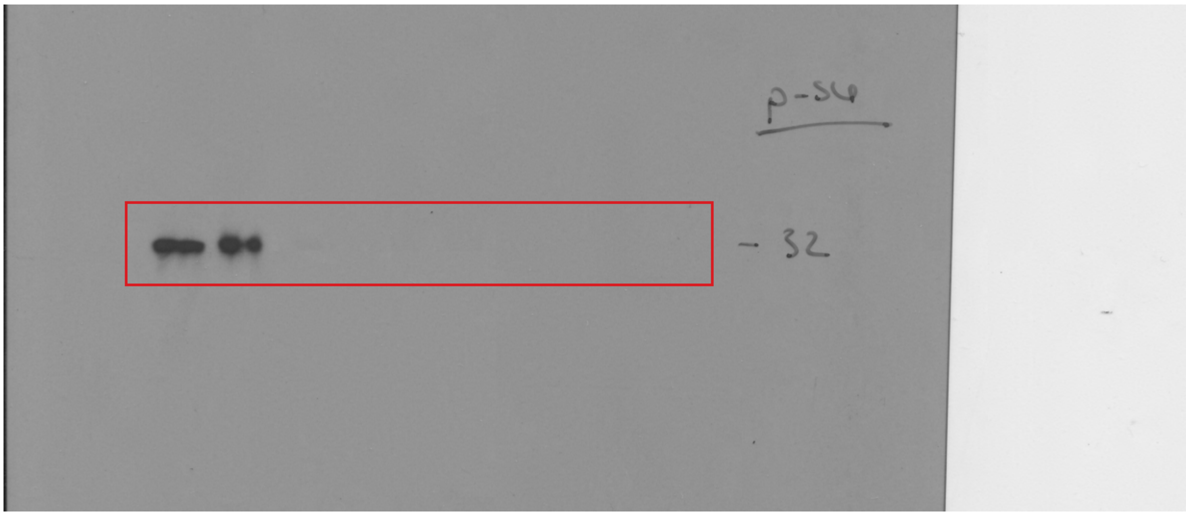

Full unedited gel for Figure 2A

Figure 2A SJCRH30 IB: YAP

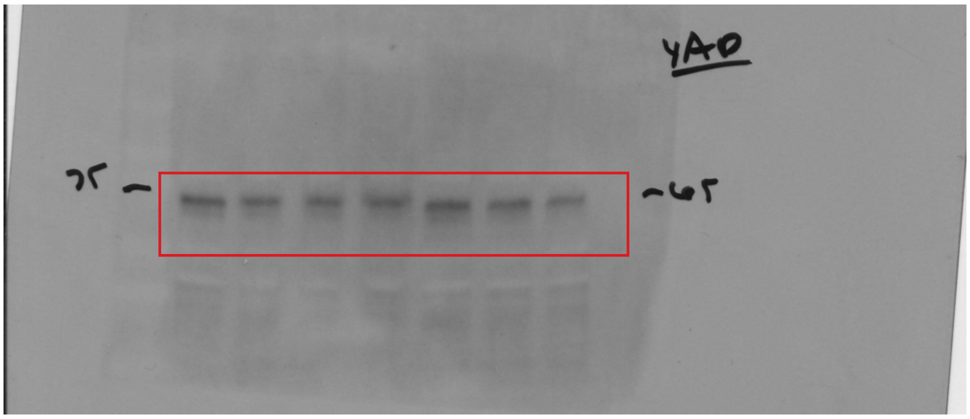

Figure 2A SJCRH30 IB: TAZ

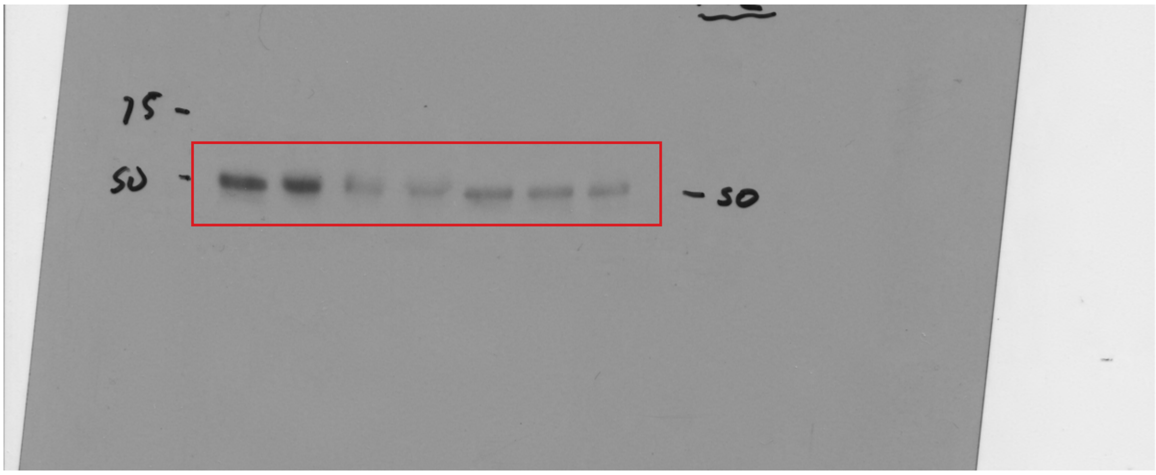

Full unedited gel for Figure 2A

Figure 2A A204 IB: β-ACTIN

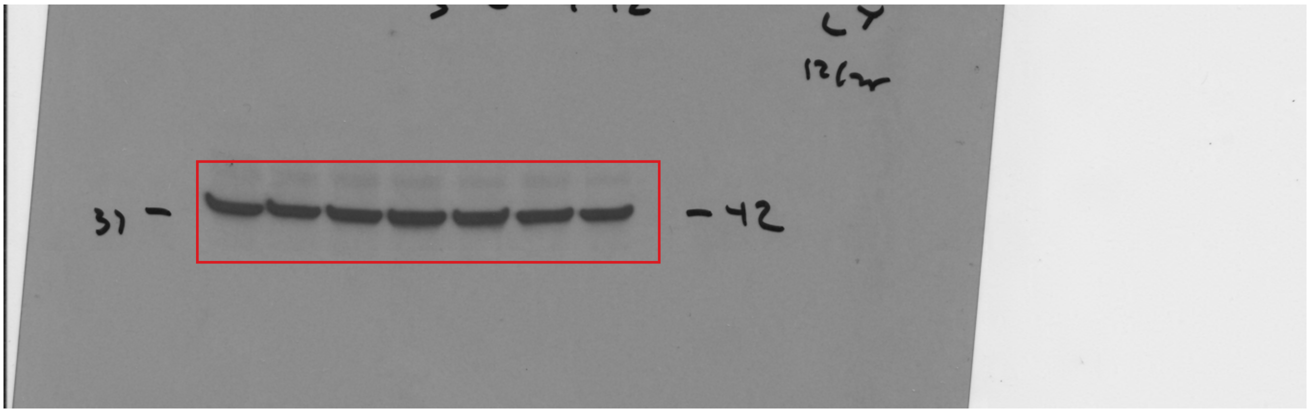

Figure 2A A204 IB: Total S6

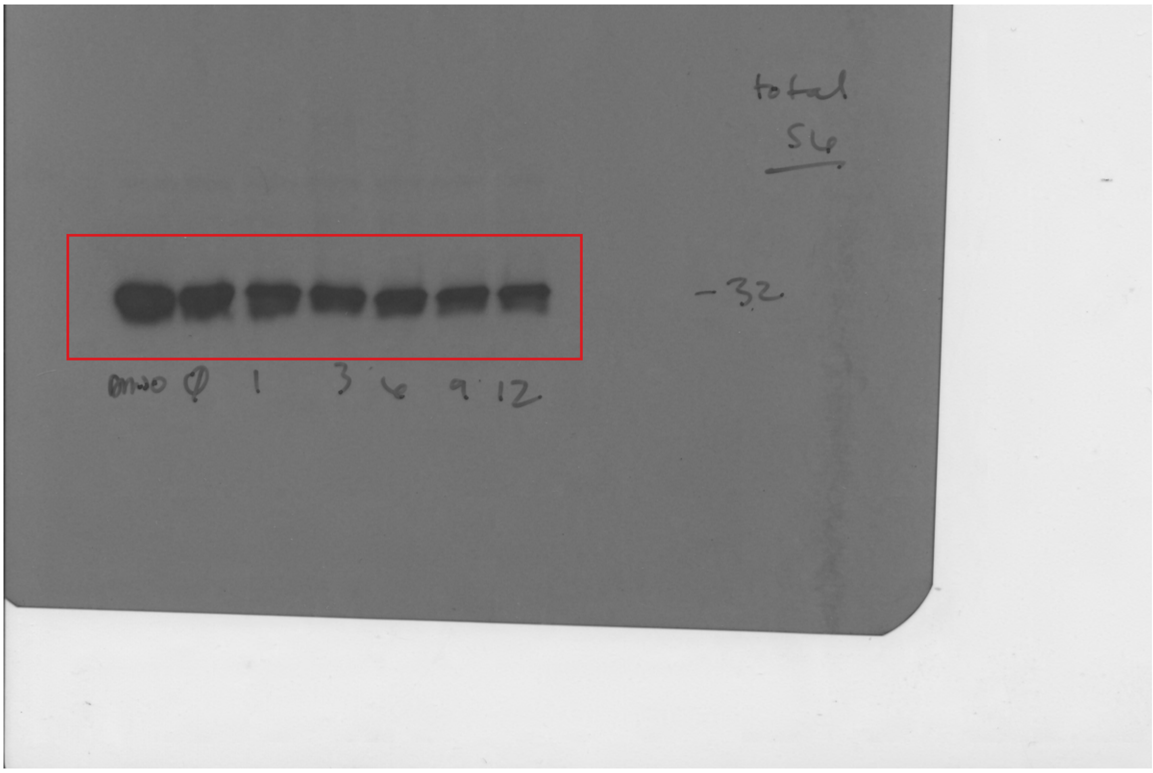

Figure 2A A204 IB: phospho-S6 (S235/236)

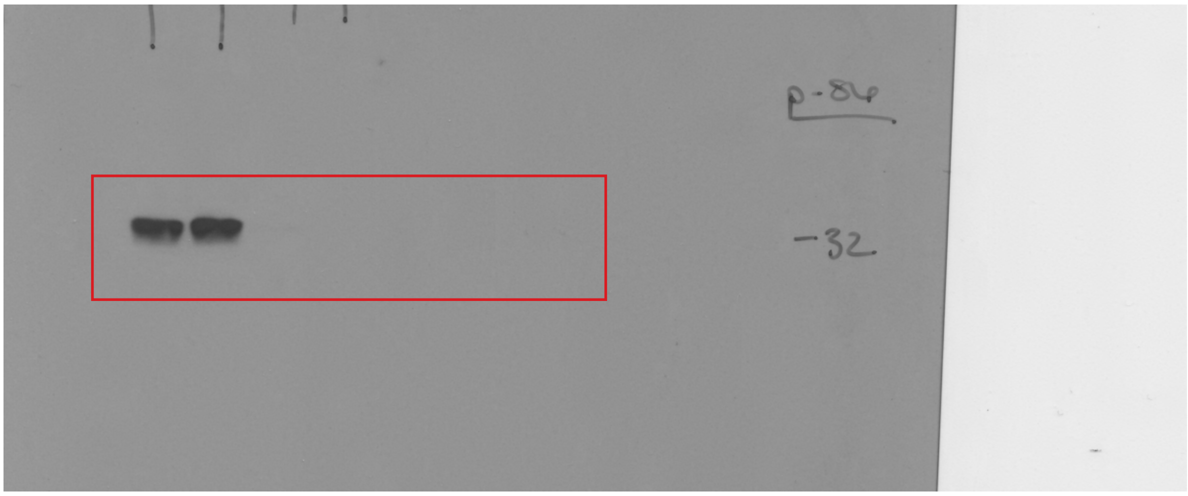

Full unedited gel for Figure 2A

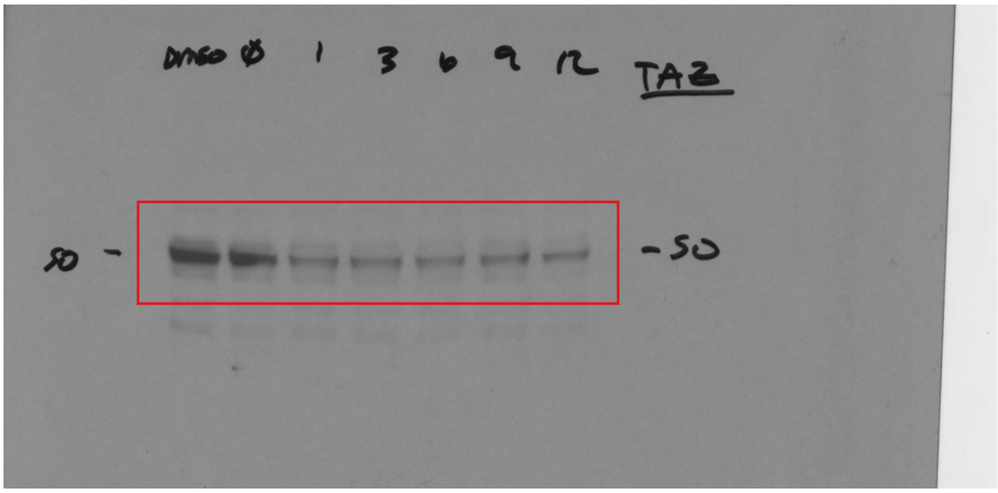

Figure 2A A204 IB: TAZ

Figure 2A A204 IB: YAP

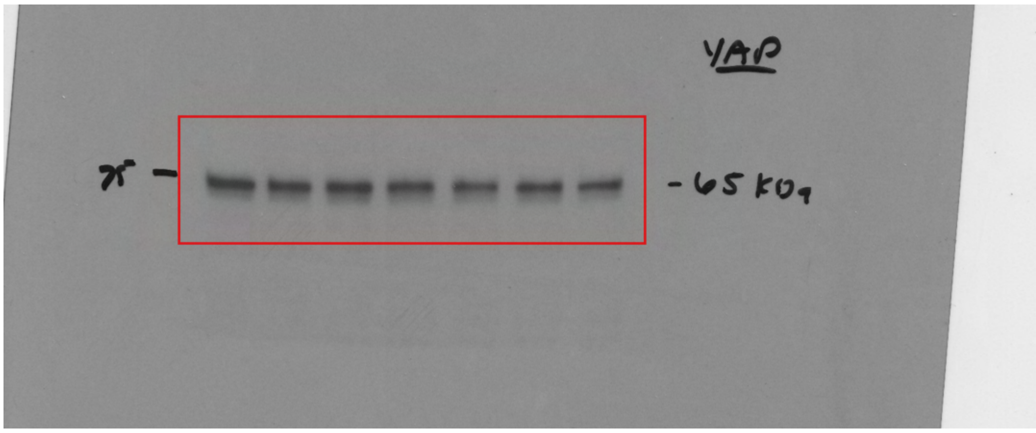

Figure 2A A204 IB: phospho-AKT(S473)

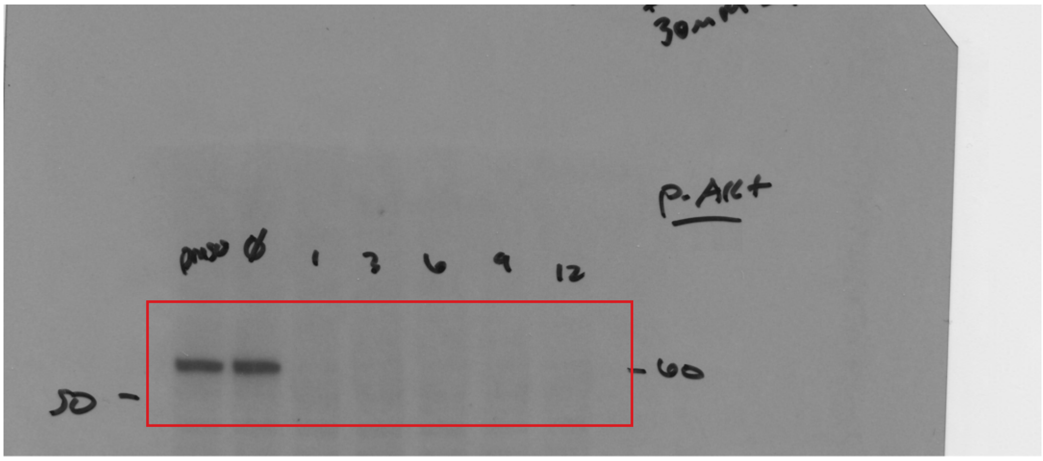

Figure 2A A204 IB:Total AKT

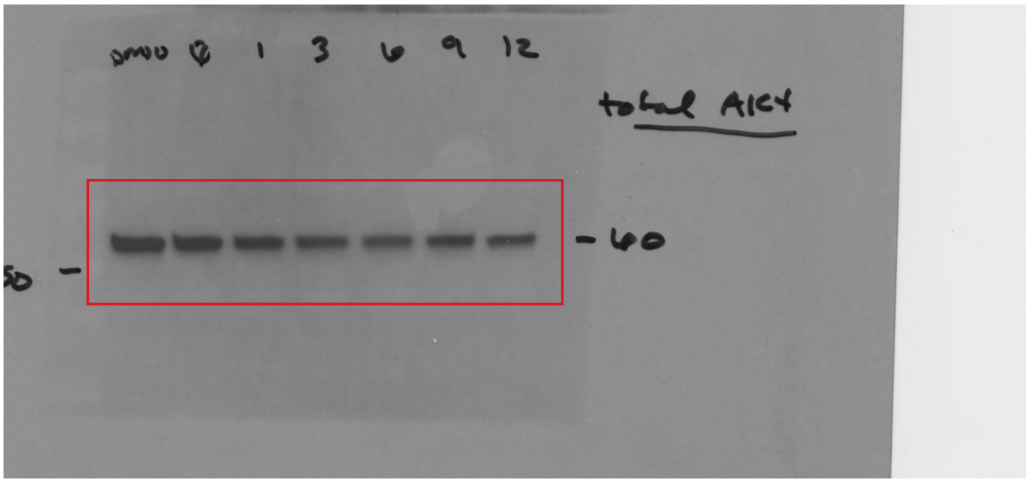

Full unedited gel for Figure 2B

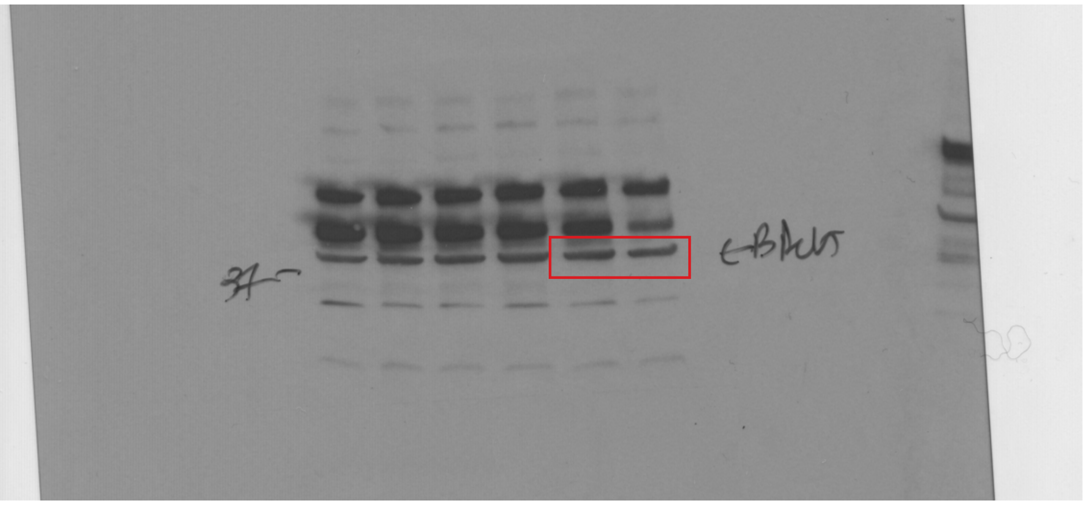

Figure 2B SJCRH30
IB: β-ACTIN

Figure 2B SJCRH30 IB: TAZ

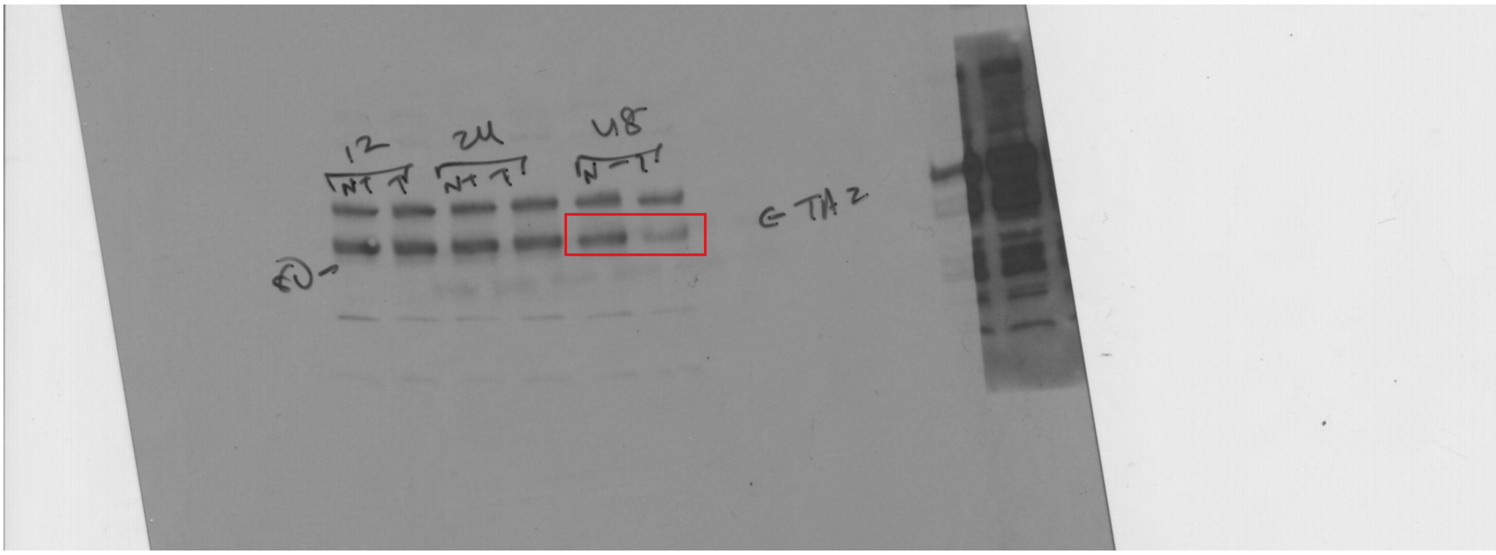

Figure 2B SJCRH30 IB: YAP

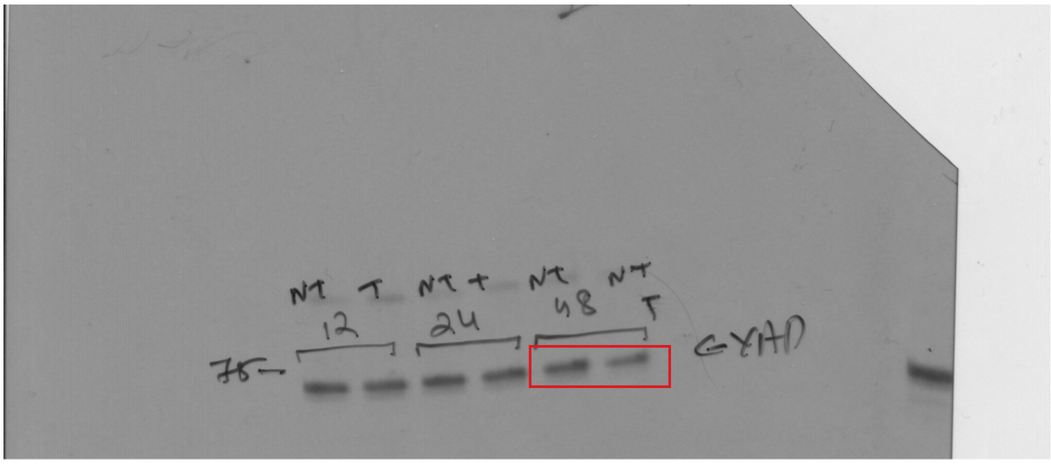

Figure 2B RH30 IB: p110α

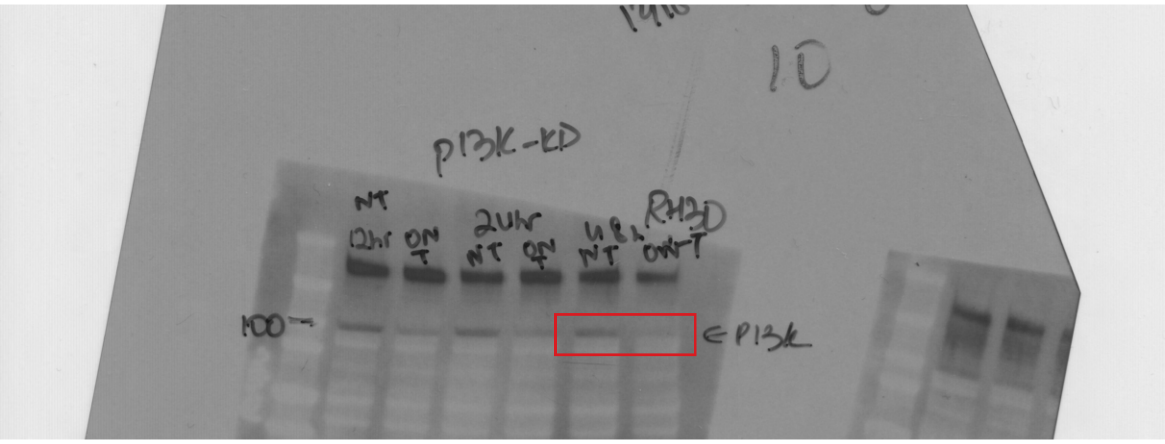

Full unedited gel for Figure 2C

Figure 2C A204 IB: TAZ

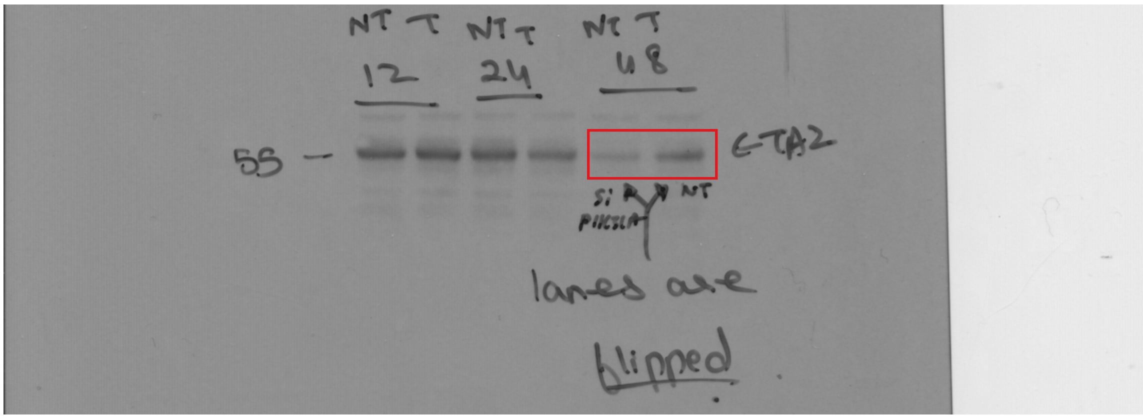

Figure 2C A204 IB: YAP

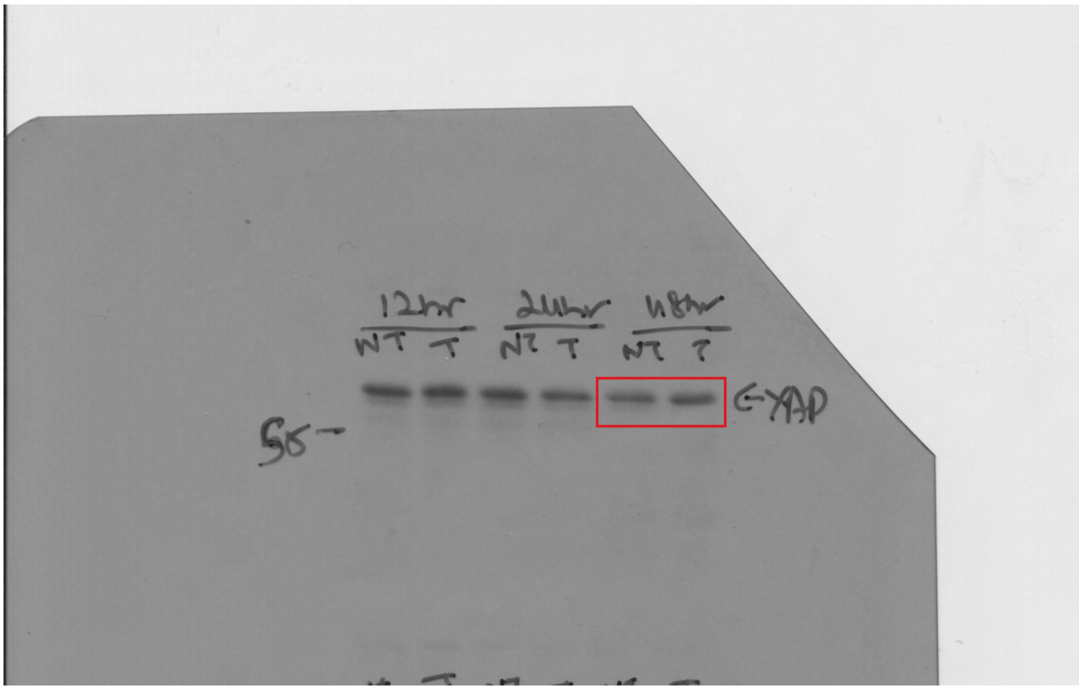

Figure 2C A204 IB: p110α

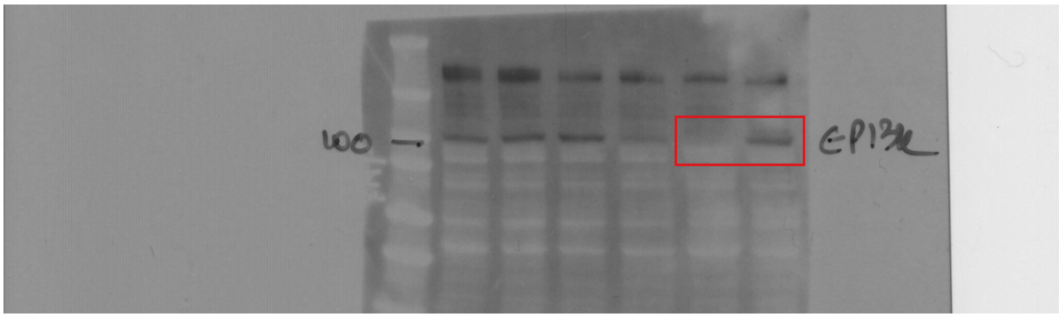

Figure 2C A204 IB: β-ACTIN

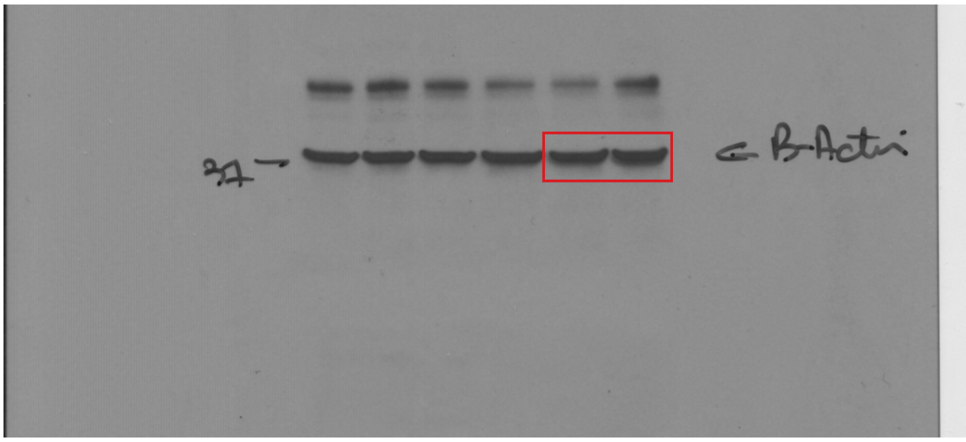

Full unedited gel for Figure 2F

Figure 2F SJCRH30 IB: YAP cytoplasm (left) nuclear (right)

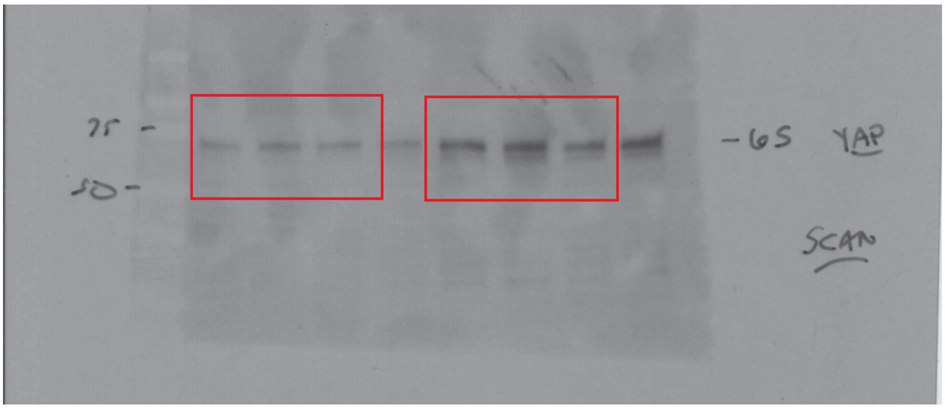

Figure 2F SJCRH30 IB: TAZ cytoplasm (left) nuclear (right)

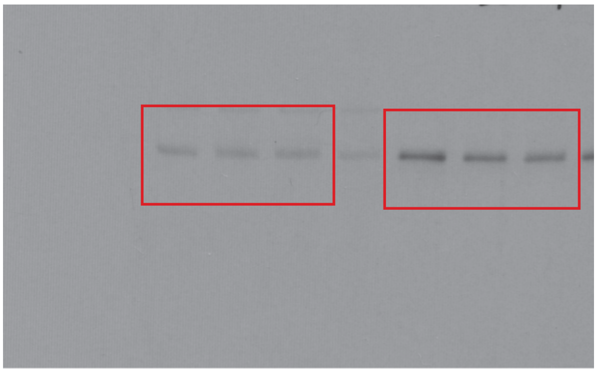

Figure 2F SJCRH30 IB: phospho-TAZ (S89) nuclear (bottom right), phospho-YAP (S127) nuclear (top right)

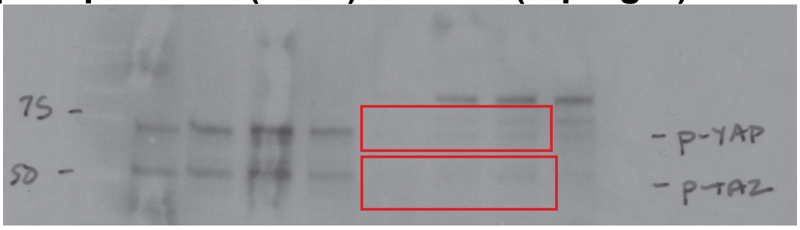

Figure 2F SJCRH30 IB: phospho-TAZ (S89) cytoplasm (bottom), phospho-YAP (S127) cytoplasm (top)

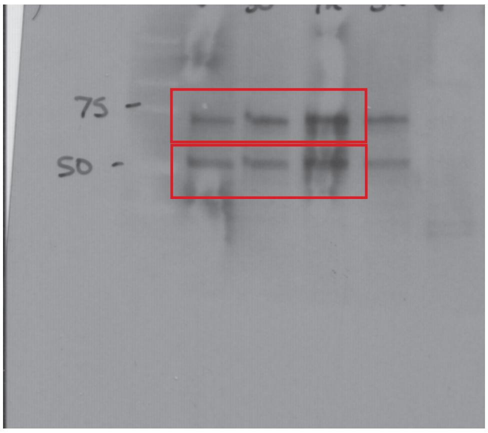

Figure 2F RH30 IB: α-tubulin cytoplasm (left) and nuclear (right)

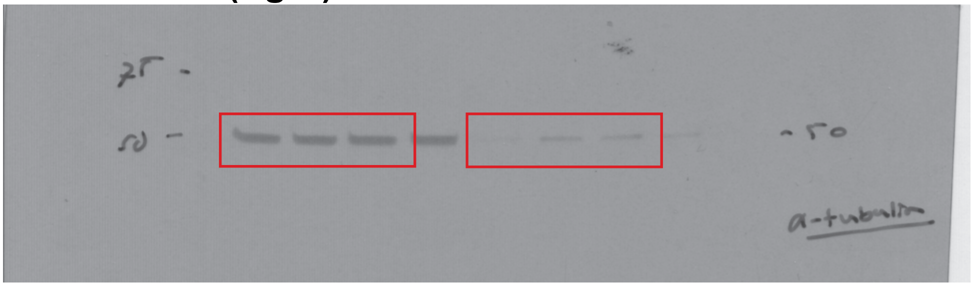

Figure 2F SJCRH30 IB: Pol.II cytoplasm (left) and Pol.II nuclear (right)

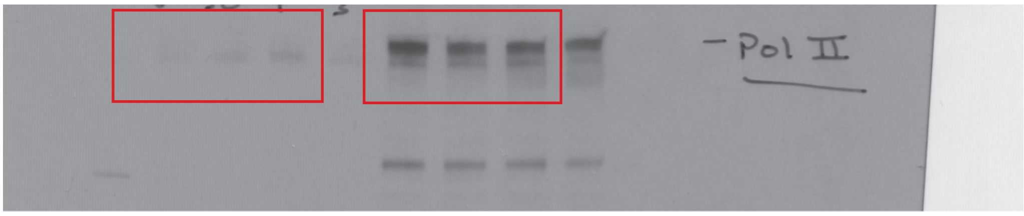

Full unedited gel for Figure 2G

Figure 2G A204 IB: TAZ cytoplasm

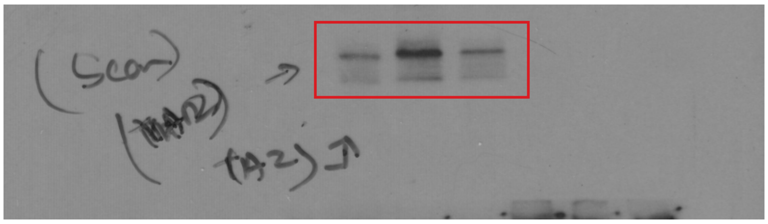

Figure 2G A204 IB: YAP nuclear (top) and TAZ nuclear (bottom)

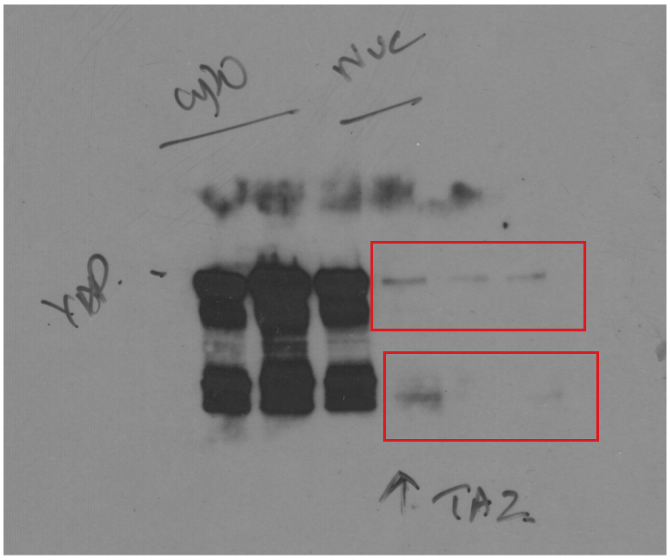

Figure 2G A204 IB: YAP cytoplasm

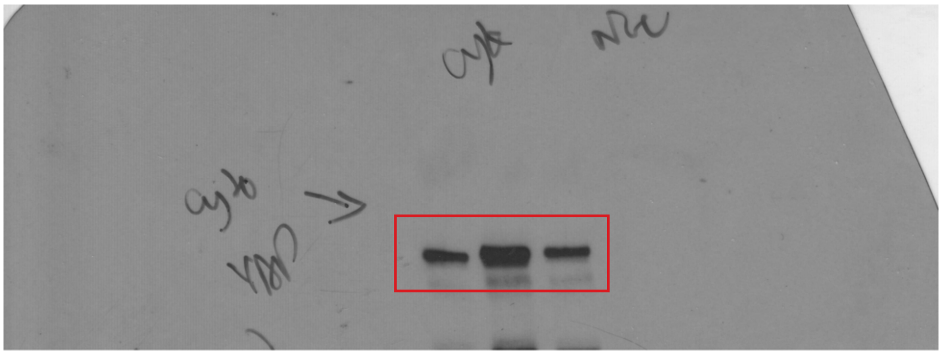

Figure 2G A204 IB: α-tubulin cytoplasm

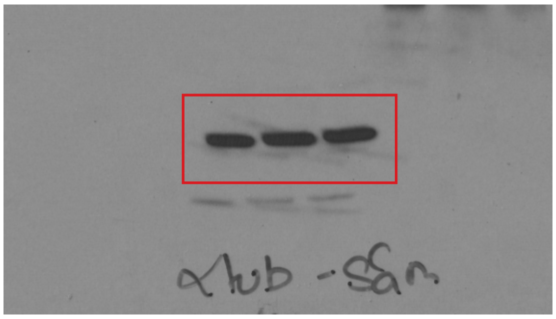

Figure 2G A204 IB: Pol.II cytoplasm (left) nuclear (right)

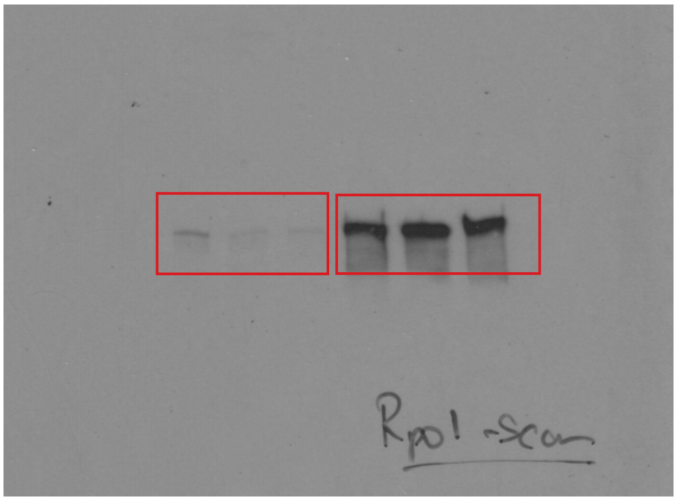

Full unedited gel for Figure 3A

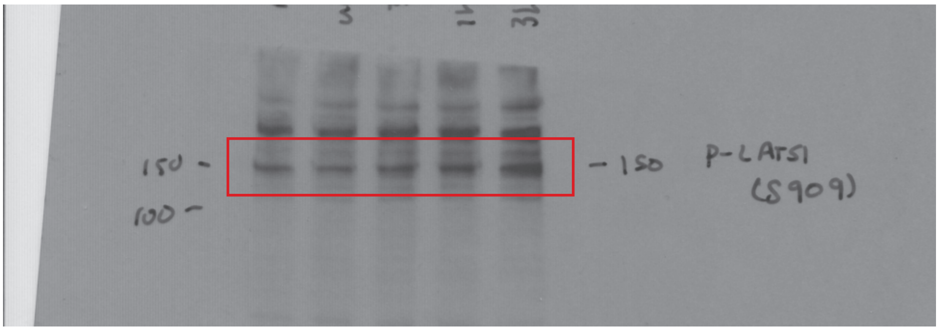

Figure 3A SJCRH30
IB: phospho-LATS1 (S909)

Figure 3A SJCRH30 IB: Total LATS1

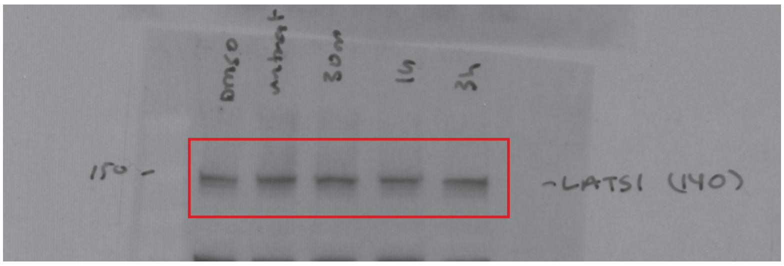

Figure 3A SJCRH30 IB: phospho-YAP (S127) (top) and p-TAZ (S89) (bottom)

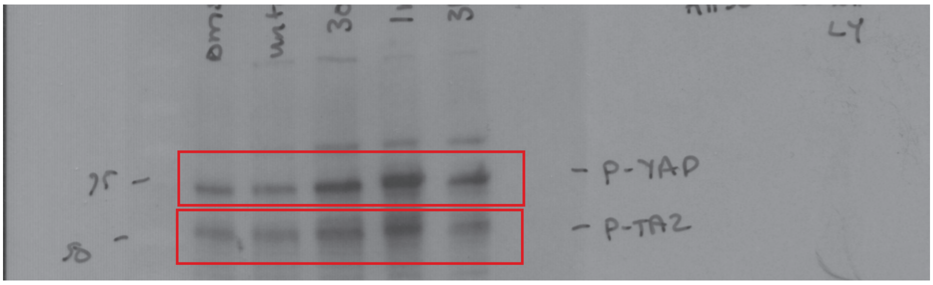

Figure 3A SJCRH30 IB: YAP

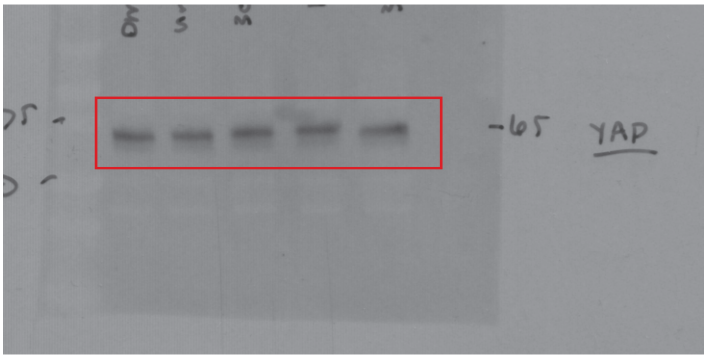

Figure 3A SJCRH30 IB: TAZ

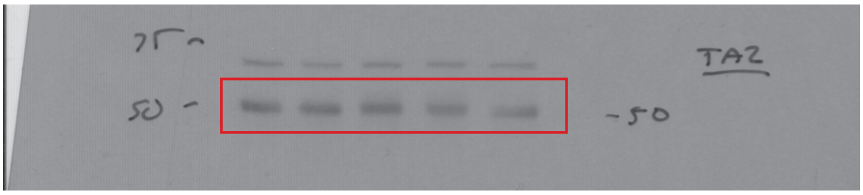

Figure 3A SJCRH30 IB: β-ACTIN

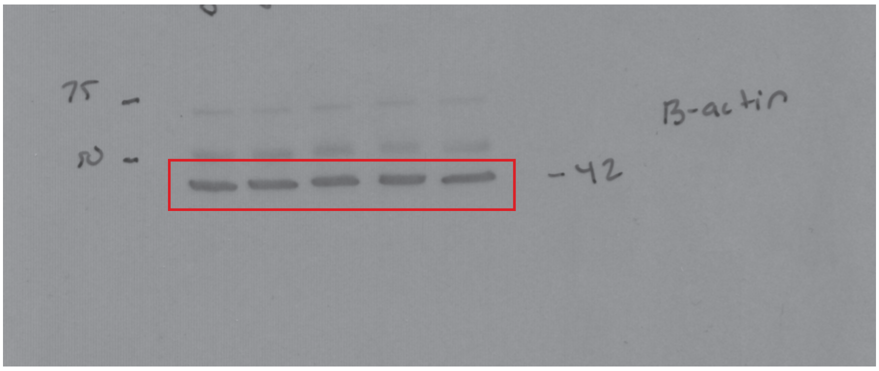

Full unedited gel for Figure 3B-C

Figure 3B A204 IB: Flag-TAZ

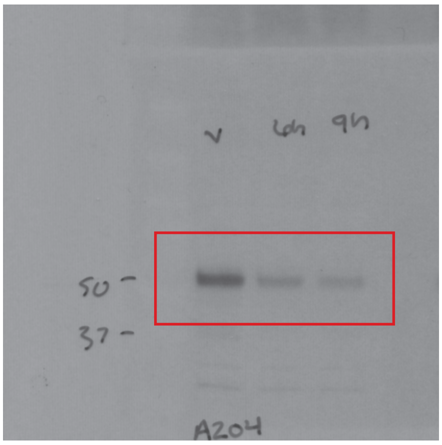

Figure 3B A204 IB: β-ACTIN

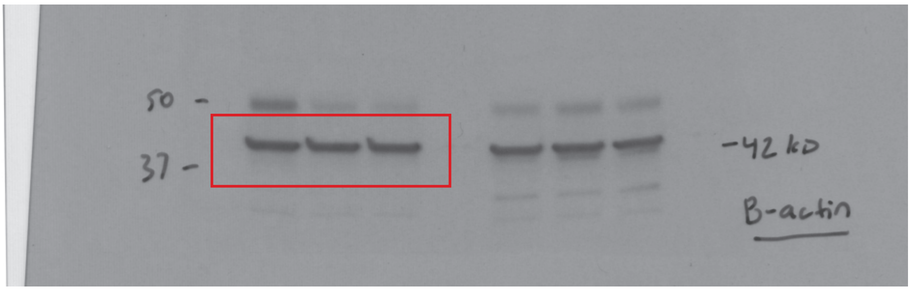

Figure 3C A204 IB: Flag-TAZ4SA

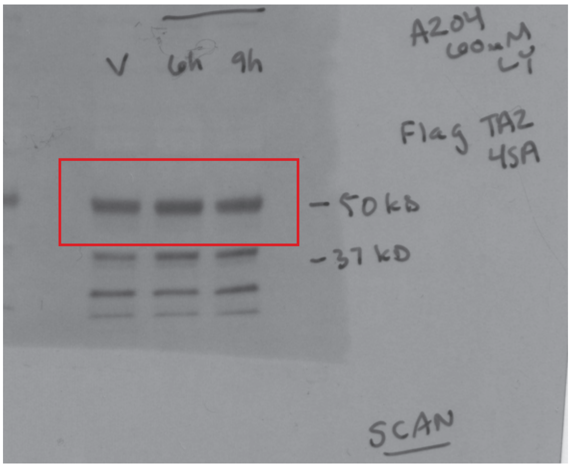

Figure 3C A204 IB: β-ACTIN

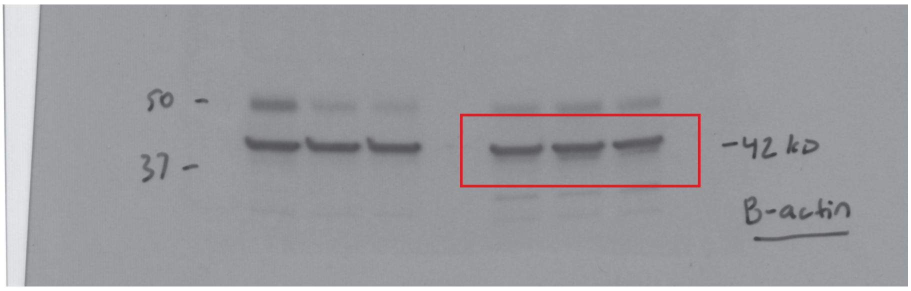

Full unedited gel for Figure 3D

Figure 3D A204 IB: TAZ

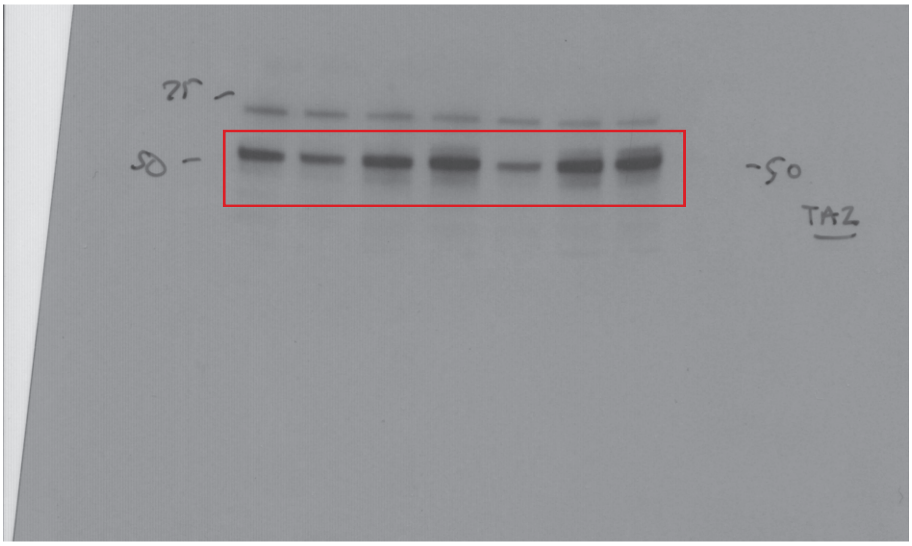

Figure 3D A204 IB: YAP

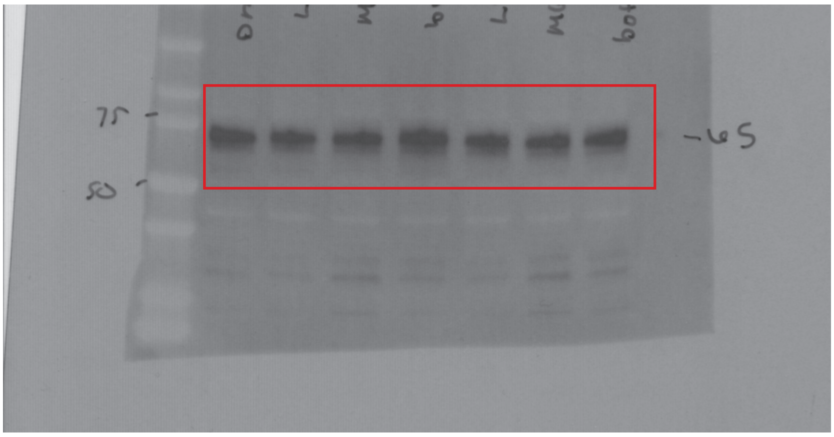

Figure 3D A204 IB: β-ACTIN

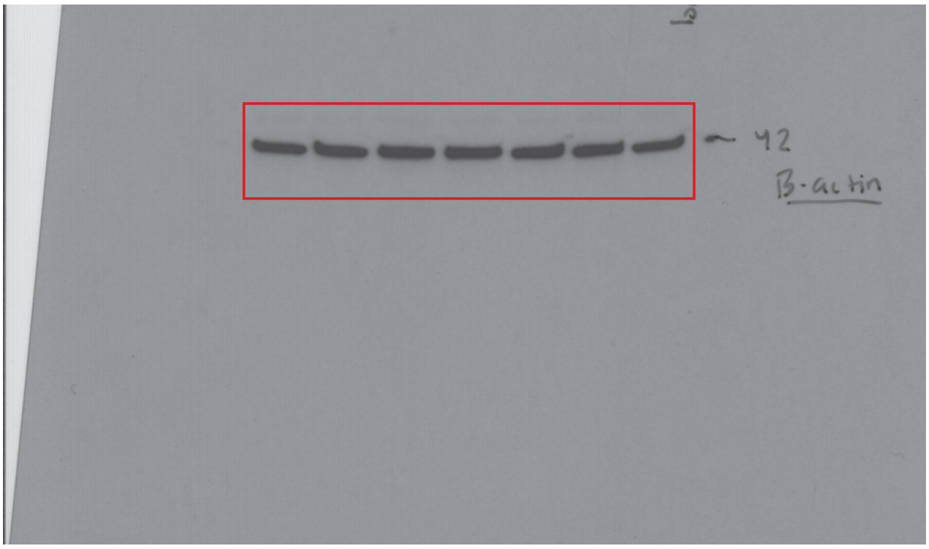

Full unedited gel for Figure 3E

Figure 3E SJCRH30 IB: YAP

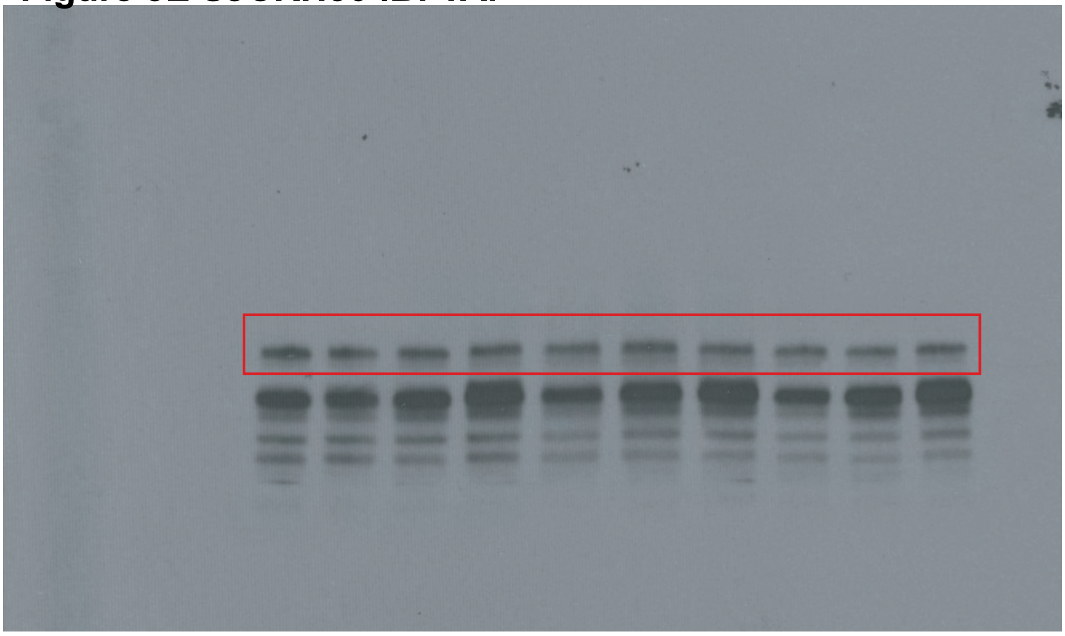

Figure 3E SJCRH30 IB: TAZ

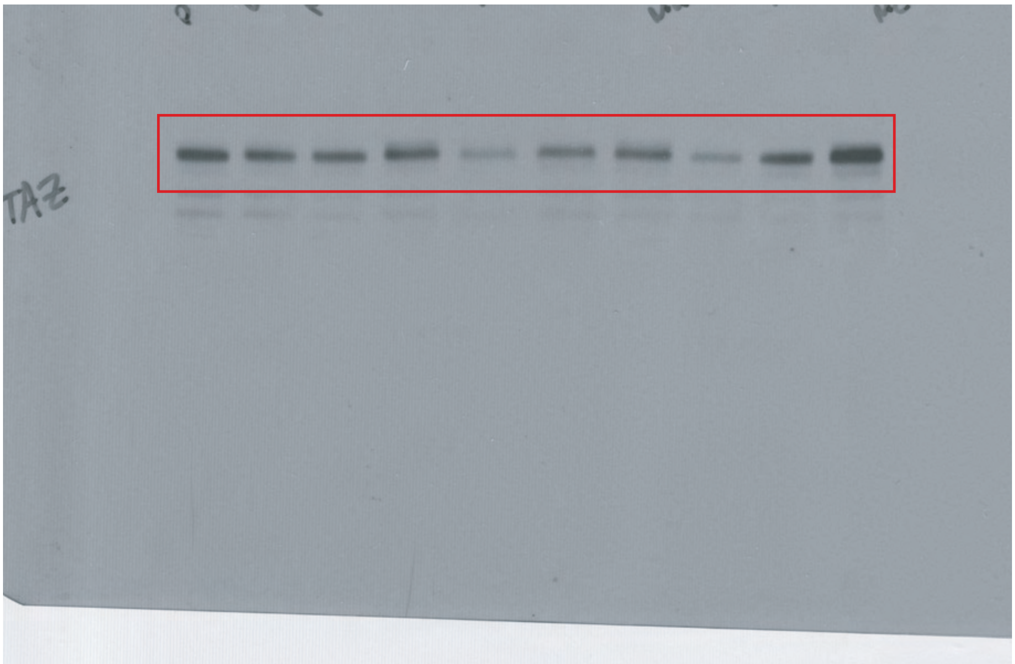

Figure 3E SJCRH30 IB: β-ACTIN

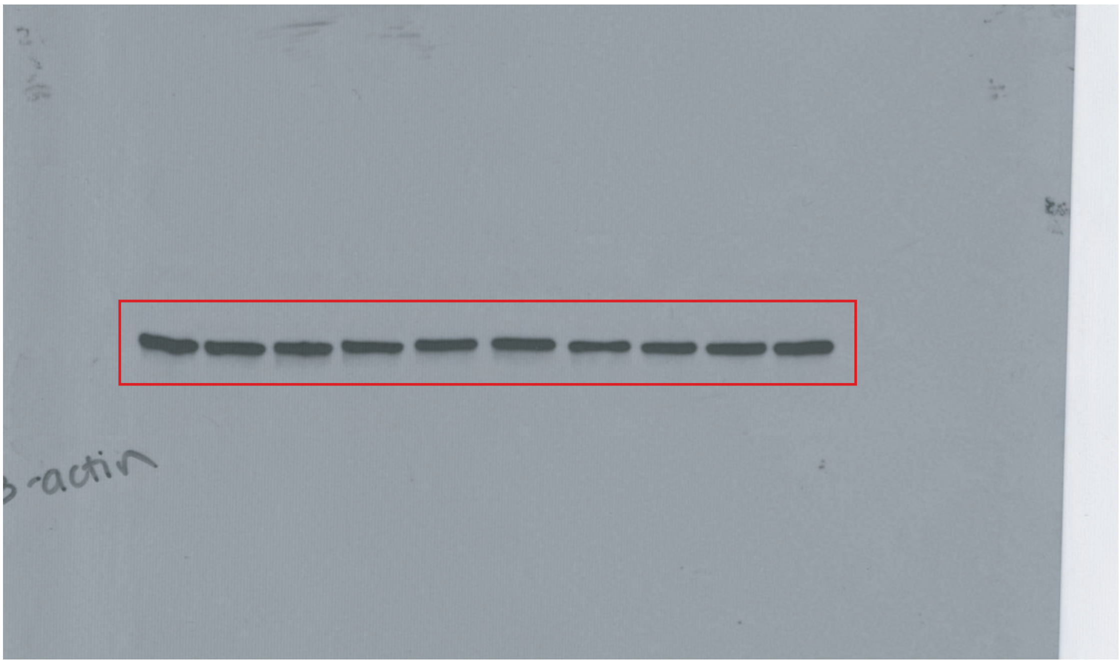

Full unedited gel for Figure 4

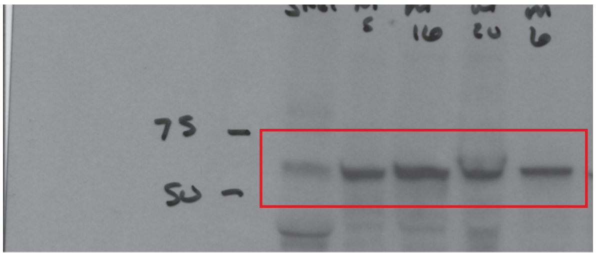

Figure 4A *Trp53*$^{fl/fl}$/*Pten*$^{fl/fl}$ MOUSE tumors IB: phospho-Akt (S437)

Figure 4A *Trp53*$^{fl/fl}$/*Pten*$^{fl/fl}$ MOUSE tumors IB: Total Akt

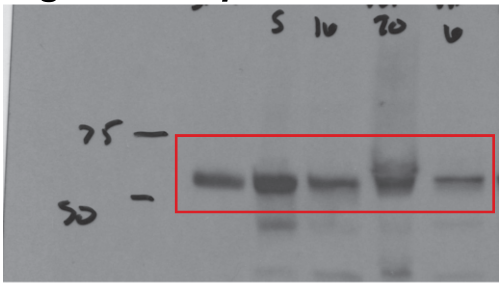

Figure 4A *Trp53*$^{fl/fl}$/*Pten*$^{fl/fl}$ MOUSE tumors IB: phospho-S6 (S235/236)

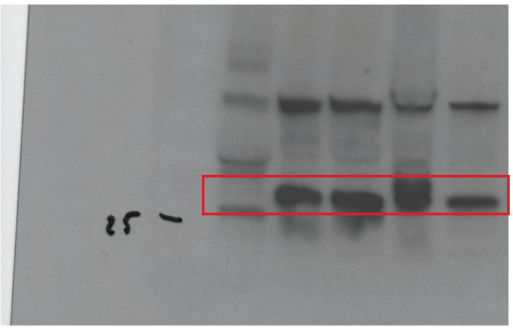

Figure 4A *Trp53*$^{fl/fl}$/*Pten*$^{fl/fl}$ MOUSE tumors IB: Total S6

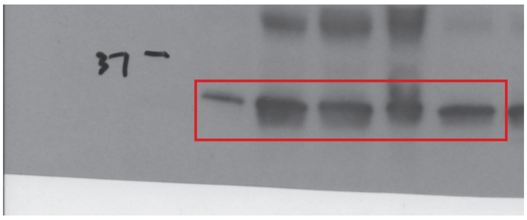

Figure 4A *Trp53*$^{fl/fl}$/*Pten*$^{fl/fl}$ MOUSE tumors IB: Taz

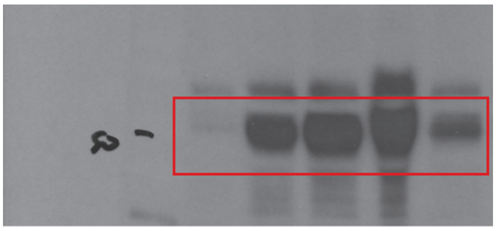

Figure 4A *Trp53*$^{fl/fl}$/*Pten*$^{fl/fl}$ MOUSE tumors IB: Yap

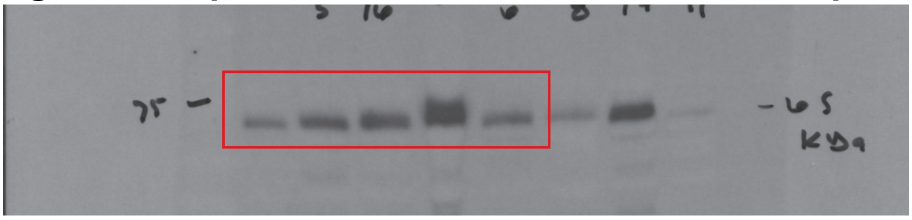

Figure 4A *Trp53*$^{fl/fl}$/*Pten*$^{fl/fl}$ MOUSE tumors IB: Vinculin

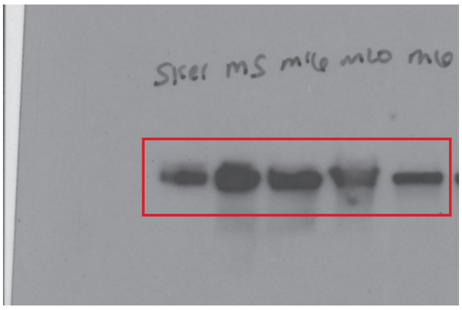

Full unedited gel for Figure 5B

A204

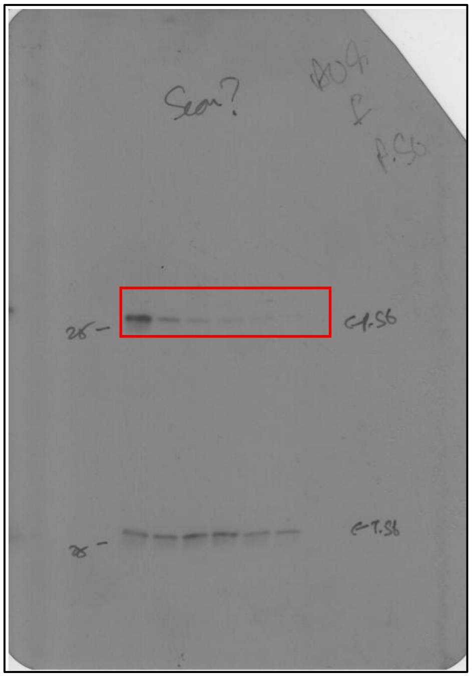

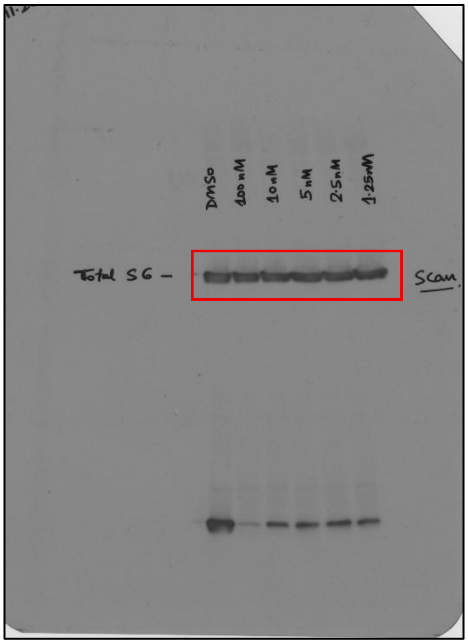

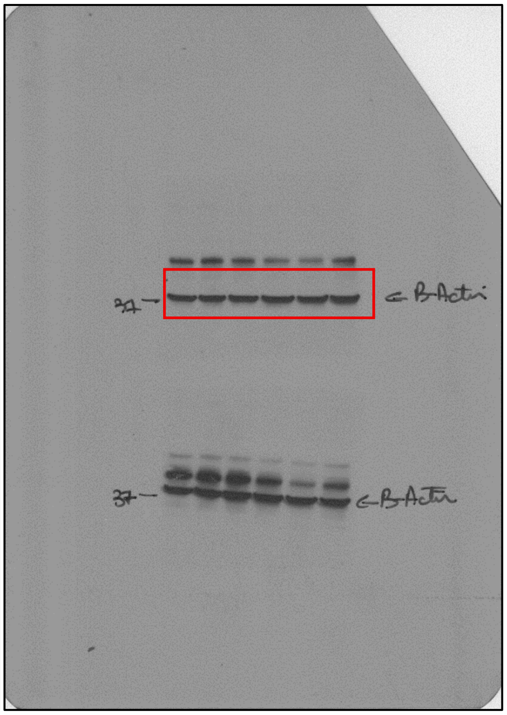

IB: Phosphorylated S6 (S235/236)

IB: Total S6

IB: β-Actin

SJCRH30

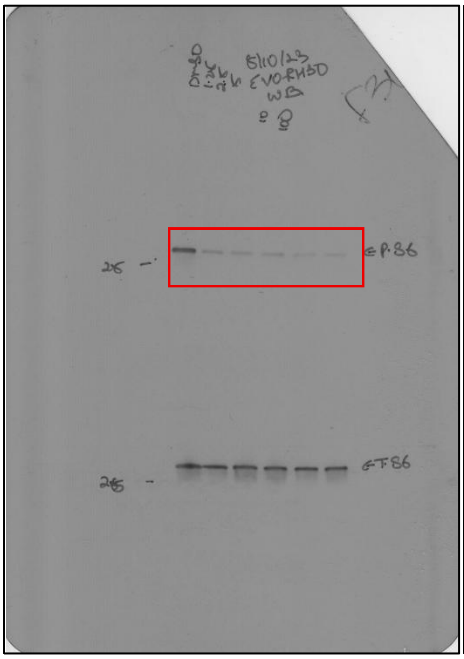

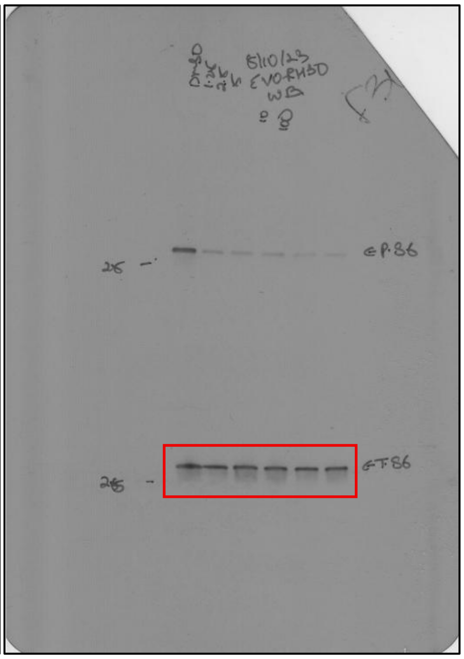

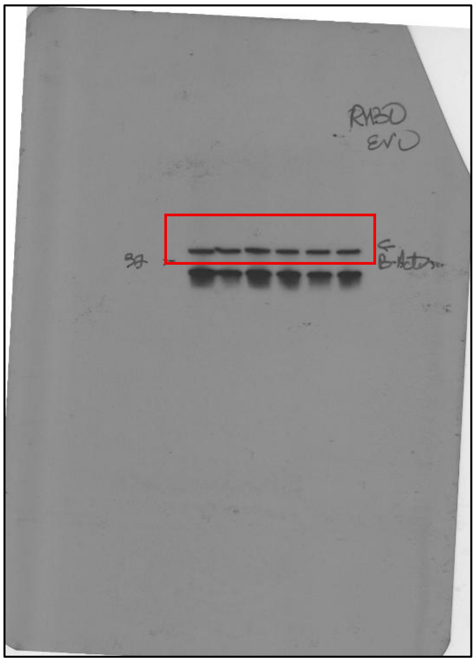

IB: Phosphorylated S6 (S235/236)

IB: Total S6

IB: β-Actin

Full unedited gel for Figure 5D

A204

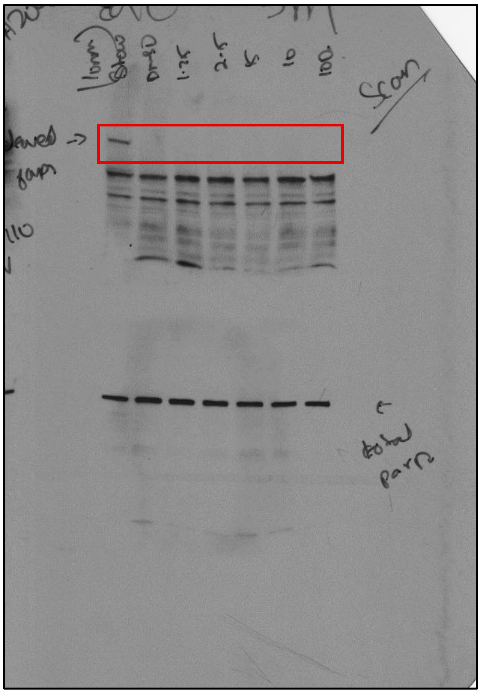

IB: Cleaved PARP (Asp214)

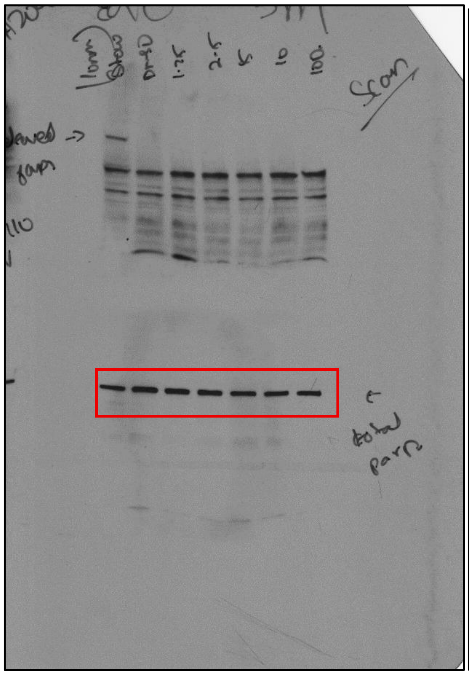

IB: Total PARP

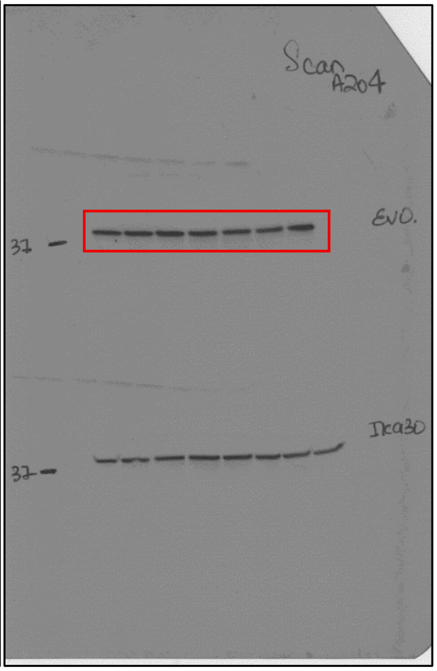

IB: β- Actin

SJCRH30

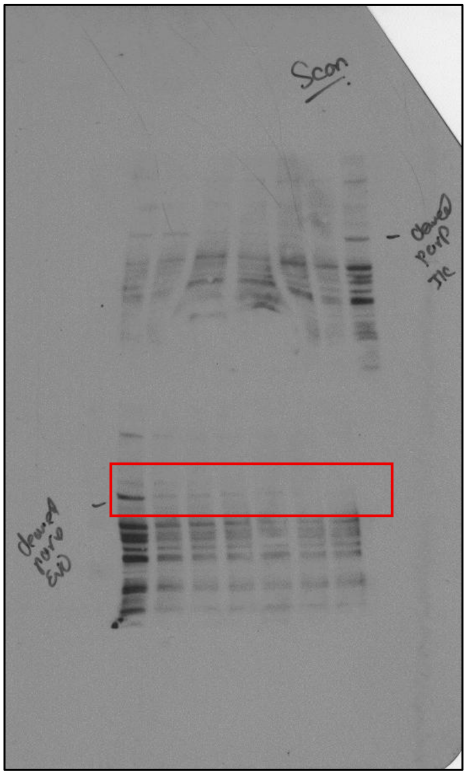

IB: Cleaved PARP (Asp214)

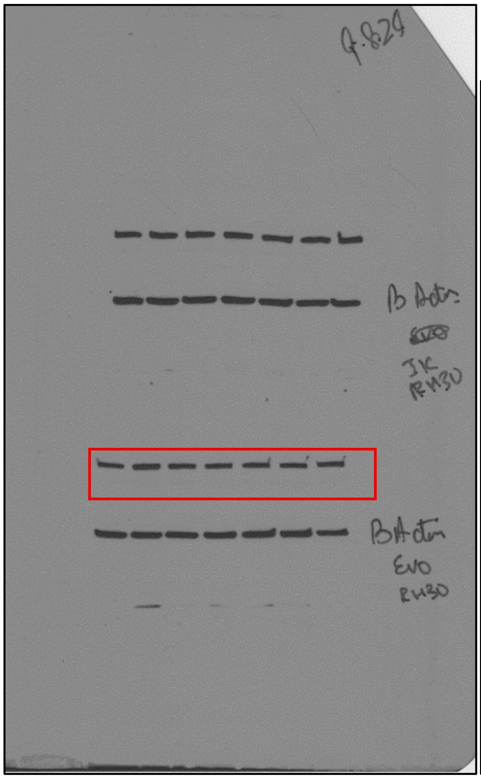

IB: Total PARP

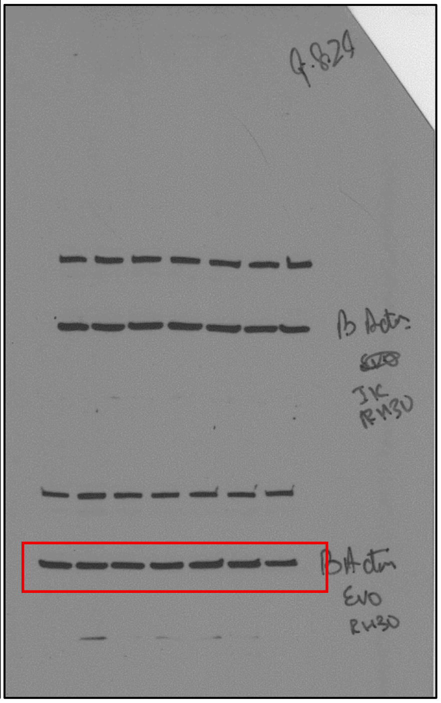

IB: β- Actin

Figure 6D

A204

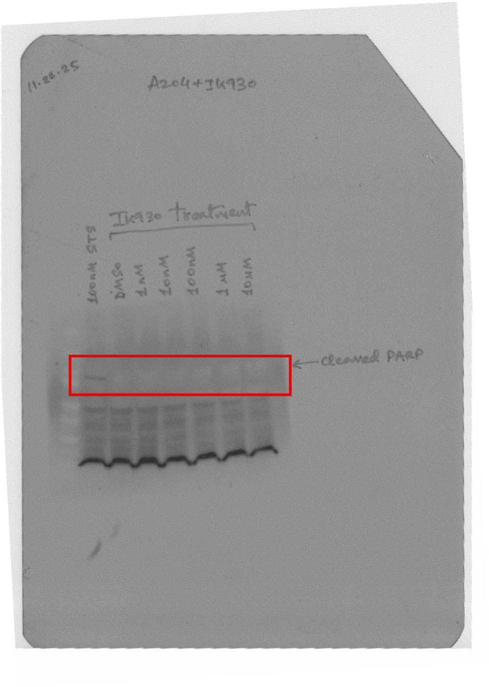

Cleaved PARP (Asp 214)

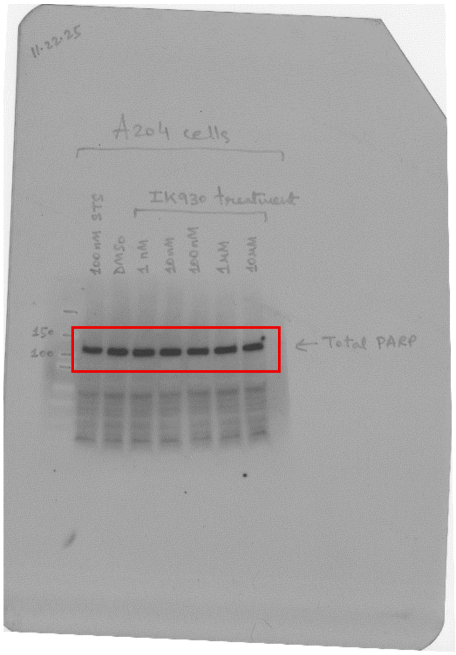

Total PARP

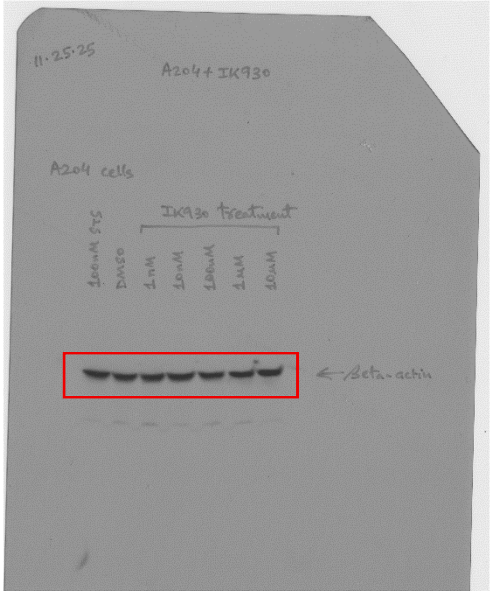

β- Actin

SJCRH30

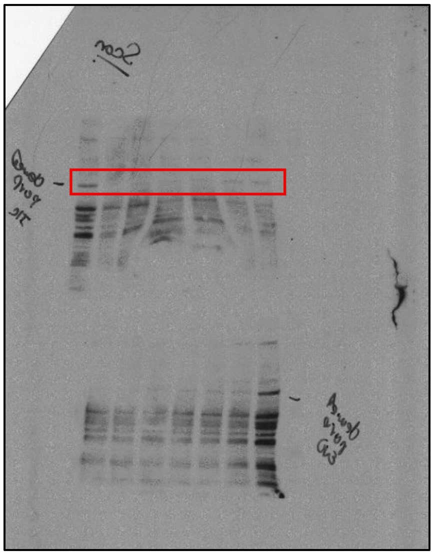

Cleaved PARP (Asp 214)

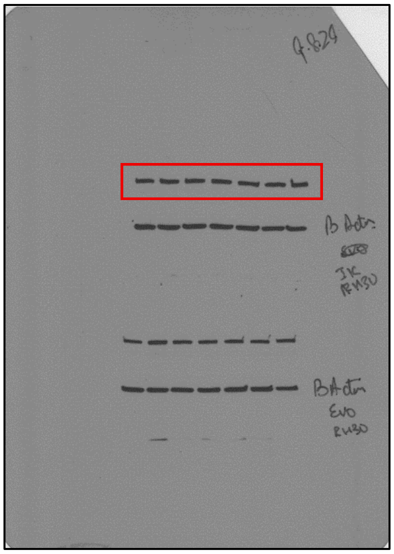

Total PARP

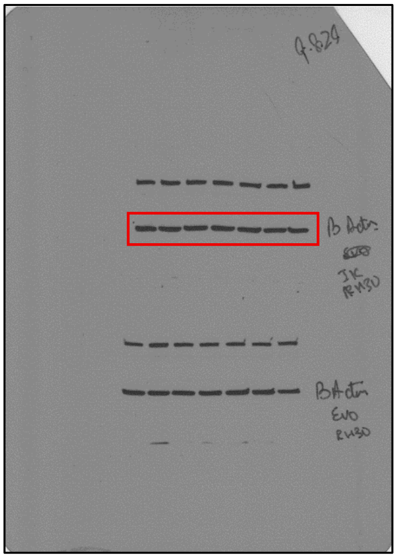

β- Actin

Full unedited gel for Figure 7C

A204

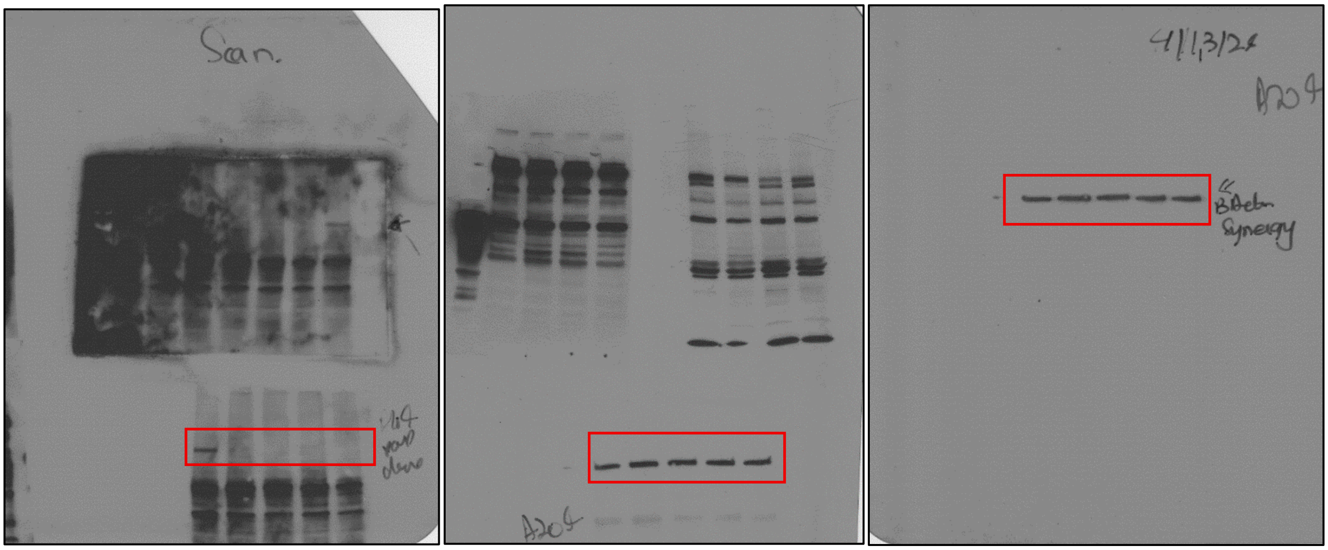

IB: Cleaved PARP (Asp214) IB: Total PARP IB: β- Actin

SJCRH30

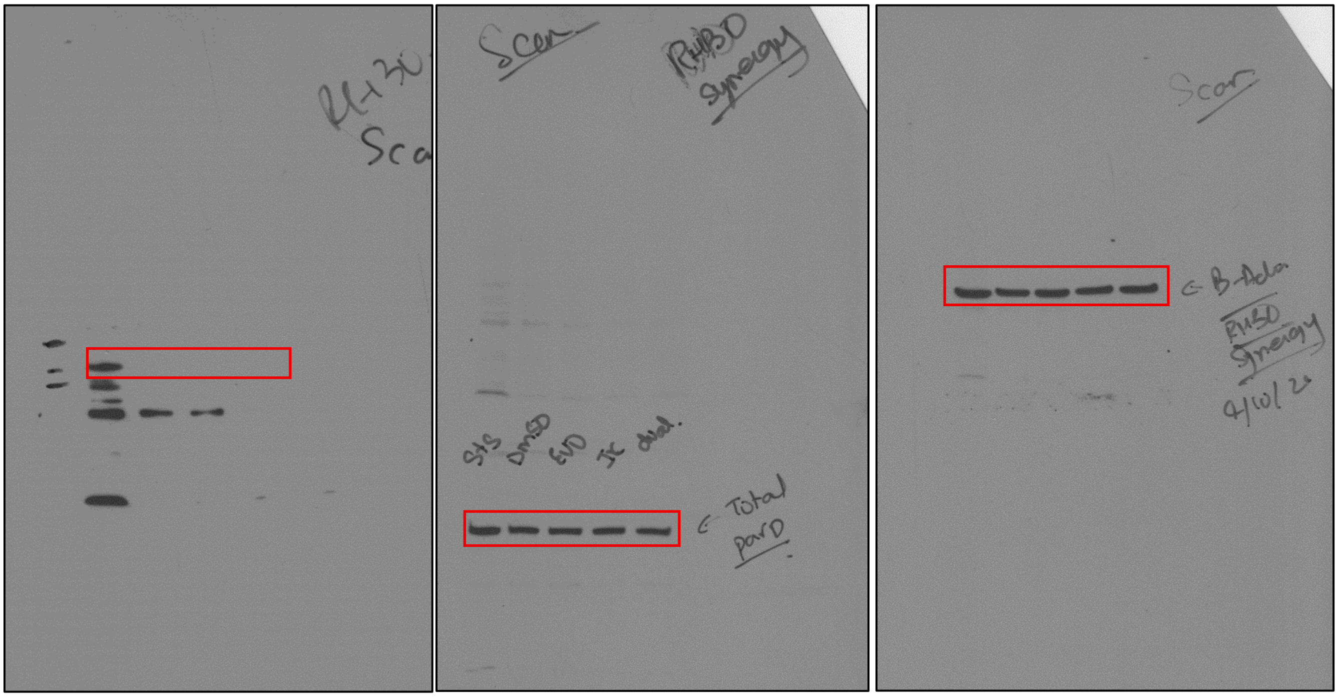

IB: Cleaved PARP (Asp214) IB: Total PARP IB: β- Actin

Full unedited gel for Figure S1D

Figure S1D SJCRH30 IB: phospho-AKT (S473)

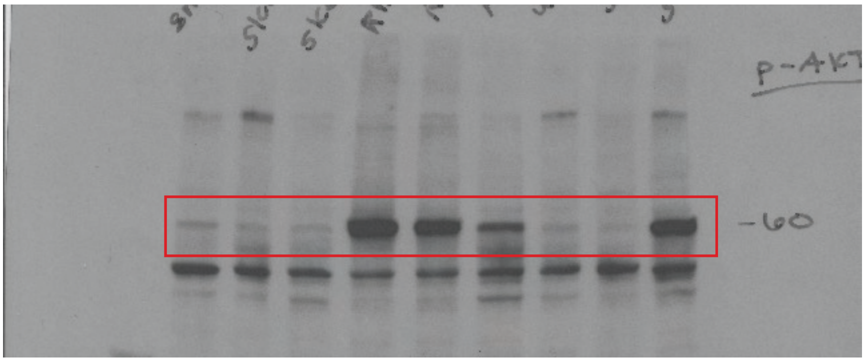

Figure S1D SJCRH30 IB: Total AKT

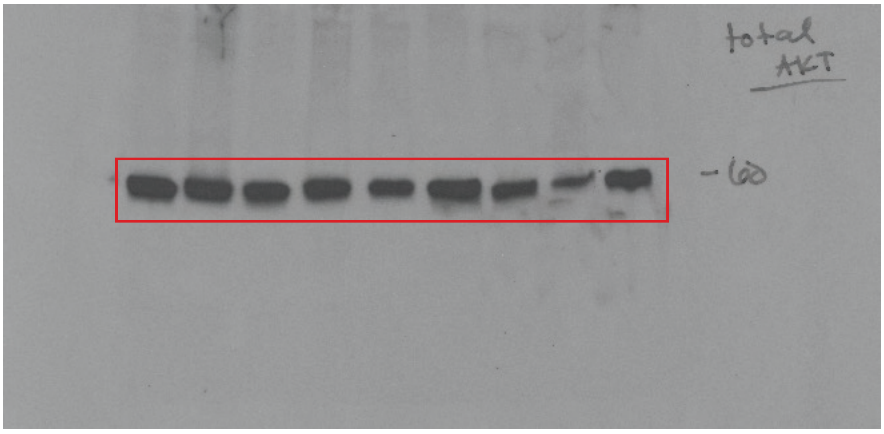

Figure S1D SJCRH30 IB: PTEN

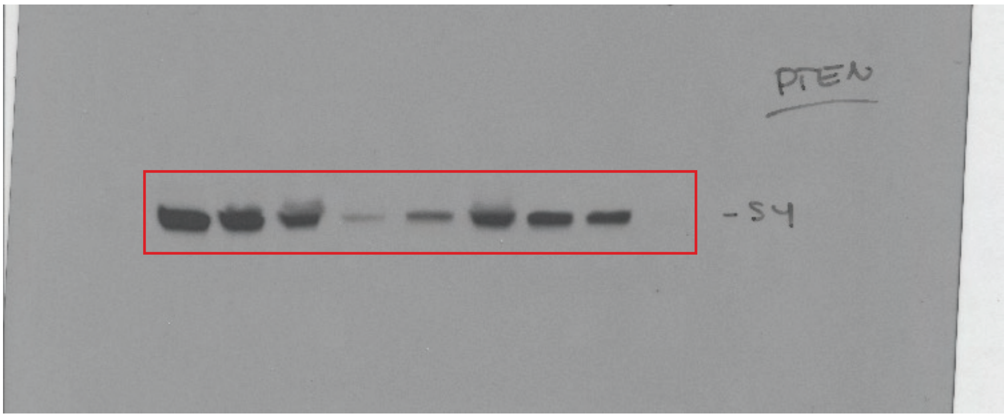

Figure S1D SJCRH30 IB: β-ACTIN

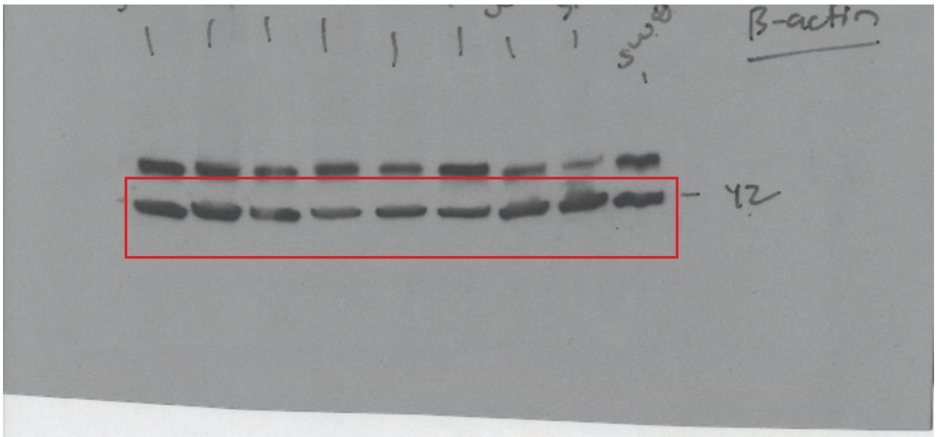

Full unedited gel for Figure S1I-J

Figure S1I SJCRH30 IB: TAZ

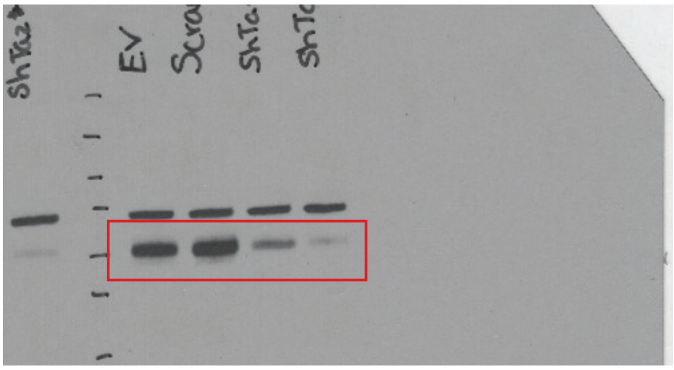

Figure S1I SJCRH30 IB: β-ACTIN (TAZ)

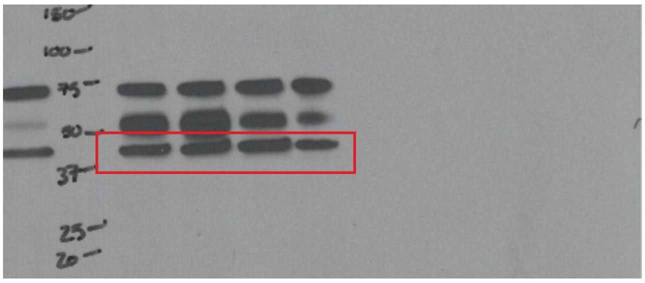

Figure S1I SJCRH30 IB: YAP

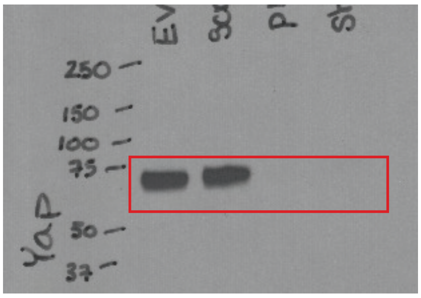

Figure S1I SJCRH30 IB: β-ACTIN (YAP)

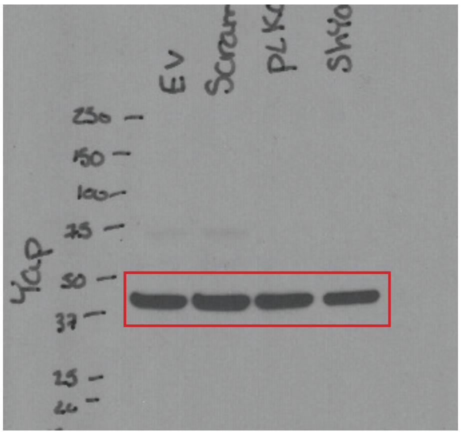

Figure S1J A204 IB: TAZ

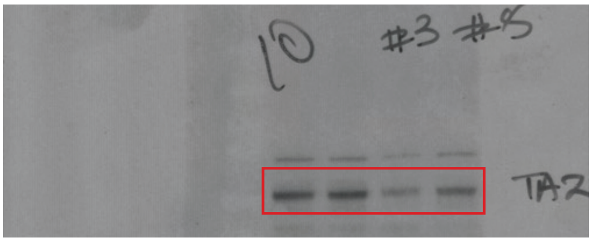

Figure S1J A204 IB: β-ACTIN (TAZ)

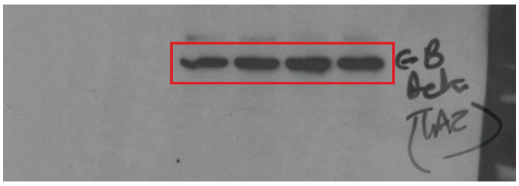

Figure S1J A204 IB: YAP

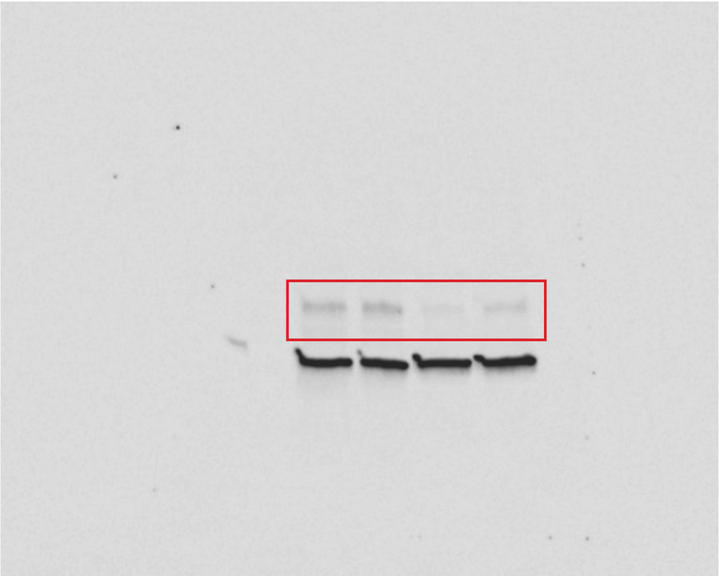

Figure S1J A204 IB: β-ACTIN (YAP)

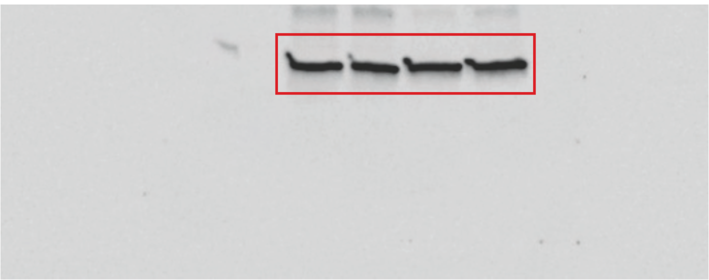

Full unedited gel for Figure S2A-C

Figure S2A SJCRH30 IB: TAZ

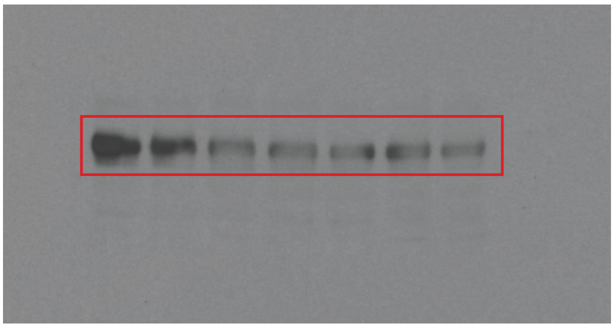

Figure S2A SJCRH30 IB: β-ACTIN

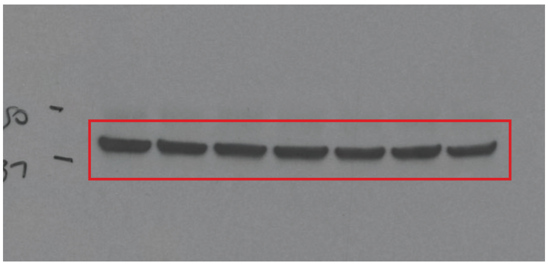

Figure S2C SJCRH30 IB: PDK1

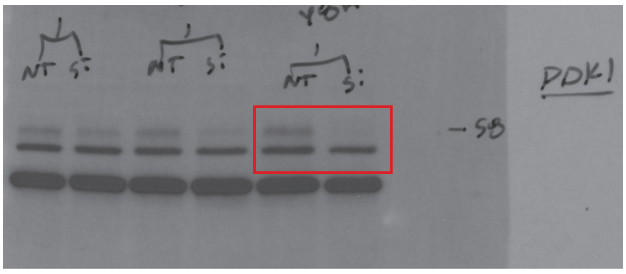

Figure S2C SJCRH30 IB: β-ACTIN

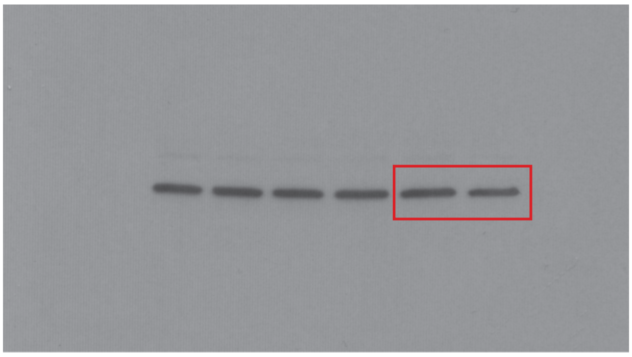

Figure S2B A204 IB: TAZ

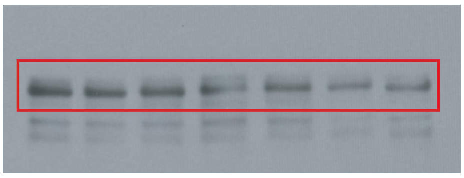

Figure S2B A204 IB: β-ACTIN

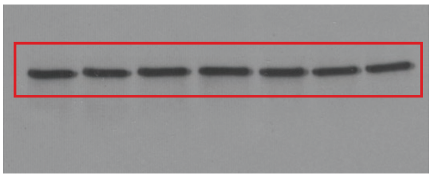

Figure S2C SJCRH30 IB: YAP

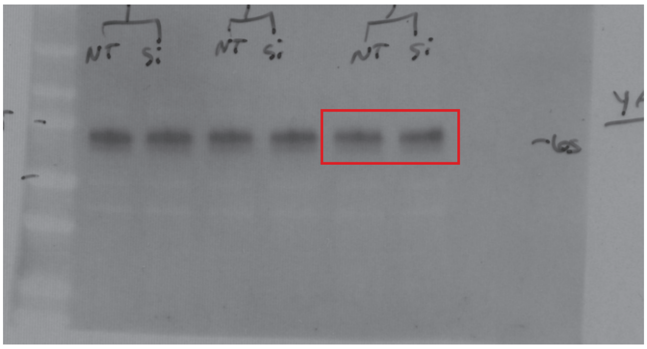

Figure S2C SJCRH30 IB: TAZ

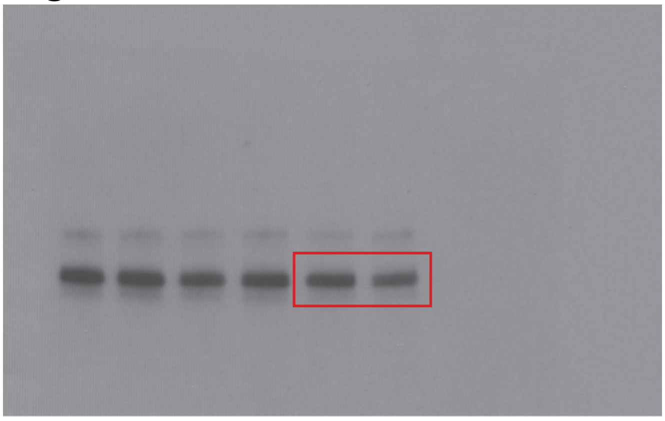

Figure S2C A204 IB: TAZ

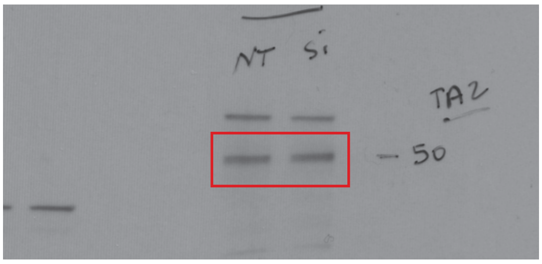

Figure S2C A204 IB: PDK1

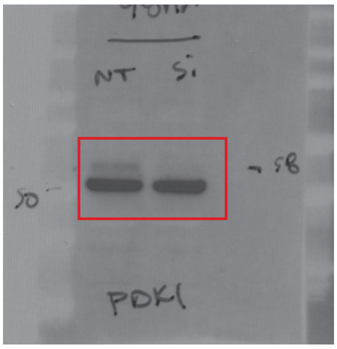

Figure S2C A204 IB: β-ACTIN

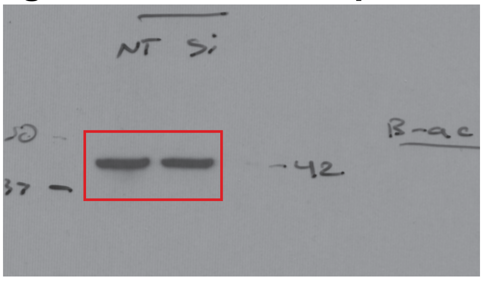

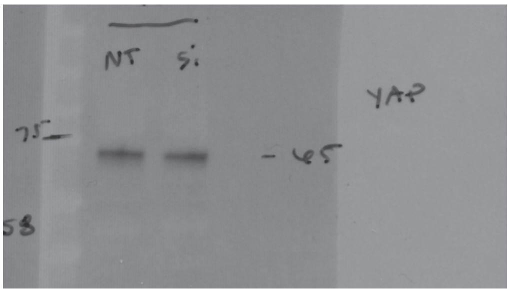

Figure S2C A204 IB: YAP

Full unedited gel for Figure S2D

Figure S2D A204 IB: AKT

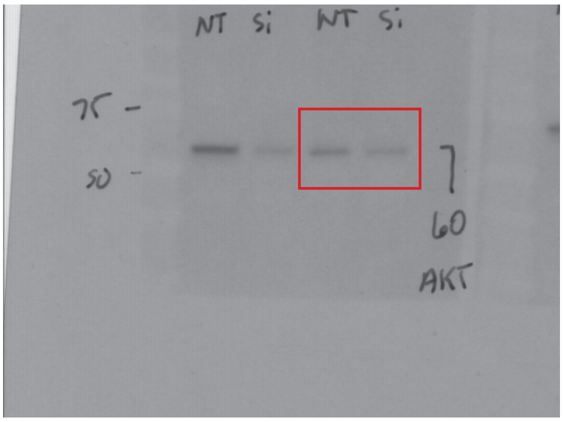

Figure S2D A204 IB: β-ACTIN

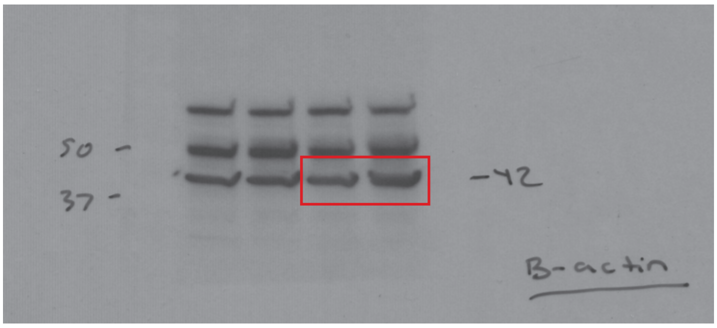

Figure S2D A204 IB: TAZ

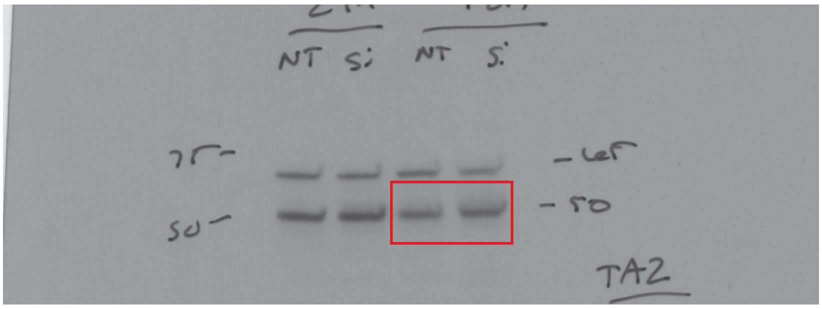

Figure S2D A204 IB: YAP

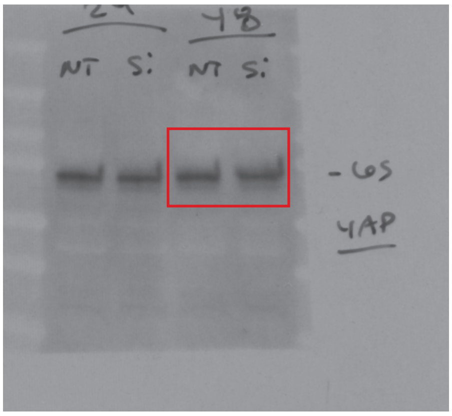

Figure S2D SJCRH30 IB: AKT

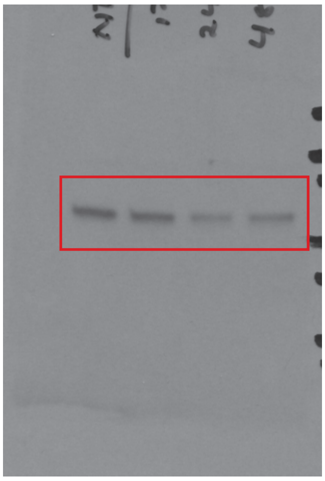

Figure S2D SJCRH30 IB: TAZ

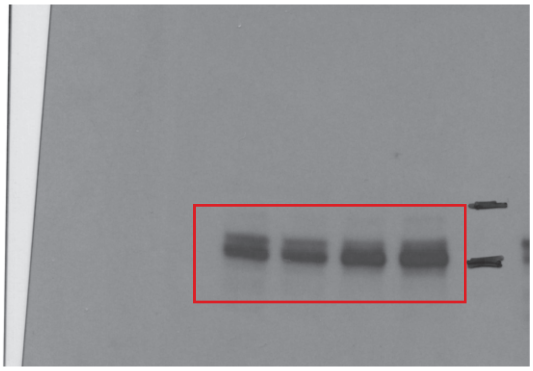

Figure S2D SJCRH30 IB: YAP

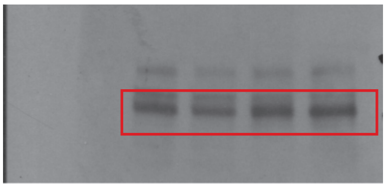

Figure S2D SJCRH30 IB: β-ACTIN

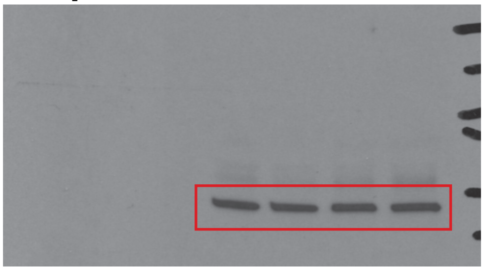

Full unedited gel for Figure S2E

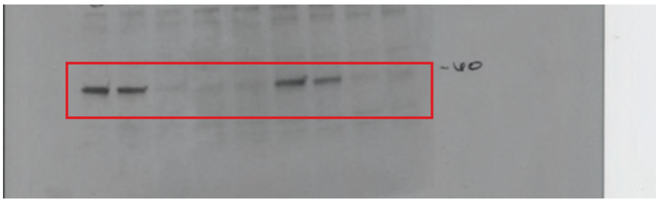

Figure S2E SJCRH30 IB: phospho-AKT (S473)

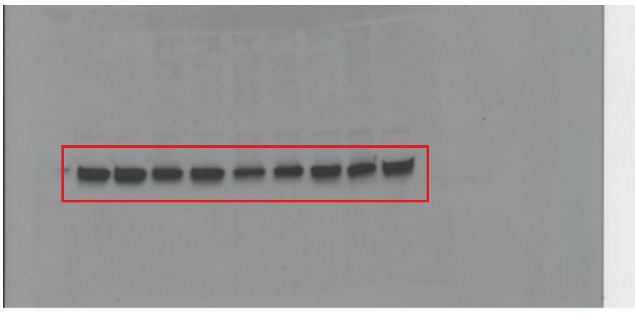

Figure S2E SJCRH30 IB: Total AKT

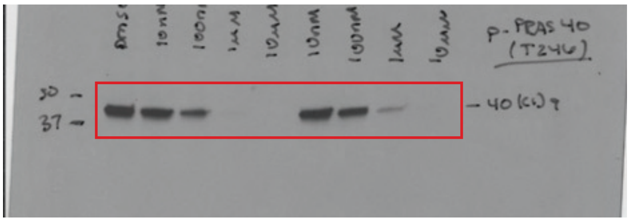

Figure S2E SJCRH30 IB: phospho-PRAS40 (T246)

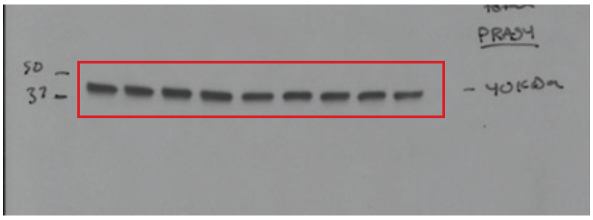

Figure S2E SJCRH30 IB: Total PRAS40

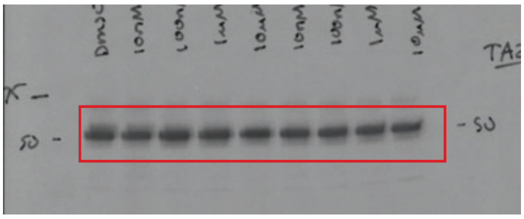

Figure S2E SJCRH30 IB: TAZ

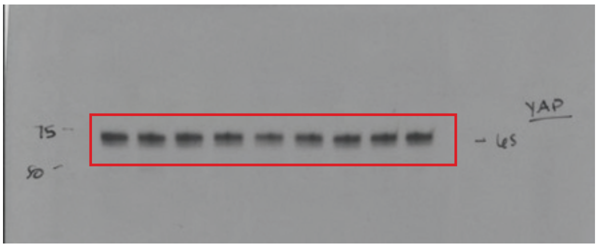

Figure S2E SJCRH30 IB: YAP

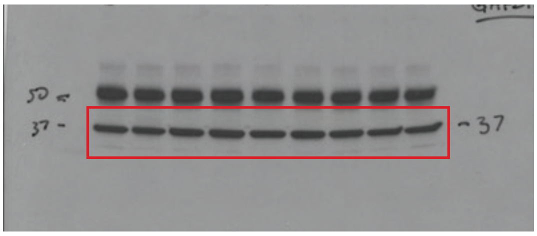

Figure S2E SJCRH30 IB: β-ACTIN

Full unedited gel for Figure S2F

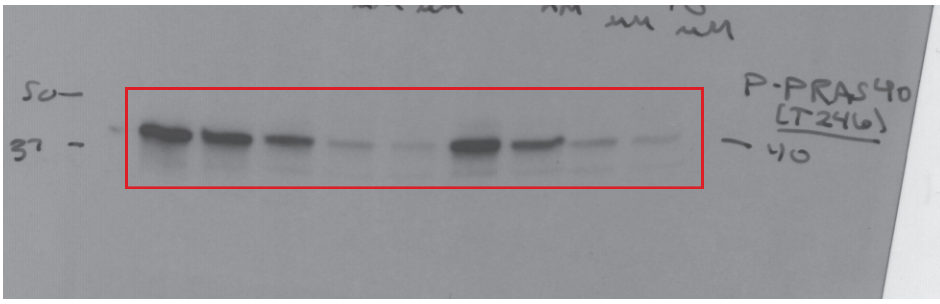

Figure S2F A204
IB: phospho-PRAS40 (T246)

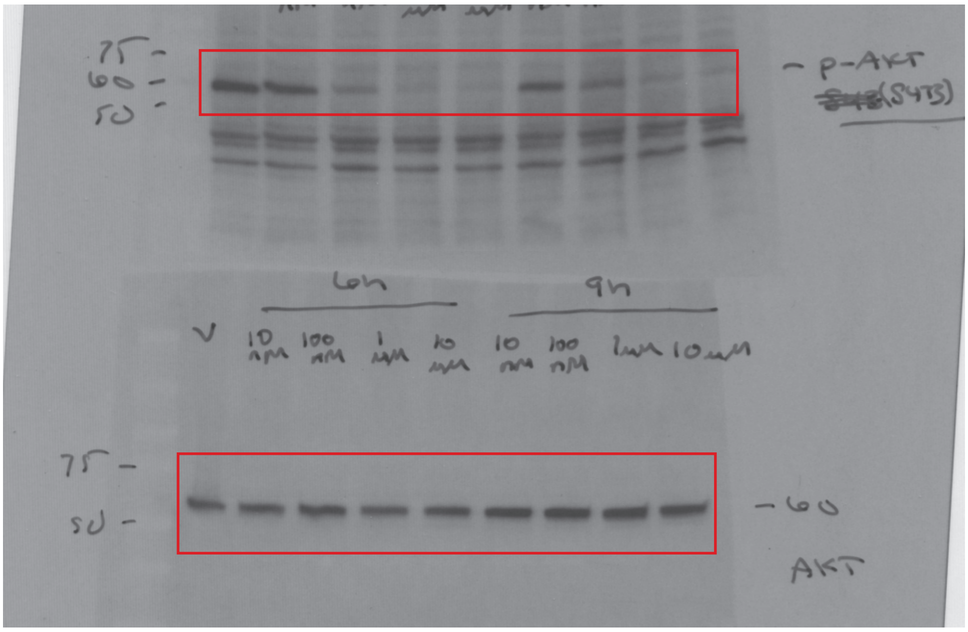

Figure S2F A204
IB: phospho-AKT (S473)

Figure S2F A204 IB: Total AKT

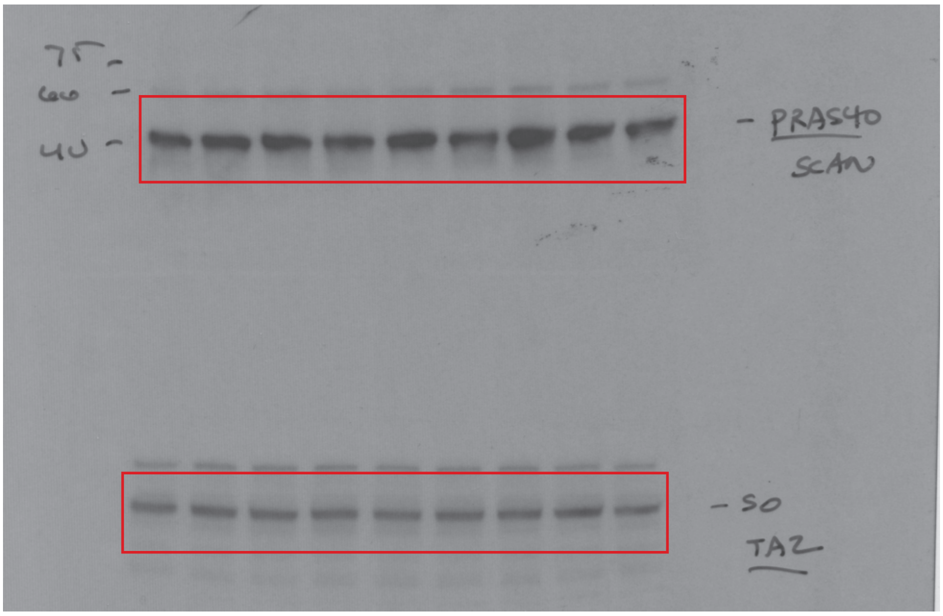

Figure S2F A204 IB: Total PRAS40

Figure S2F A204 IB: TAZ

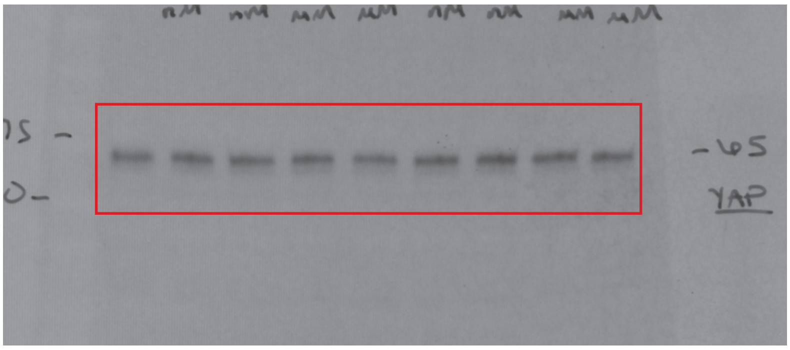

Figure S2F A204 IB: YAP

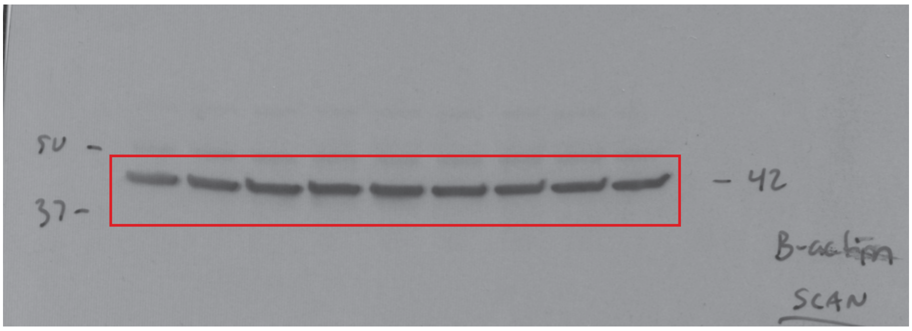

Figure S2F A204 IB: β-ACTIN

Full unedited gel for Figure S3B

Figure S3B SJCRH30 IB: Flag-TAZ and Flag-TAZ(S58/62A)

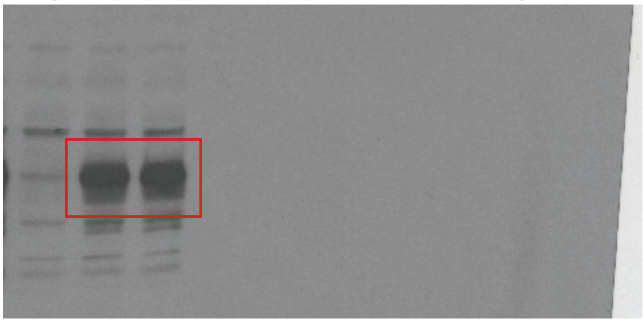

Figure S3B SJCRH30 IB: β-ACTIN

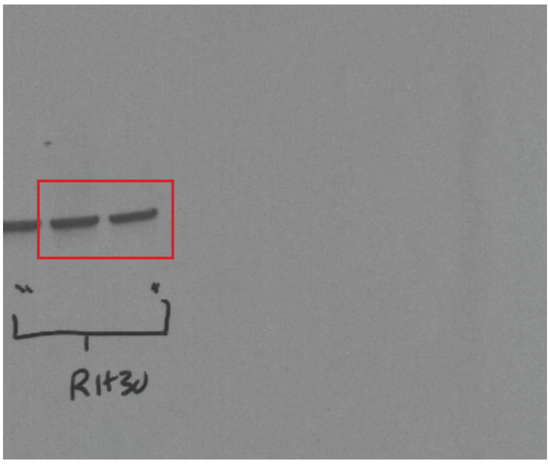

Figure S3B A204 IB: Flag-TAZ and Flag-TAZ(S58/62A)

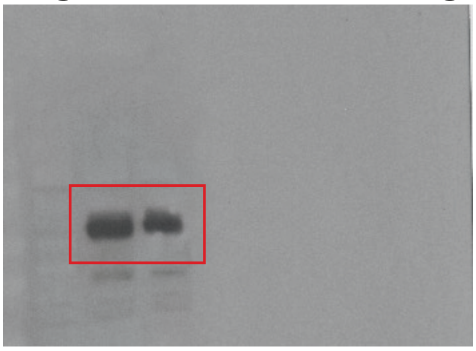

Figure S3B A204 IB: GAPDH

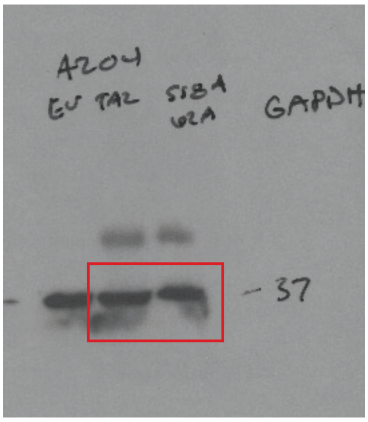

Full unedited gel for Figure S3C

Figure S3C SJCRH30 IB: phospho-AKT (S473)

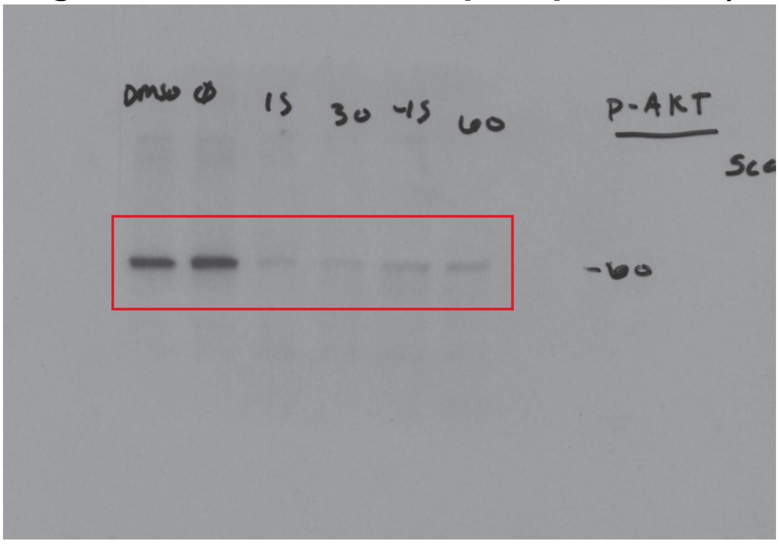

Figure S3C SJCRH30 IB: Total AKT

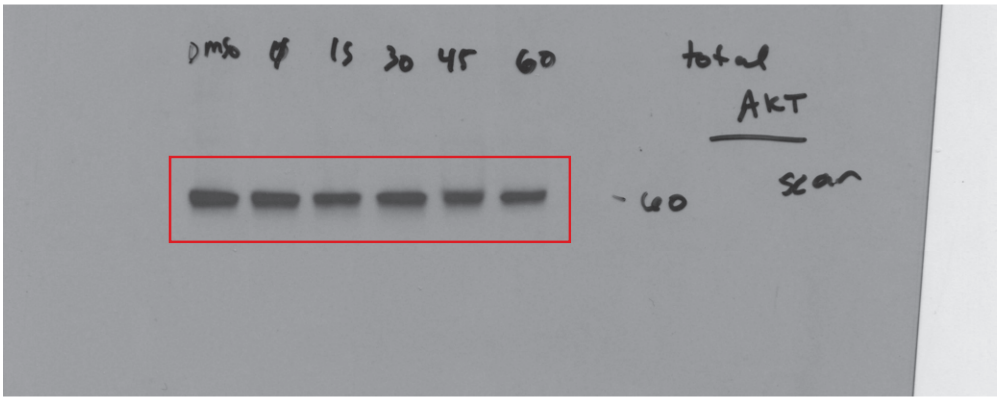

Figure S3C SJCHR30 IB: Flag-TAZ

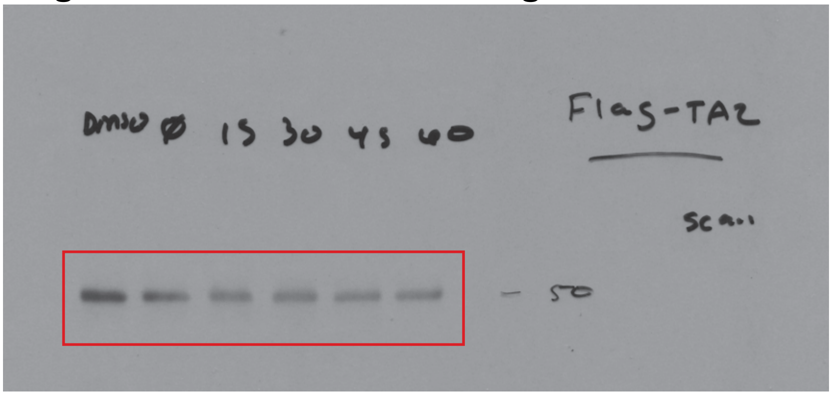

Figure S3C SJCRH30 IB: GAPDH

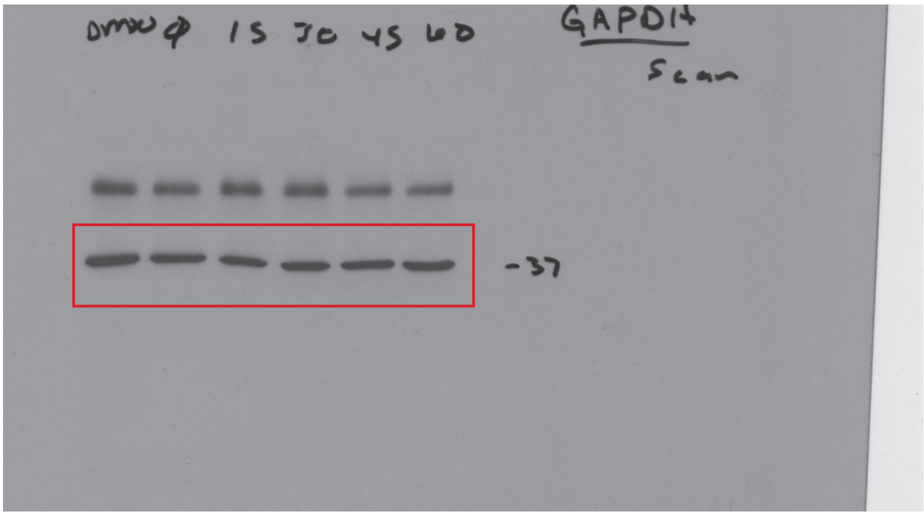

Full unedited gel for Figure S3D

Figure S3D SJCRH30 IB: phospho-AKT (S473) (top)
Figure S3D SJCRH30 IB: Total AKT (bottom)

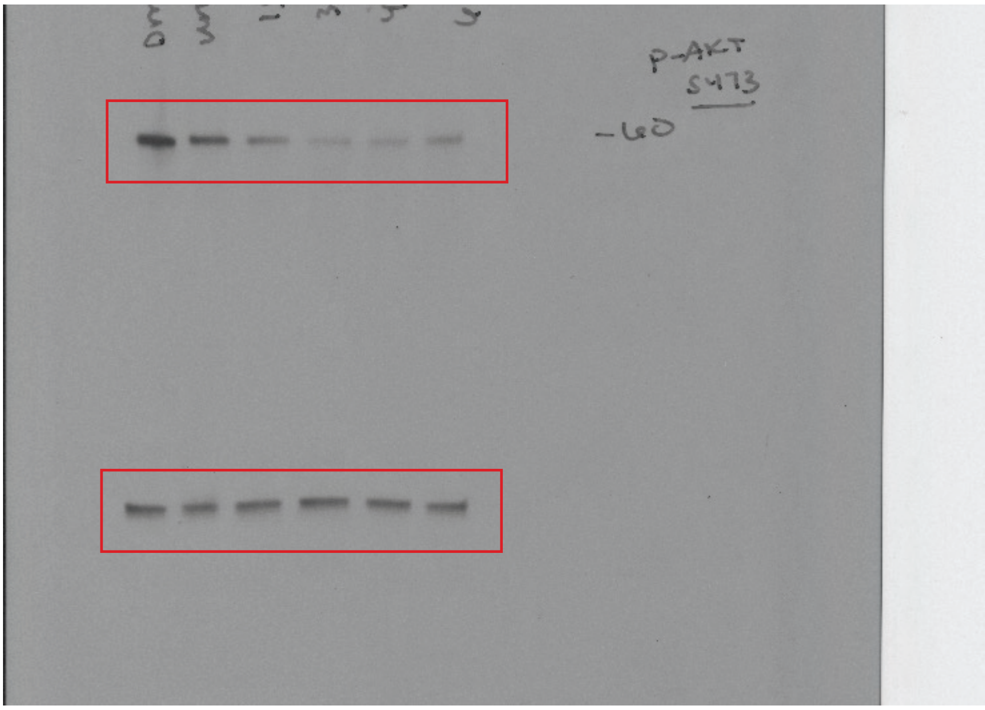

Figure S3D SJCHR30 IB: Flag-TAZ (S58/62A)

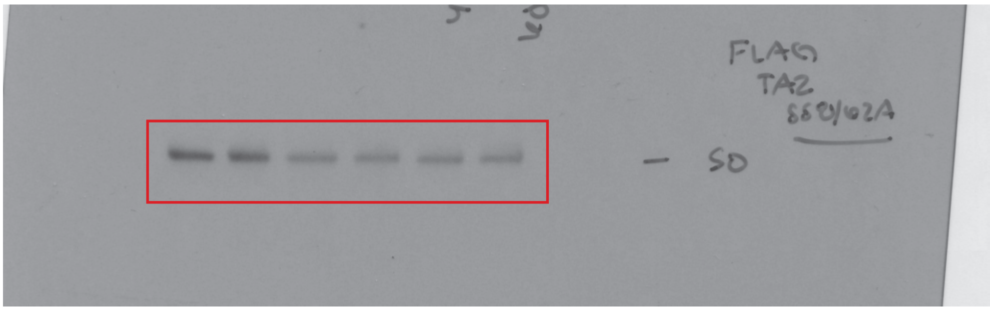

Figure S3D SJCRH30 IB: GAPDH

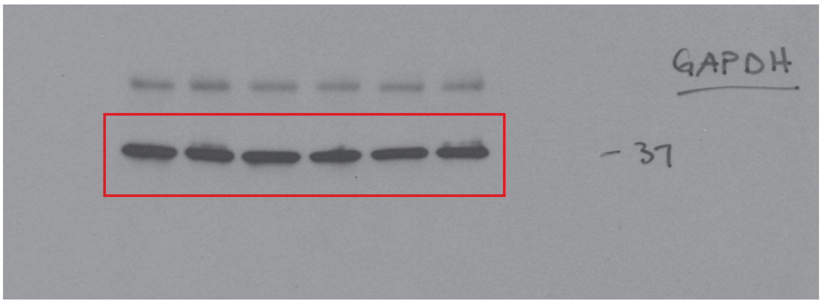

Full unedited gel for Figure S3E-F

Figure S3E A204 IB: Flag-TAZ

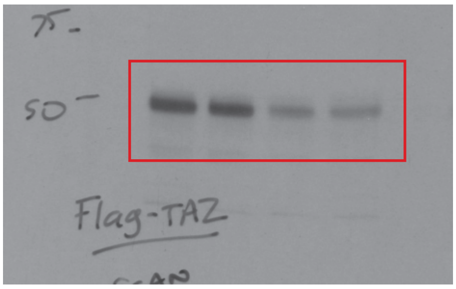

Figure S3F A204 IB: Flag-TAZ (S58/62A)

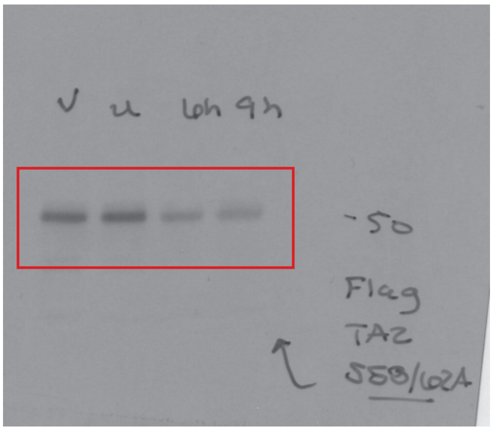

Figure S3E A204 IB: GAPDH (left) and Figure S3F A204 IB: GAPDH (right)

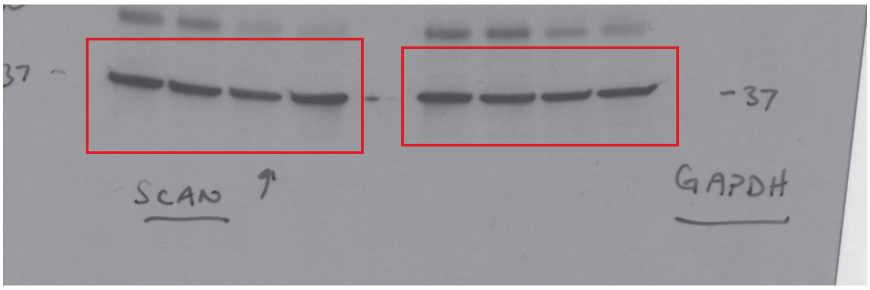

Figure S3E A204 IB: Total AKT (left) and Figure S3F A204 IB: Total AKT (right)

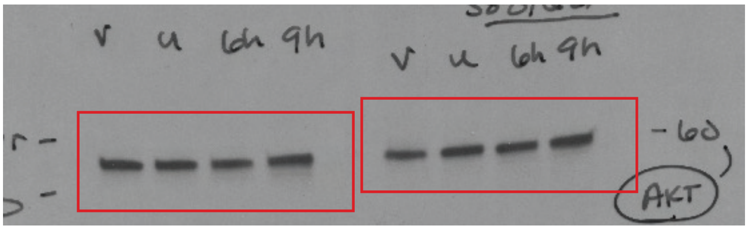

Figure S3E A204 IB: phospho-AKT (S473) (left) and Figure S3F A204 IB: phospho-AKT (S473) (right)

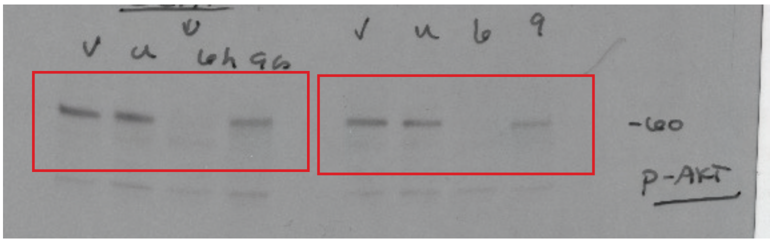

Full unedited gel for Figure S4A-B

Figure S4A SJCRH30 IB: phospho-AKT (S473) and Total AKT

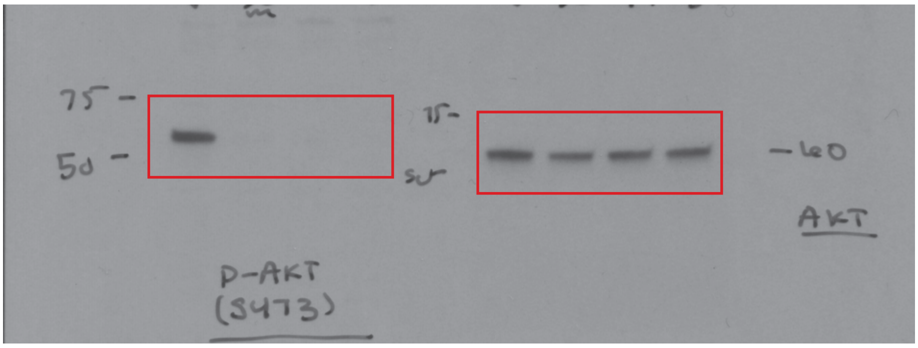

Figure S4A SJCRH30 IB: phospho-TAZ(S89) (bottom) and phospho-YAP (S127) (top)

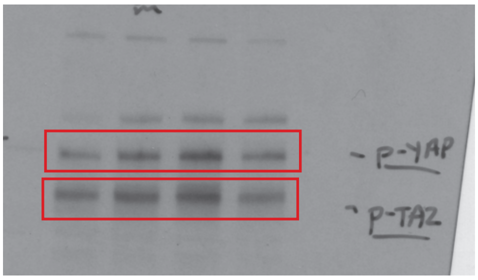

Figure S4A SJCRH30 IB: YAP

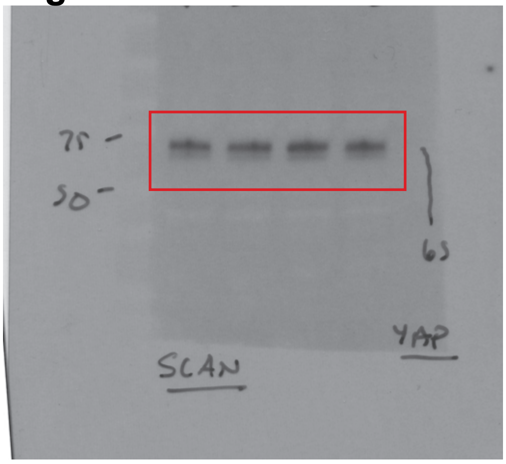

Figure S4A SJCRH30 IB: TAZ

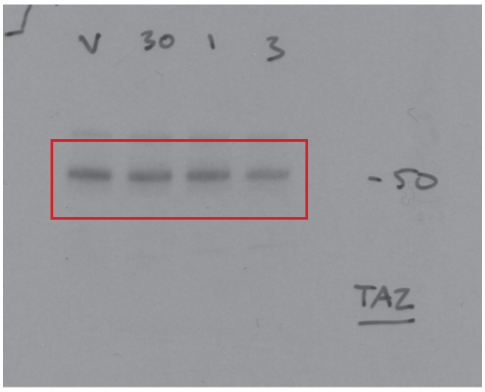

Figure S4A SJCRH30 IB: β-ACTIN

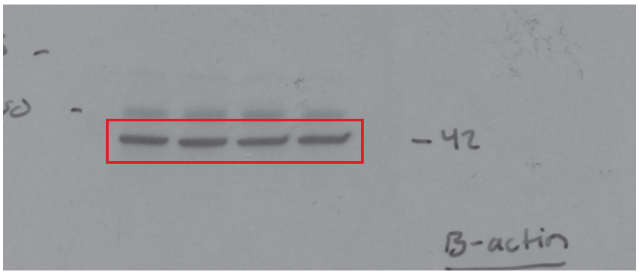

Figure S4B A204 IB: phospho-AKT (S473) and Total AKT

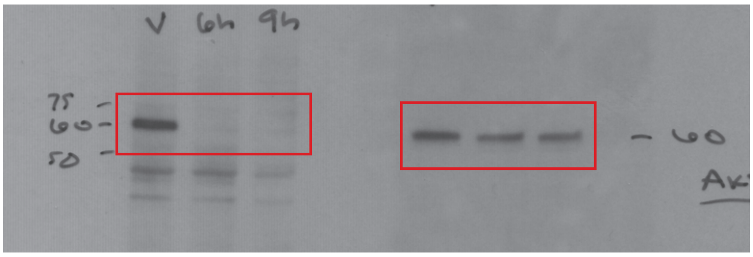

Figure S4B A204 IB: YAP

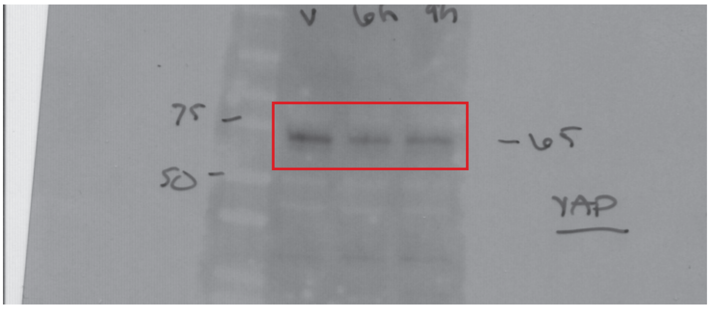

Figure S4B A204 IB: TAZ

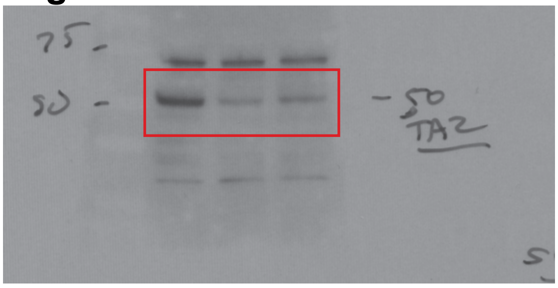

Figure S4B A204 IB: β-ACTIN

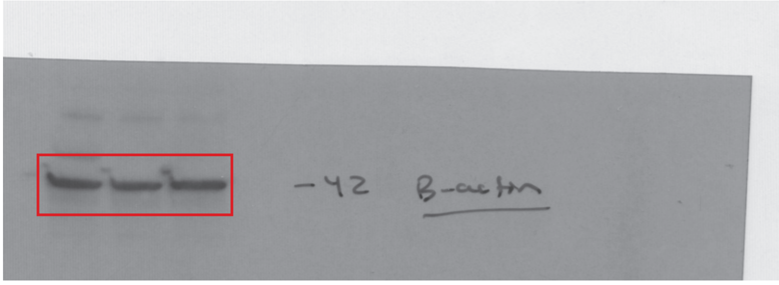

Full unedited gel for Figure S4C-D

Figure S4C SJCHR30 IB: Flag-TAZ

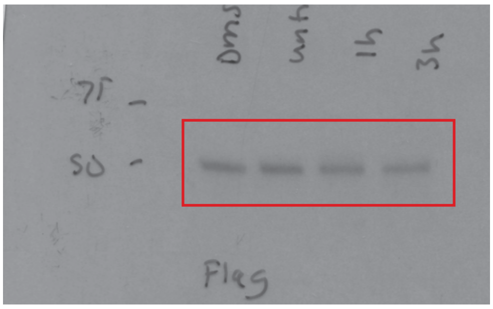

Figure S4C SJCRH30 IB: β-ACTIN

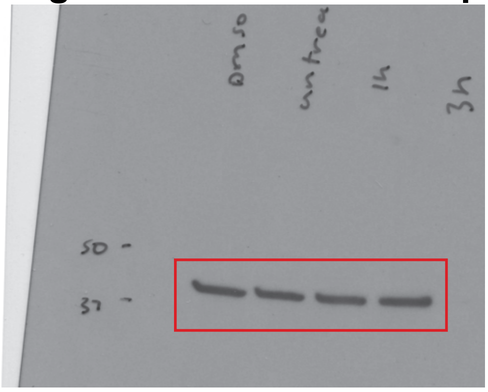

Figure S4D SJCHR30 IB: Flag-TAZ (4SA)

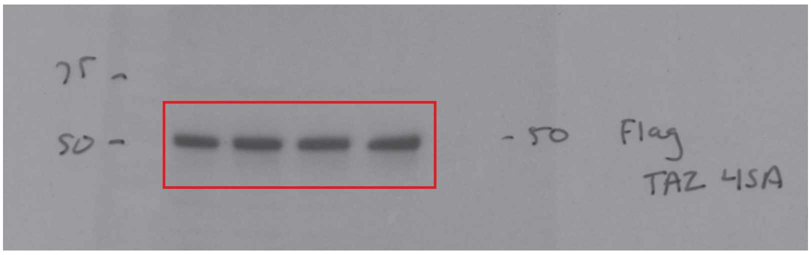

Figure S4D SJCRH30 IB: β-ACTIN

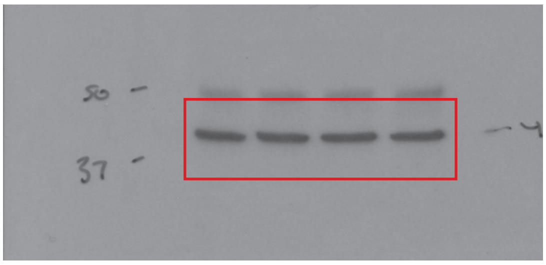

Figure 2F

Gel 1

Slide 1

IB: YAP 65 kDa, cytoplasmic, nuclear fractions

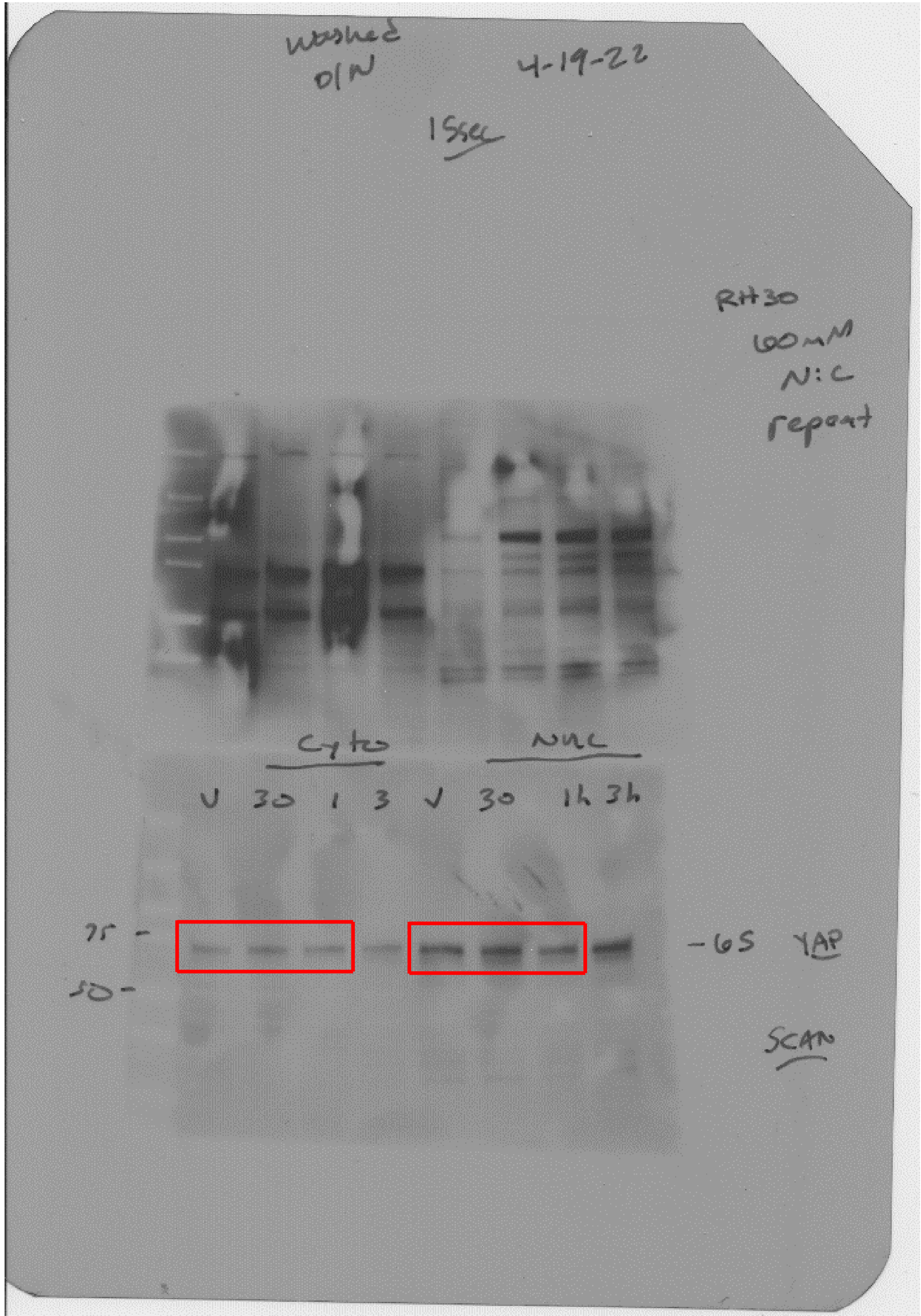

Figure 2F

Gel 1

Slide 2

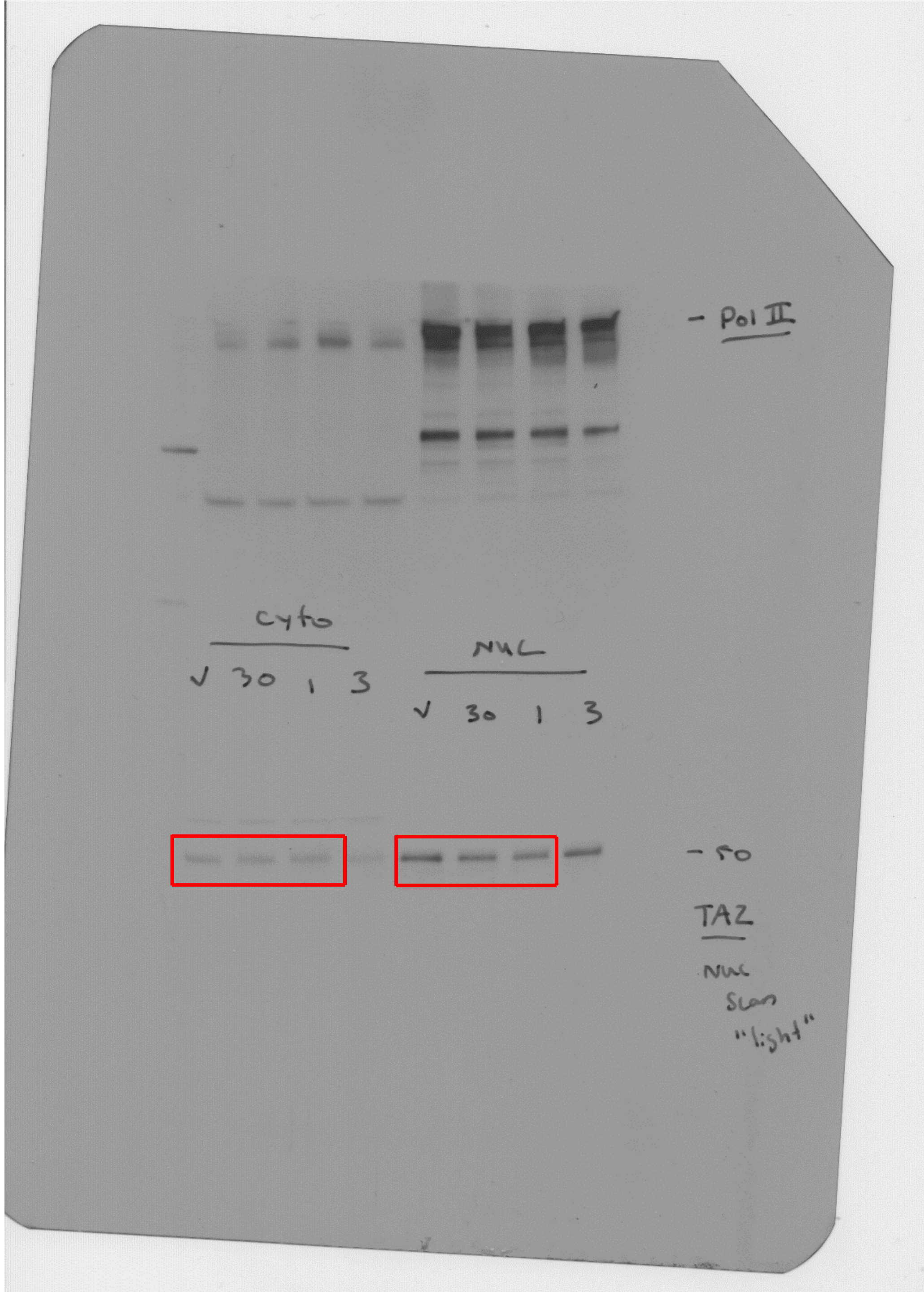

IB: TAZ 50 kDa, cytoplasmic, nuclear fractions

Figure 2F

Gel 2

Slide 3

Top band (phospho-YAP), 65 kDa

Lower band (phospho-TAZ), 50 kDa

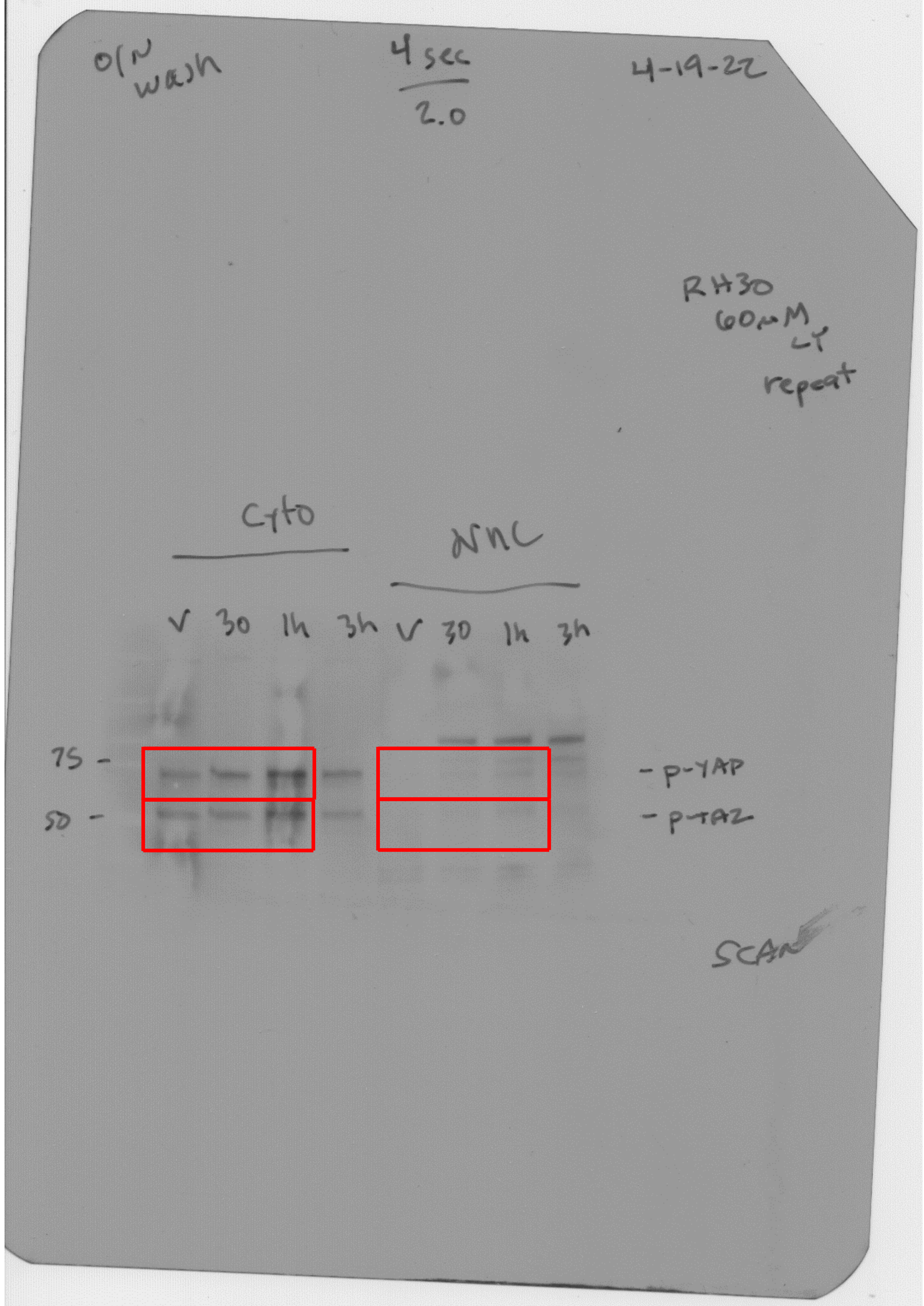

Figure 2F

Gel 3

Slide 4

IB: α – tubulin 50 kDa

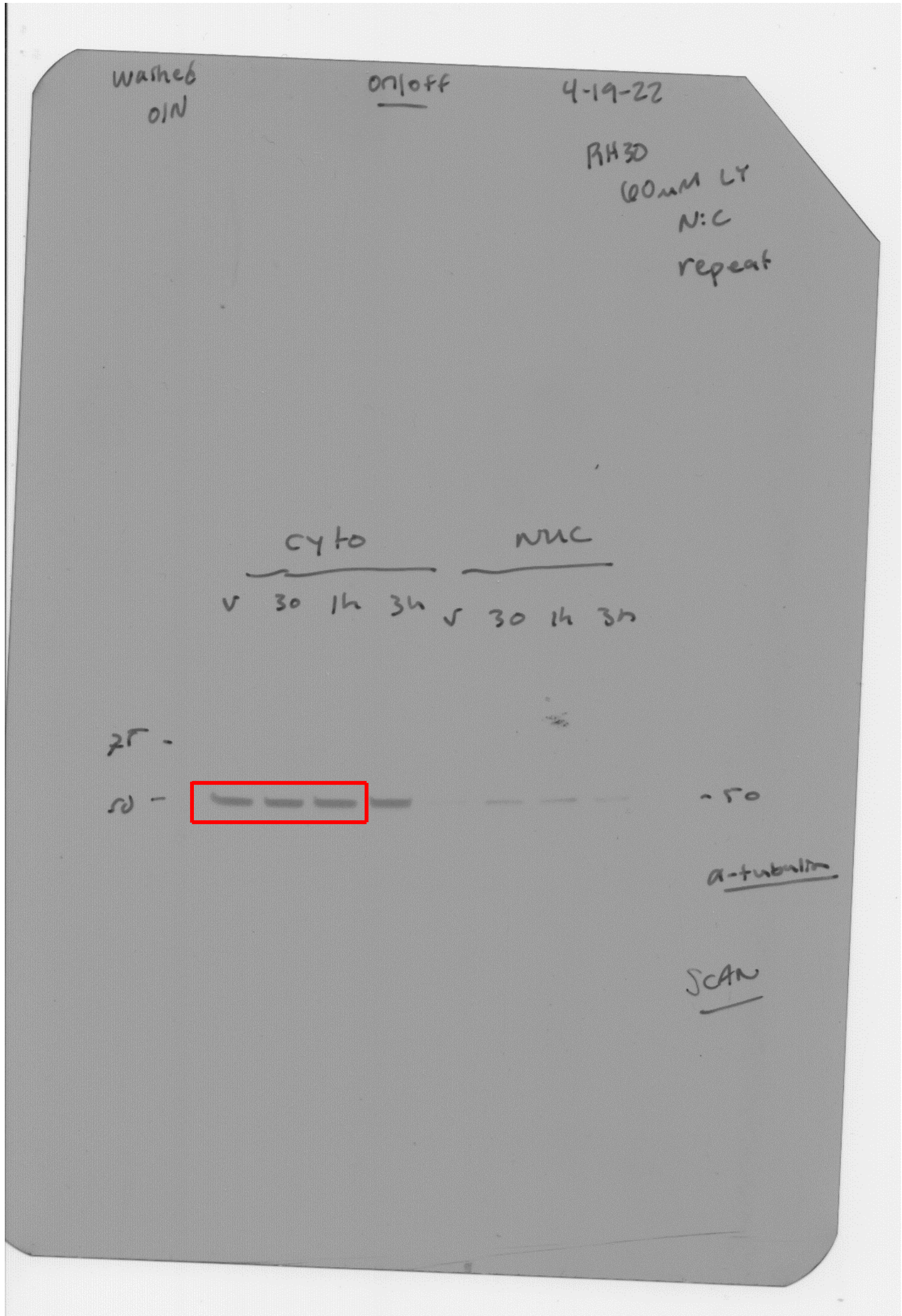

Gel 3

Slide 5

IB: RNA Pol II 250 kDa

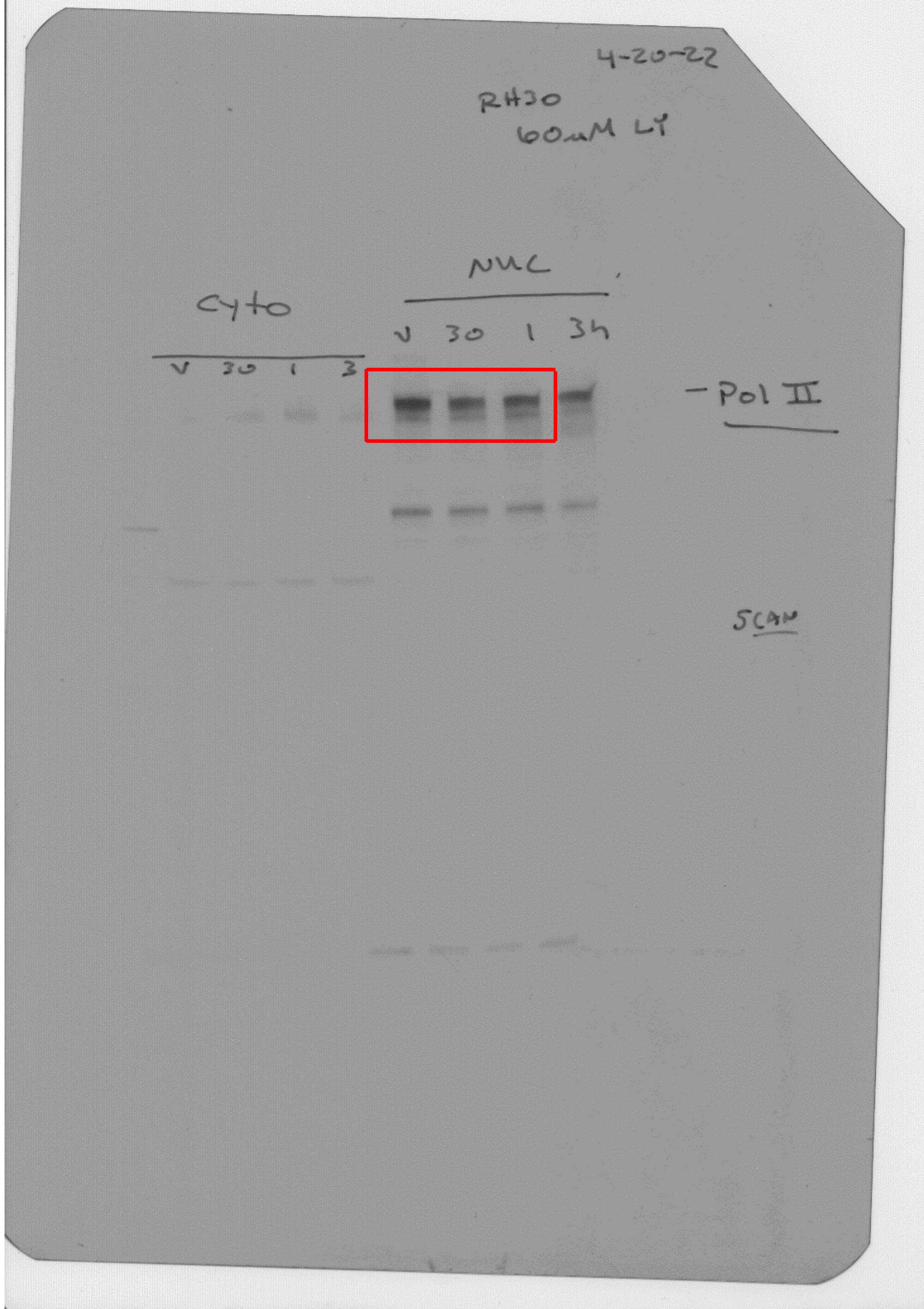

Figure 2F

Ponceau S staining for Gels 1-3

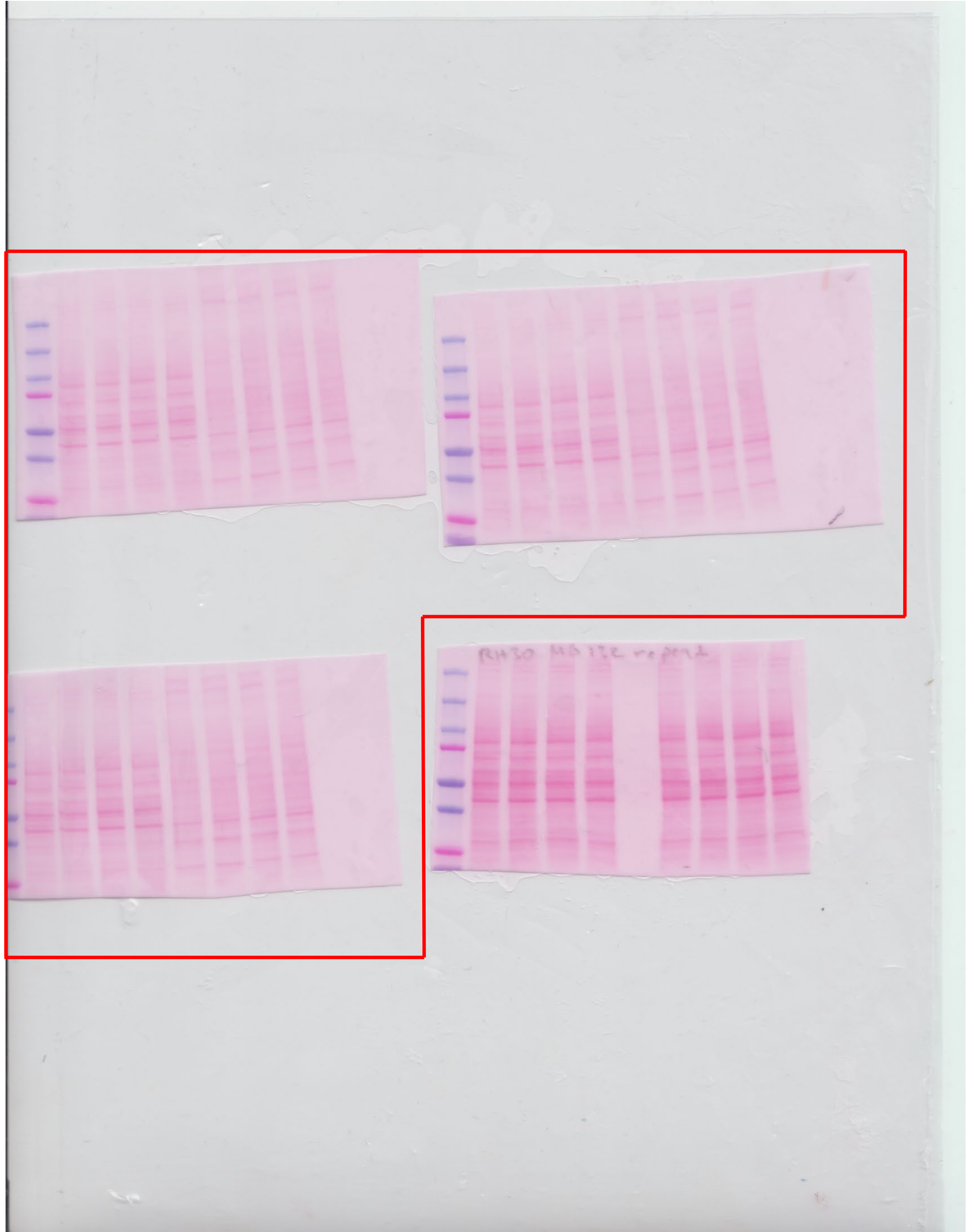

Figure 2G

Gel 1

IB: YAP, cytoplasmic, 65 kDa

IB: TAZ, cytoplasmic, 50 kDa

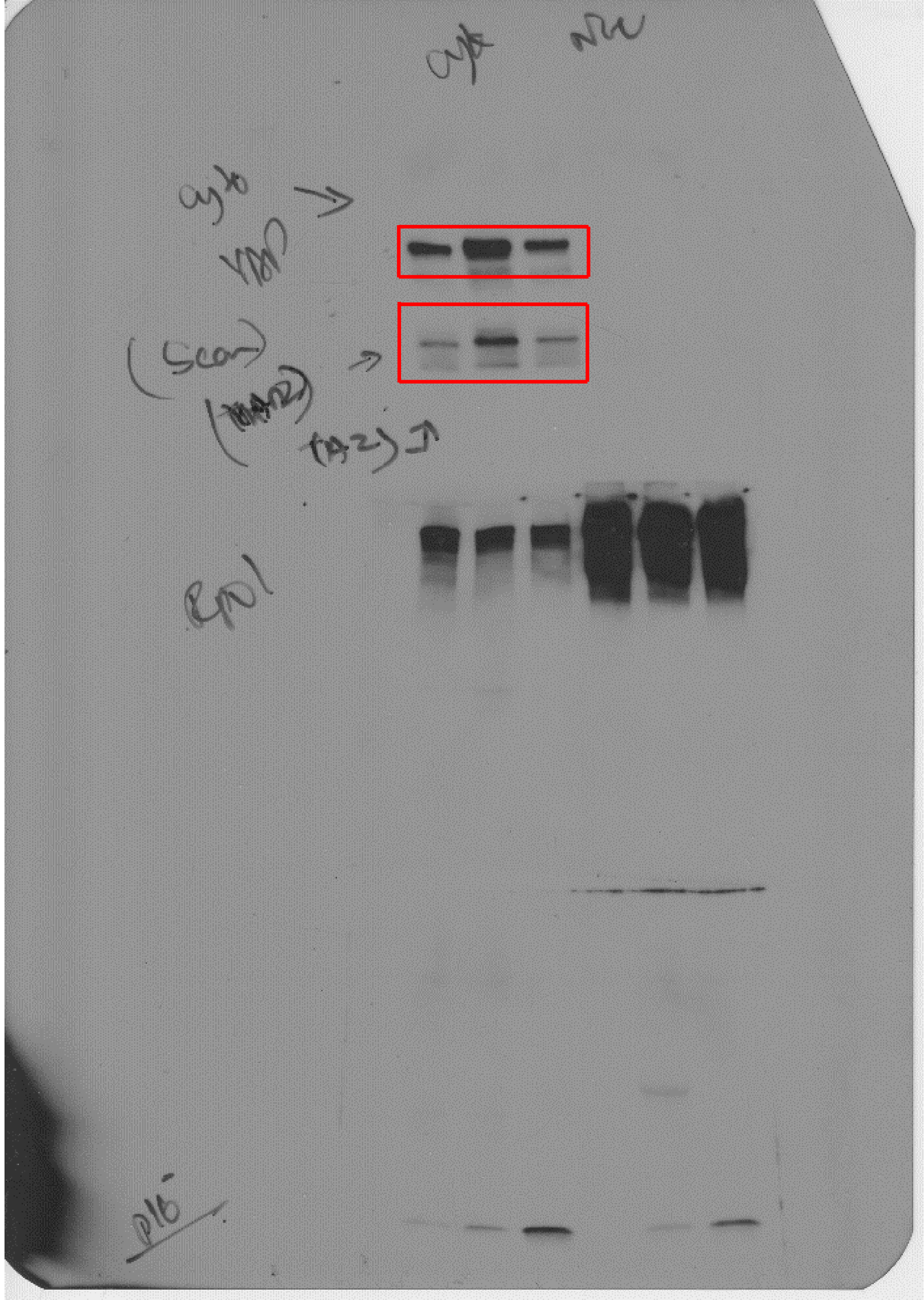

Figure 2G

Gel 1

IB: YAP, nuclear, 65 kDa

IB: TAZ, nuclear, 50 kDa

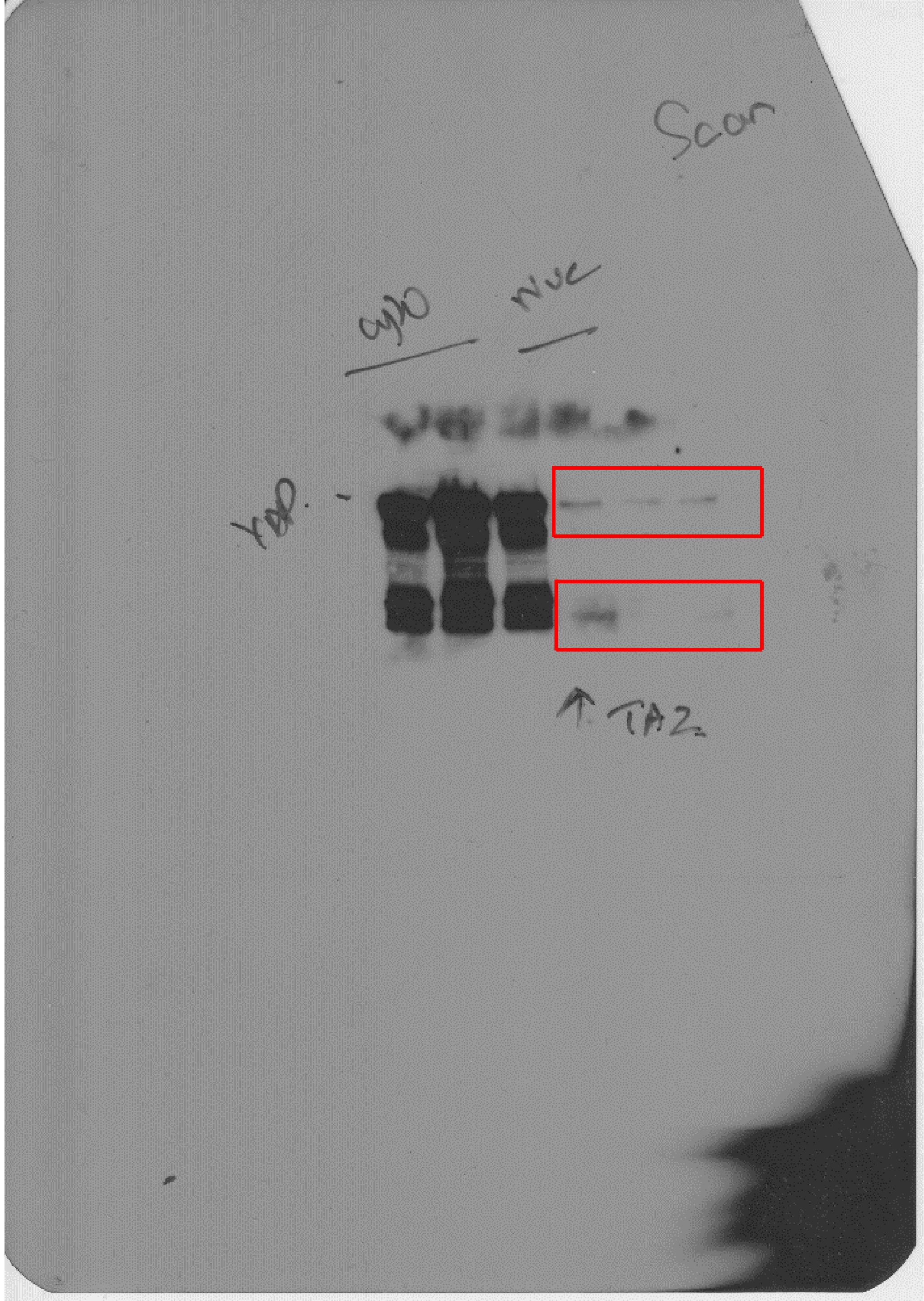

Figure 2G

Gel 1

IB: RNA Pol II 250 kDa

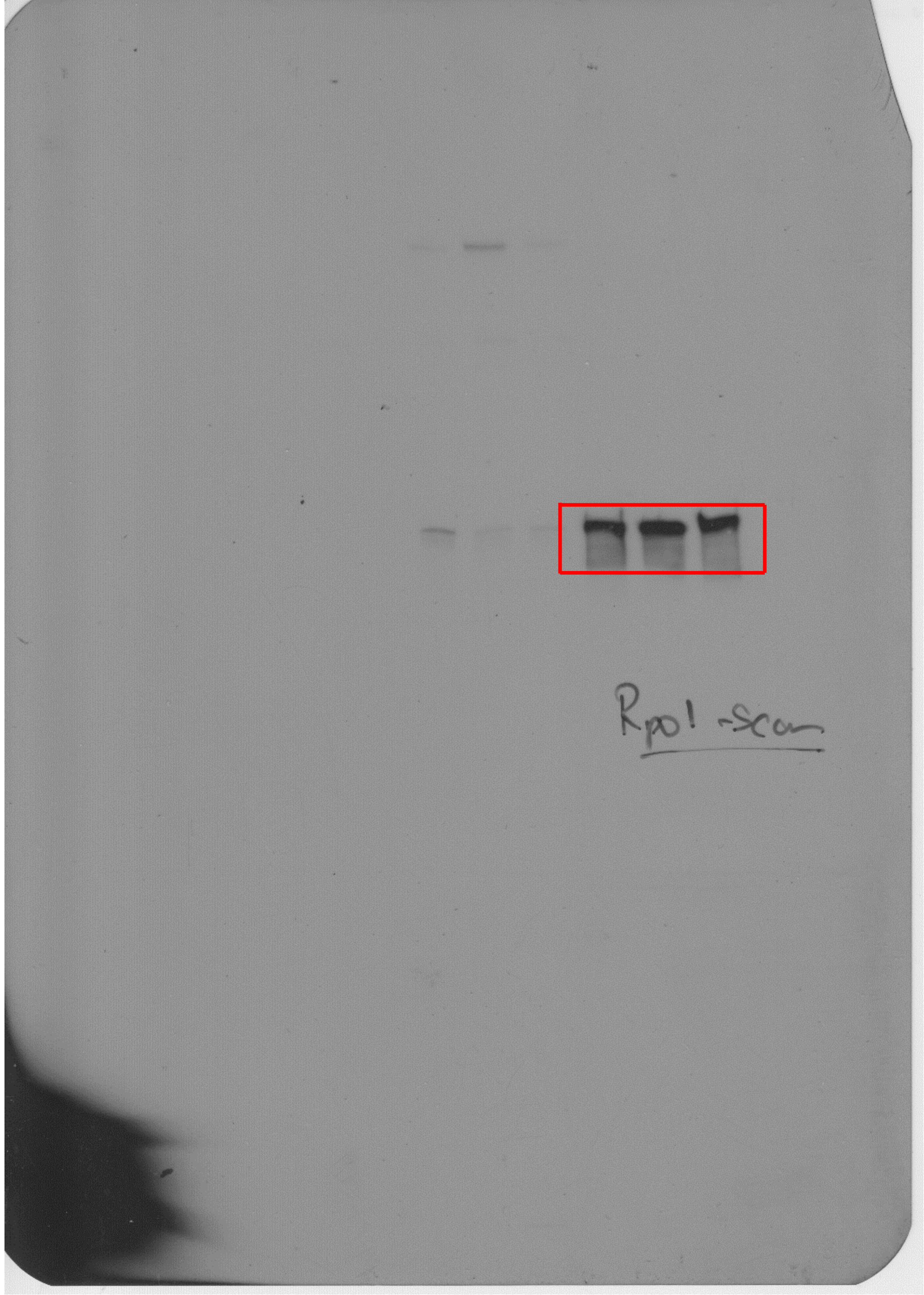

Figure 2G

Gel 2

IB: α – tubulin 50 kDa

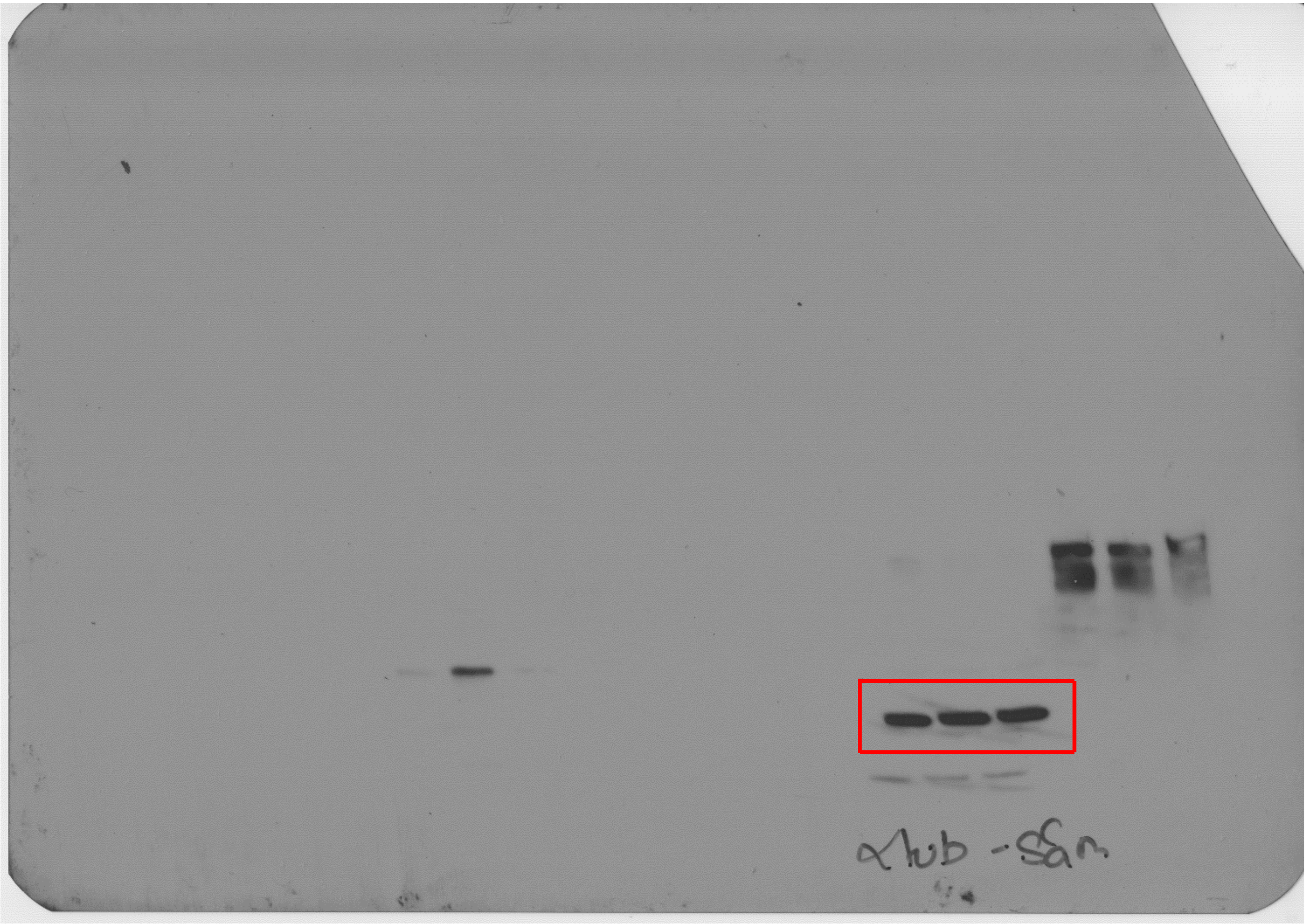

Figure 2G

Ponceau S staining for Gels 1, 2

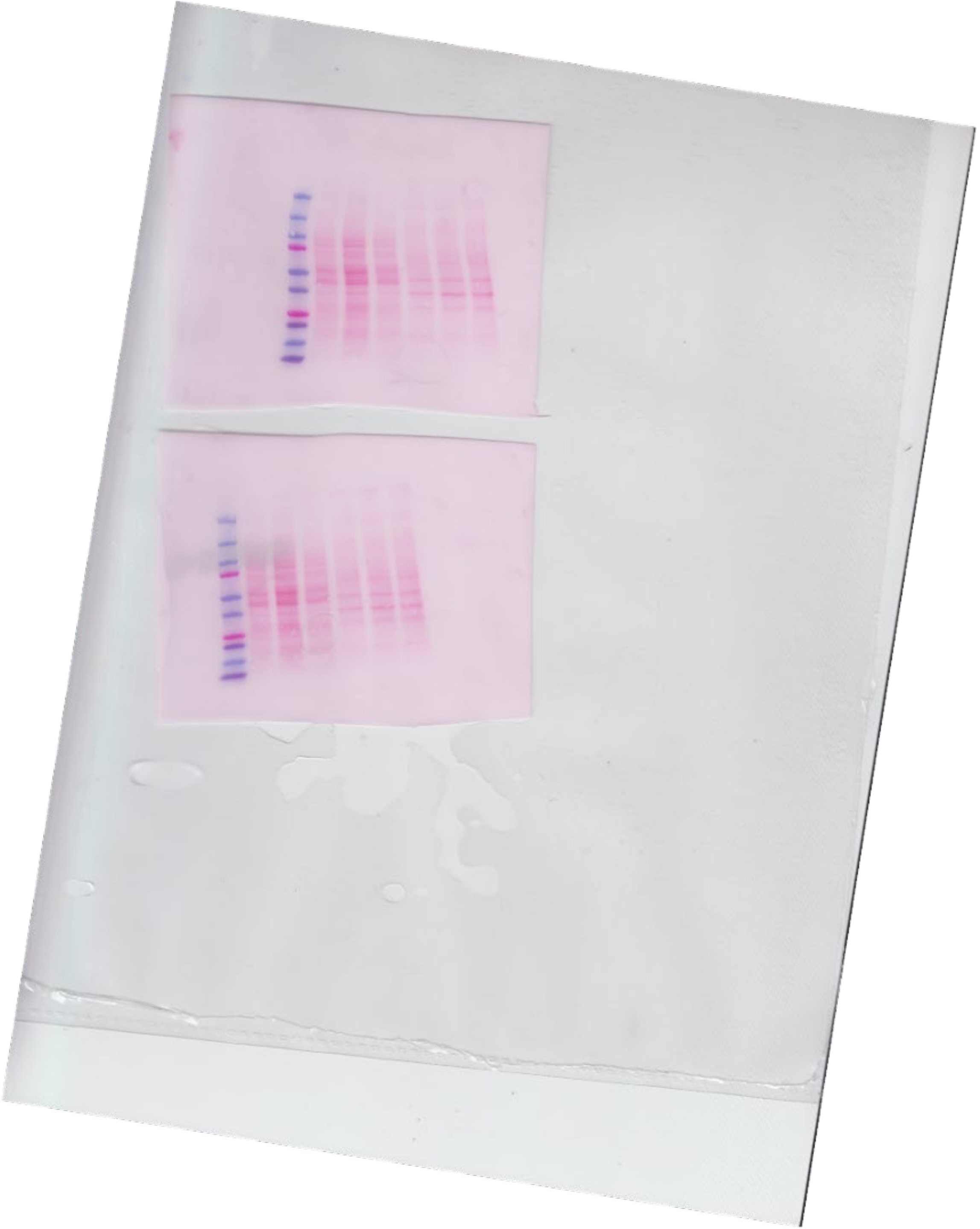

Figure 3B

Gel 1

IB: Flag (Flag-TAZ, 50 kDa)

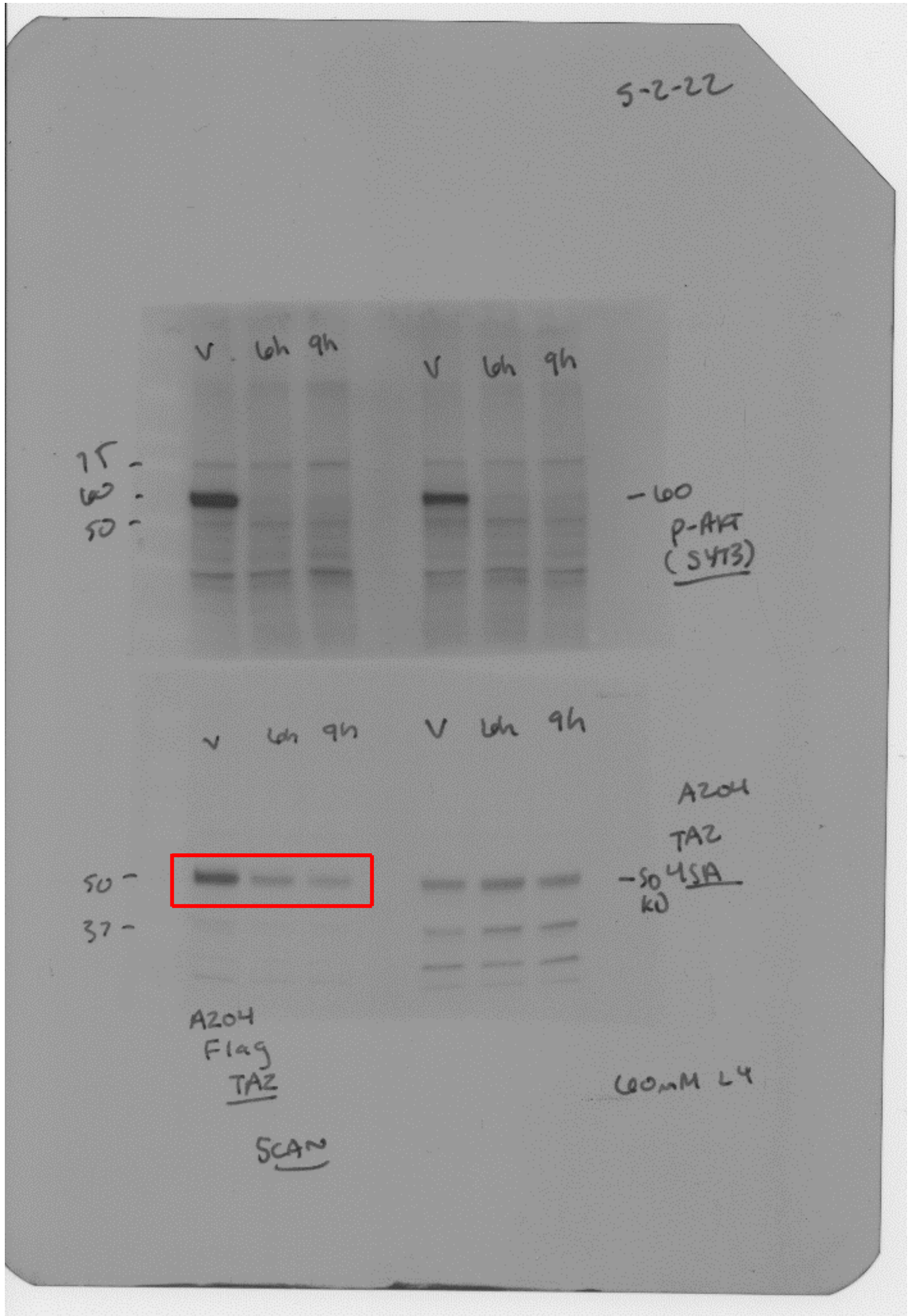

Figure 3B

Gel 1

Residual band from previous immunoblot for Flag-TAZ (slide 12)

IB: β-actin 42 kDa

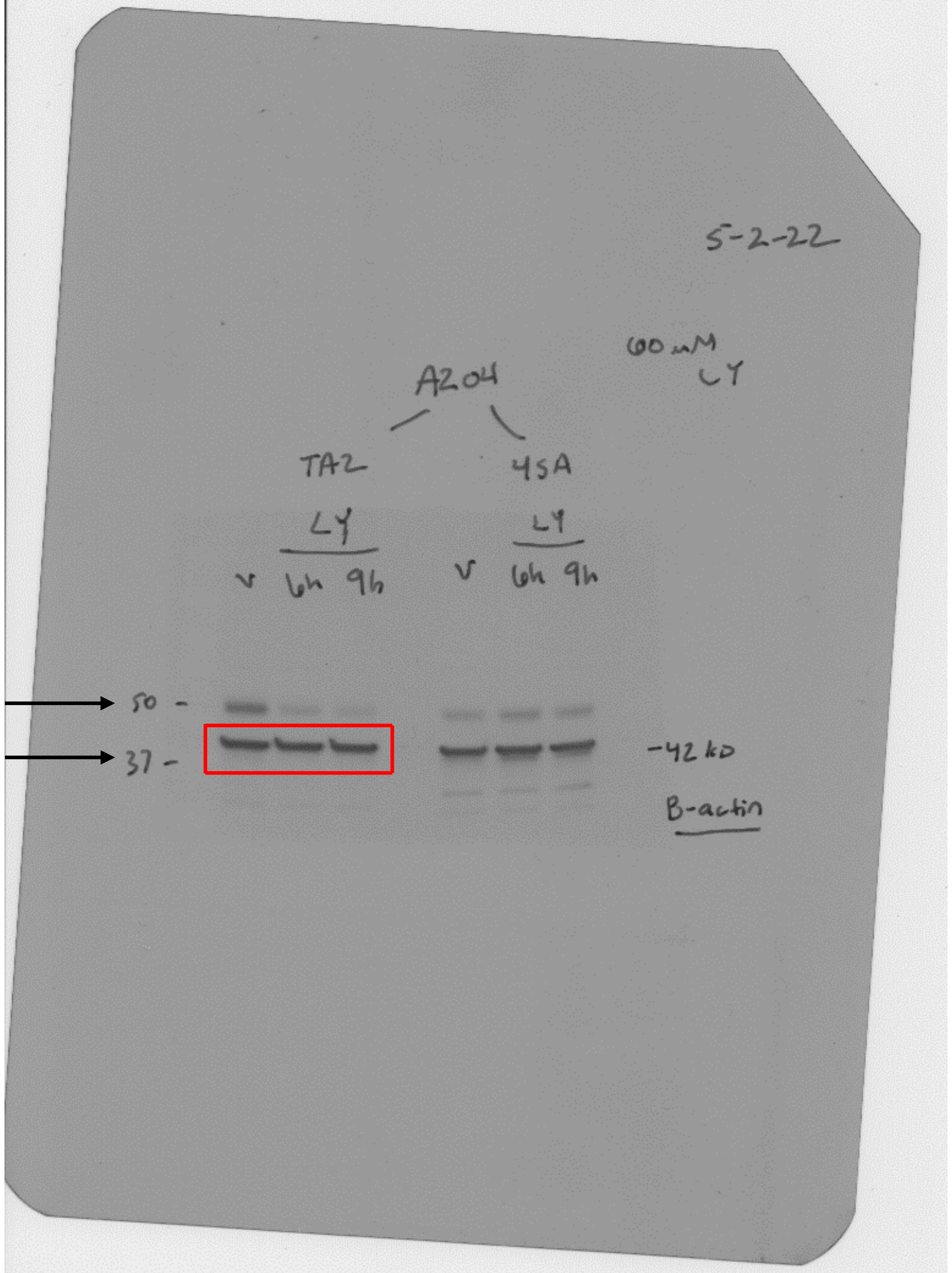

Figure 4A
Gel 2

Slide 14

IB: Yap, 65 kDa

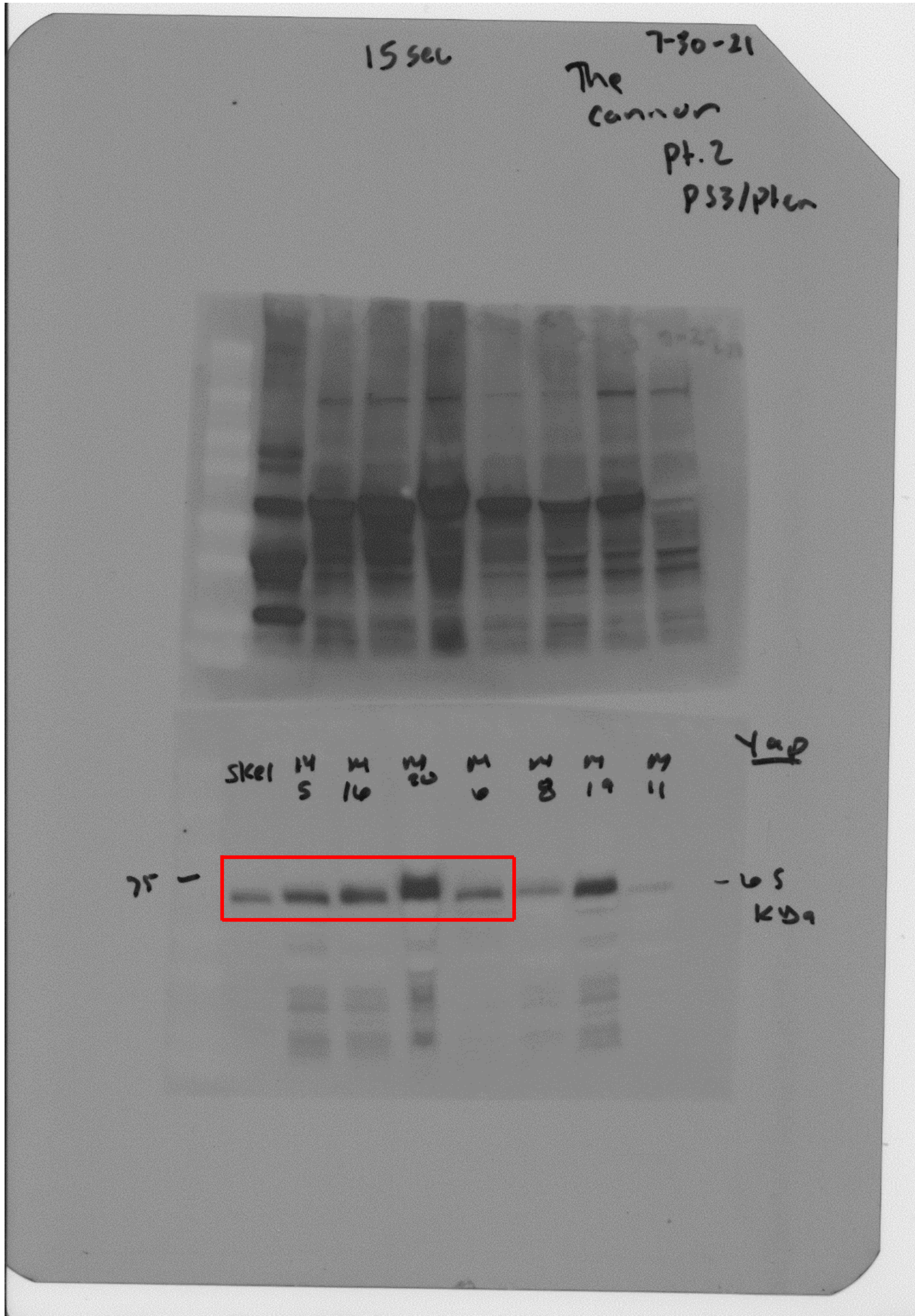

Figure 4A
Gel 2

Slide 15

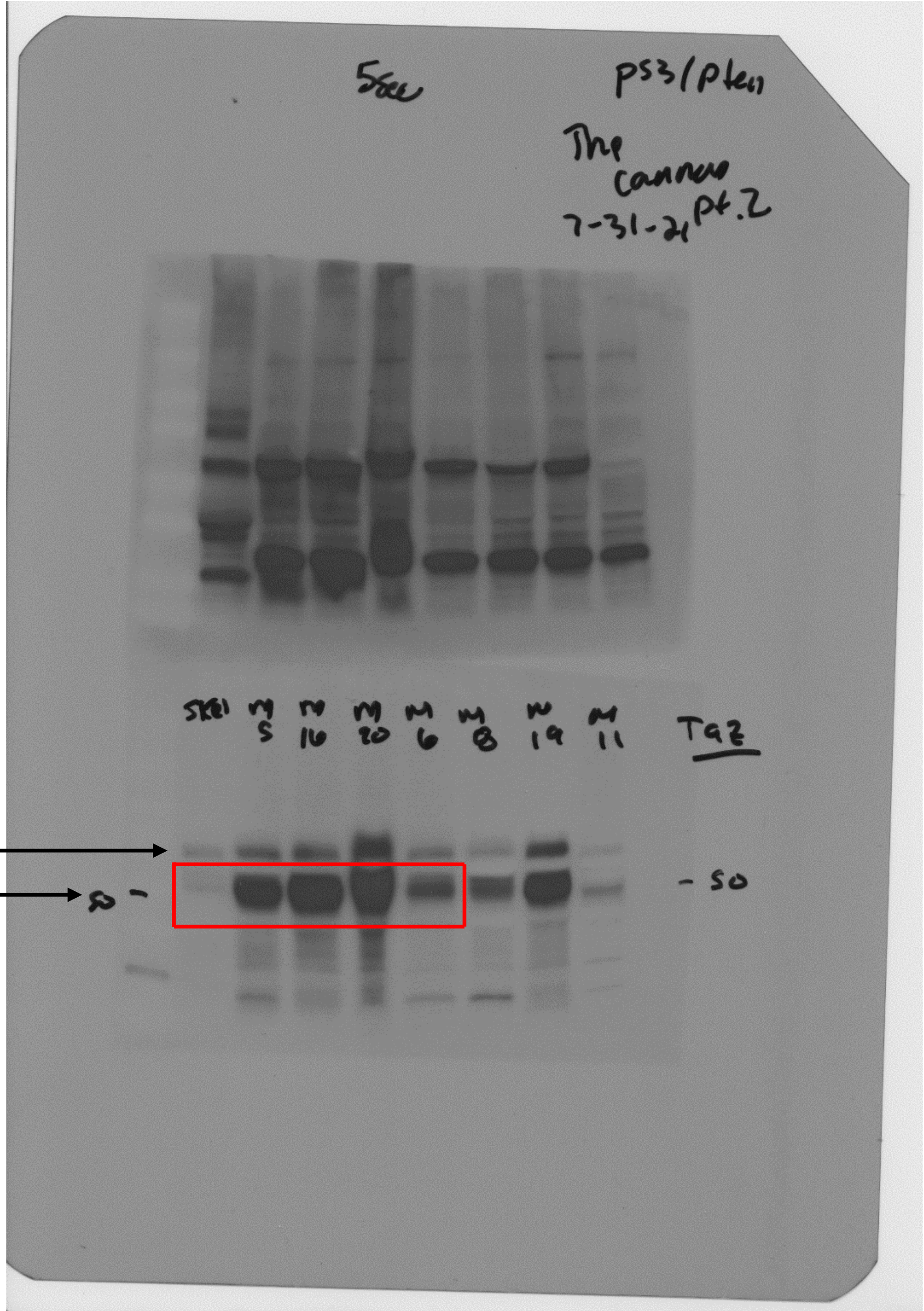

Figure 4A

Ponceau S staining for Gels 1, 2

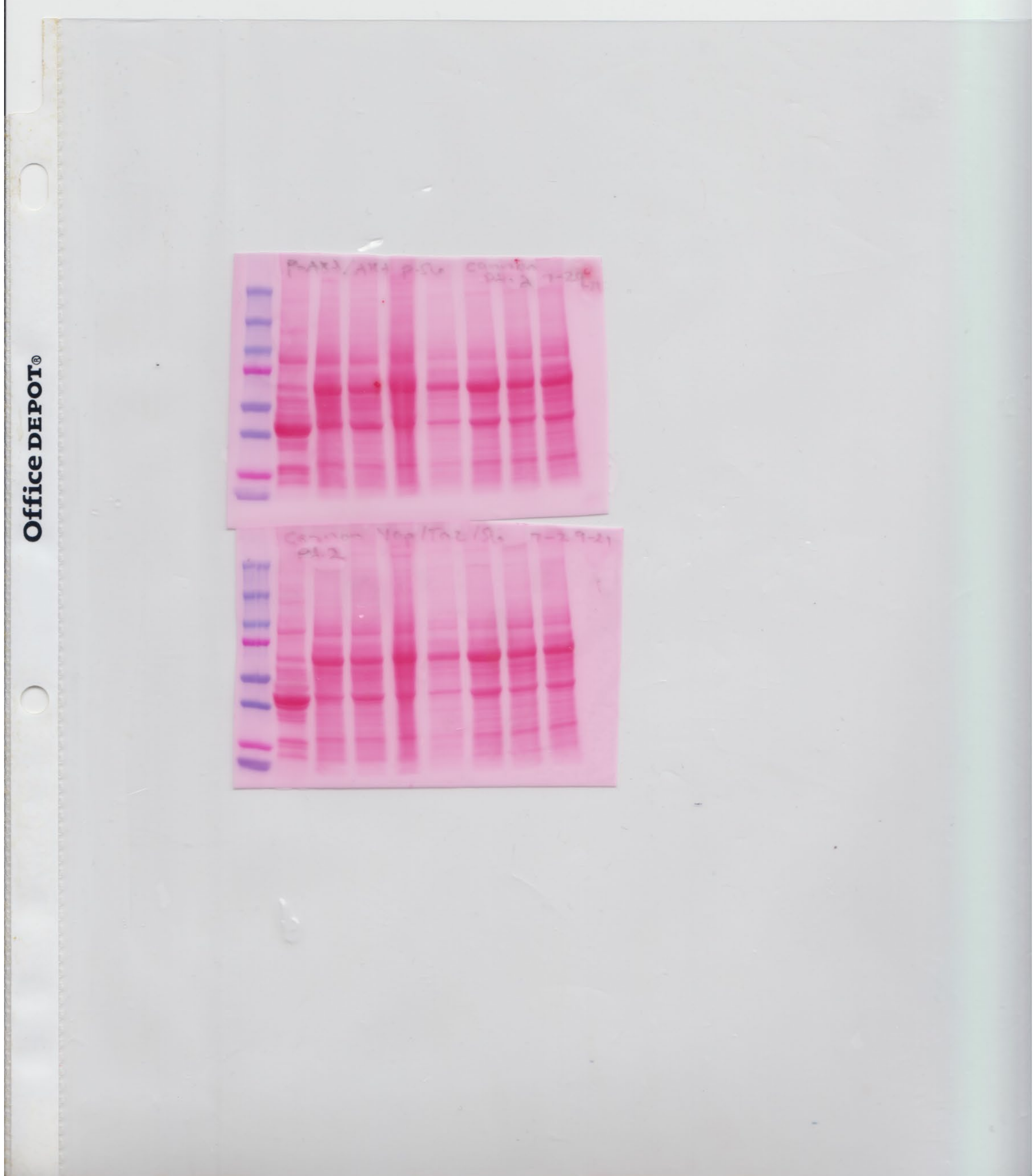

Supplement: Unedited blot and gel images [file jciinsight-11-191600-s374.pdf]
